# ENGEP: advancing spatial transcriptomics with accurate unmeasured gene expression prediction

**DOI:** 10.1186/s13059-023-03139-w

**Published:** 2023-12-21

**Authors:** Shi-Tong Yang, Xiao-Fei Zhang

**Affiliations:** 1 School of Mathematics and Statistics, Central China Normal University, Wuhan, China; 2 Key Laboratory of Nonlinear Analysis & Applications (Ministry of Education), Central China Normal University, Wuhan, China

**Keywords:** Spatial transcriptomics, scRNA-seq, Gene expression prediction

## Abstract

Imaging-based spatial transcriptomics techniques provide valuable spatial and gene expression information at single-cell resolution. However, their current capability is restricted to profiling a limited number of genes per sample, resulting in most of the transcriptome remaining unmeasured. To overcome this challenge, we develop ENGEP, an ensemble learning-based tool that predicts unmeasured gene expression in spatial transcriptomics data by using multiple single-cell RNA sequencing datasets as references. ENGEP outperforms current state-of-the-art tools and brings biological insight by accurately predicting unmeasured genes. ENGEP has exceptional efficiency in terms of runtime and memory usage, making it scalable for analyzing large datasets.

**Supplementary Information:**

The online version contains supplementary material available at 10.1186/s13059-023-03139-w.

## Background

Single-cell RNA sequencing (scRNA-seq) has become a popular tool for analyzing gene expression in individual cells. However, scRNA-seq data loses information about the spatial locations of cells because of the cell dissociation required for sequencing processes. To understand cellular coordination in multicellular organisms, it is important to have access to both gene expression and spatial information [1]. Recently, advances in spatial transcriptomics have made it possible to obtain gene expression and spatial localization data [2]. These methods can be broadly classified into two categories: sequencing-based and imaging-based methods. Sequencing-based methods, such as ST [3], 10X Visium, and Slide-seq [4], can capture the whole transcriptome but are limited by spatial resolution and gene detection sensitivity. On the other hand, imaging-based methods, such as seqFISH [5], osmFISH [6], and MERFISH [2], provide high-resolution and high-sensitivity single-cell gene expression data but are limited to a smaller number of target genes.

In this study, we focus on imaging-based methods due to their high spatial resolution and sensitivity, which are critical for exactly exploring gene expression patterns at the single cell level. Despite the advantages of imaging-based methods, they face challenges in obtaining comprehensive, genome-wide gene expression data [7, 8]. Therefore, users of these methods need to have well-defined biological hypotheses and design an appropriate gene panel, making it unlikely to generate incidental discoveries. To address this challenge, computational methods have been proposed that use scRNA-seq data as a reference to predict the expression of unmeasured genes in imaging-base spatial transcriptomics data, by leveraging the ability of scRNA-seq to provide genome-wide gene expression at the single cell level [9–12].

Most previous methods consist of two steps: searching for neighboring cells in the reference dataset and predicting expression levels. In the first step, different strategies are used to search for neighboring cells in the reference dataset that have similar expression levels to the query cells in the spatial data. For example, Seurat [9] uses canonical correlation analysis to embed both spatial and scRNA-seq datasets into a common low-dimensional space and then identifies the mutual nearest neighbors in this space. SpaGE [10] employs a domain adaptation algorithm to align the spatial and scRNA-seq datasets and then searches for the *k* nearest neighbors based on the aligned datasets. StPlus [11] is based on an auto-encoder deep learning framework and searches for the *k* nearest neighbors based on the learned cell embedding. Tangram [12] uses a deep learning framework to learn a spatial alignment for scRNA-seq data. In the second step, these methods mainly use a weighted average of the expression levels in the identified neighboring cells to predict the expression levels of query cells. The weights can be based on the similarity between the query cell and the neighboring cells, determined using various similarity measures.

The prediction accuracy of previous methods depends primarily on two factors: the quality of the reference dataset used and the prediction methods employed. Firstly, since predictions are made based on the reference dataset, the accuracy of the predictions is significantly influenced by the quality of the reference dataset used. Poor results can arise when the reference dataset contains high levels of noise and batch effects. While there may be multiple reference datasets available for a query dataset, most existing methods use only a single reference dataset, making it challenging to select the optimal one. Secondly, since the accuracy of predictions is determined by the prediction methods used, the choice of prediction methods is critical to achieving high accuracy. Existing prediction methods are often determined by three key components: methods used for correcting batch effects, similarity measures used for searching for neighbors, and the number of selected neighbors. However, in practice, determining the optimal combination of batch effect correction methods [13], similarity measures [14], and number of selected neighbors remains an open problem.

In this study, we propose ENGEP, an ENsemble learning tool for spatially unmeasured Genes Expression Prediction. ENGEP integrates the results of different reference datasets and prediction methods, instead of relying on a single reference dataset or method. It not only avoids manual selection of the best reference dataset and prediction method but also results in a more consistent and accurate prediction. We evaluate ENGEP on three spatial transcriptomics datasets generated by different technologies (MERFISH, osmFISH, and STARmap) and benchmark its performance against five cutting-edge methods. Our findings reveal that ENGEP surpasses alternative methods both in accuracy and in capturing expression patterns, as confirmed by cross-validation. In addition, ENGEP is capable to correct low-quality gene expression and accurately predict the expression patterns of spatially unmeasured genes. We also unveil novel spatial patterns among predicted unmeasured genes, which are distinctly different from the known patterns identified in measured genes. The biological significance of these novel patterns is validated through a comprehensive approach, including comparisons of representative genes within each pattern with corresponding ISH images in Allen Brain Atlas [15], analysis of the co-localization of these patterns with specific cell types, and exploration of biological processes enriched with genes encompassed by each pattern. Finally, we demonstrate that ENGEP requires less computing resources than other methods, rendering it more suitable for handling extensive datasets and broadening its applicability.

## Results

### Overview of ENGEP

**Fig. 1:**
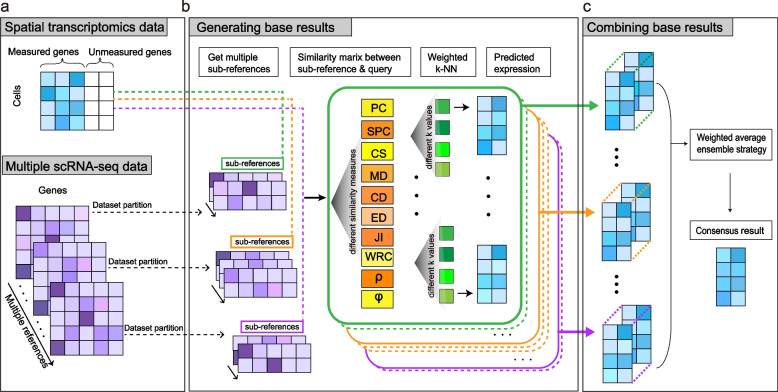
Framework of ENGEP. **a** The input of ENGEP. ENGEP takes in a spatial transcriptomics dataset and multiple scRNA-seq datasets that profile the same or similar tissue as the spatial dataset. **b** Generating base results. ENGEP first partitions each substantial reference dataset into smaller sub-reference datasets. Then, base results are generated by ENGEP for each sub-reference and query dataset pair. It utilizes *k*-nearest-neighbor (*k*-NN) regression with ten different similarity measures and four different values of *k* (number of neighbors) to generate these base predictions. **c** Combining base results. The base results are combined by ENGEP using a weighted average ensemble strategy to produce the final prediction

We present ENGEP, a user-friendly tool for predicting expression levels of spatially unmeasured genes in a query spatial dataset based on ensemble learning. ENGEP achieves high accuracy and robustness by combining multiple prediction results from different reference datasets and prediction methods. The input to ENGEP is a spatial query dataset and multiple sc/snRNA-seq reference datasets collected from the same or similar tissues as the spatial dataset (Fig. 1a). To manage large-scale reference datasets, we first partition each substantial reference dataset into smaller sub-reference datasets. For each sub-reference and query dataset pair, ENGEP uses *k*-nearest-neighbor (*k*-NN) regression with ten different similarity measures and four different values of *k* (number of neighbors) to generate forty different base results (Fig. 1b). The ten similarity measures used, including Pearson correlation coefficient, Spearman correlation coefficient, cosine similarity, Manhattan distance, Canberra distance, Euclidean distance, $\rho _{p}$ measure of proportionality [16], $\phi _{s}$ measure of proportionality [16], weighted rank correlation, and Jaccard index, are complementary to each other and can produce different base results. These ten similarity measures are chosen based on a study [14], which evaluates different association measures in single-cell transcriptomics. We prioritize measures that show strong performance and computational efficiency, making them suitable for integration into ENGEP. The use of four different values of *k* eliminates the need for manual parameter selection and increases the diversity of the base results. To generate the final prediction, ENGEP integrates these base results using a weighted average ensemble strategy, where the weights are assigned to each sub-reference dataset to account for their predictive power (Fig. 1c).

We evaluate performance of ENGEP in two ways (Additional file 1: Fig. S1) by applying it to three datasets generated by different spatial transcriptomics technologies (Additional file 2: Table S1). Firstly, we conduct cross-validation experiments on genes shared by the query and reference datasets. We use three metrics, including Pearson correlation coefficient (PCC), Spearman correlation coefficient (SCC), and root mean square error (RMSE), to evaluate performance. Additionally, we visually compare the spatial patterns of the predicted values with the measured values. To assess ENGEP’s ability to correct for low-quality genes, we compare the predicted patterns with those measured by ISH images from the Allen Brain Atlas or other highly sensitive spatial transcriptomics techniques. Secondly, we use ENGEP to predict the expression levels of spatially unmeasured genes and compare the predicted expression patterns with ISH images. We subsequently apply pattern analysis to ascertain the presence of novel spatial patterns that have not been observed within the spatially measured genes.

### ENGEP accurately predicts unmeasured genes for MERFISH

Multiplexed error-robust FISH [17] (MERFISH) is a spatial transcriptomics technique that enables imaging of hundreds to thousands of RNA species in individual cells by using combinatorial FISH labeling with encoding schemes capable of detecting and correcting errors. However, it faces limitations such as expensive experimental costs, long imaging acquisition time, and complex analysis [18]. To overcome these limitations, predicting the expression levels of genes not measured by MERFISH is valuable. We demonstrate the predictive power of ENGEP on a MERFISH-generated spatial dataset [19] of 254 genes on a section segmented into 3700 cells (“$mouse2\_slice300$”) in the mouse primary motor cortex (MOp). We use seven single-cell (and single-nucleus) RNA-sequencing datasets generated by different techniques from MOp as reference datasets [20] (Additional file 2: Table S1).

**Fig. 2:**
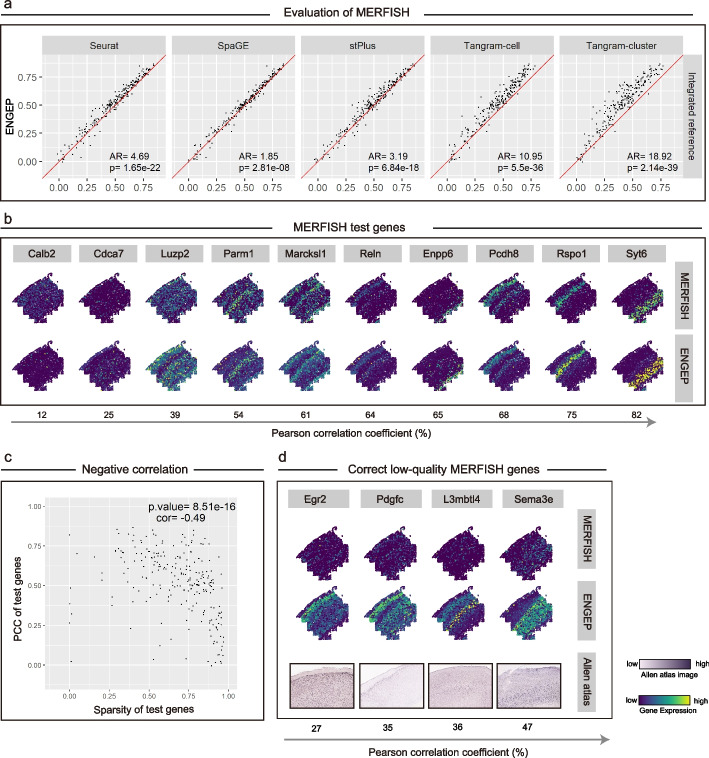
Prediction comparison for MERFISH. **a** Performance comparison between ENGEP and five benchmarked methods via fivefold cross-validation. The scatter plots show the PCC values of every gene across ENGEP (y-axis) and the benchmarked methods (*x*-axis). The red line is the $y=x$ line. The AR value greater than 1 indicates that ENGEP predicts better than others on more genes, while the *P* value (Wilcoxon rank-sum test) shows the significant difference between correlations across two methods. **b** Measured (top) and predicted (bottom) expression patterns of test genes selected with different PCC values (bottom arrow). **c** Negative correlation between the PCC values and sparsity values of each gene. **d** The ability of ENGEP to correct for low-quality genes. The predicted patterns (middle) differ from the measured patterns (top) but are in agreement with the ISH images (bottom)

We conduct a fivefold cross-validation experiment on the 253 common genes shared by the spatial query dataset and seven reference datasets to evaluate performance. We first compare the ensemble result with base results generated from different sub-reference datasets, different similarity measures, and different values of *k* to show the effectiveness of our ensemble strategy. The accuracy of the base results varies significantly with different combinations of sub-reference datasets, similarity measures, and values of *k*. The ensemble result is more accurate than the base results (Additional file 1: Fig. S2). Besides, the significant correlation between the weights assigned to sub-references and the corresponding base results’ performance demonstrates the effectiveness of our weighted ensemble strategy (Additional file 1: Fig. S3, left). Then, to benchmark the performance, we compare ENGEP with five state-of-the-art methods, including Seurat [9], SpaGE [10], stPlus [11], Tangram Cell, and Tangram Cluster [12]. Since all of the compared methods are developed based on a single reference dataset, we merge the seven reference datasets into a single large dataset before running them. ENGEP significantly outperforms the five compared methods (*P* value < 0.05, Wilcoxon rank sum test) in all three evaluation metrics including PCC (Fig. 2a), SCC, and RMSE (Additional file 1: Fig. S4). We further extend our experiments to three additional slices (“$mouse2\_slice50$,” “$mouse1\_slice31$,” and “$mouse1\_slice313$” slices), and the consistently superior performance underscores the generalizability and robustness of ENGEP (Additional file 1: Figs. S5-7). Finally, to evaluate the performance when only one reference dataset is available, we run ENGEP and the five compared methods seven times, each time using only one reference dataset (Methods). The results show that ENGEP still outperforms the five compared methods, even when using only a single reference dataset (Additional file 1: Fig. S8).

In addition to quantitative evaluation, we assess performance in terms of spatial patterns of predicted expression. We select ten genes with varying PCCs from the cross-validation experiment to visually compare the measured and predicted expression levels. Across a broad range of PCCs, expression patterns predicted by ENGEP match well with the measured patterns (Fig. 2b). Moreover, ENGEP is capable of predicting clearer spatial patterns than the five compared methods. For instance, ENGEP faithfully represents the layer structure for the genes *Marcksl*1 and *Reln* and generates clearer spatial patterns for genes such as *Enpp*6 and *Pcdh*8, achievements that some existing methods struggle to replicate (Additional file 1: Fig. S9). We also observe that genes with low PCC values often have sparse expression patterns (Fig. 2c). Despite the low PCC values for genes like *Calb*2 and *Cdca*7, ENGEP still accurately predicts their sparse patterns (Fig. 2b), indicating that lower PCC values do not always indicate poor predictions. We then validate the effectiveness of ENGEP in improving low-quality genes. Although the predicted patterns for genes in Fig. 2d do not match the MERFISH measurements, they are consistent with the ISH images from the Allen Brain Atlas. This suggests that ENGEP is capable of correcting the expression levels of low-quality spatial genes.

**Fig. 3:**
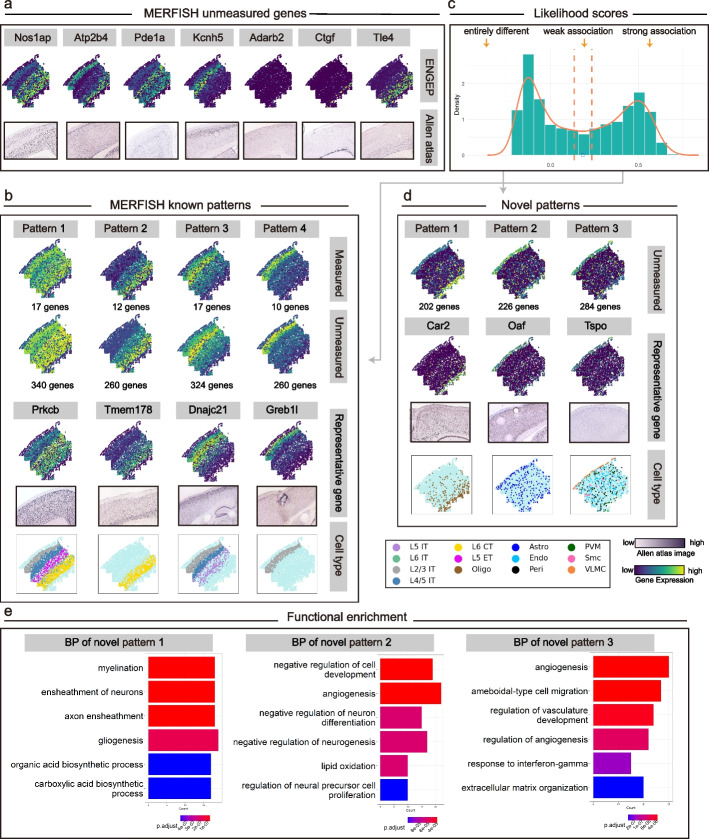
Prediction of expression levels of unmeasured genes for MERFISH. **a** Predicted expression patterns (top) and corresponding ISH images (bottom) of seven spatially unmeasured genes. **b** Known patterns in MERFISH. The first row displays the expression levels averaged by measured genes, while the second row shows the expression levels averaged by unmeasured genes. The number of genes within each pattern is indicated. Representative unmeasured genes for each pattern and their corresponding ISH images are presented in the third row. Each pattern co-localizes with distinct cell types (bottom). **c** Multimodality structure of likelihood scores. Unmeasured genes are categorized into three groups by two dashed lines. The *x*-axis point marked as s (blue point) represents the likelihood score at the low peak between two high peaks. The two dashed lines are located at s ± 0.05. **d** Discovery of three novel expression patterns in MERFISH. The first row illustrates the expression levels of three novel patterns, along with the number of genes composing each pattern. The second row showcases representative genes and their corresponding ISH images. These novel patterns exhibit co-localization with various non-neuronal cell types (bottom). **e** Functional enrichment analysis. The top six biological process-related functional enrichment results are displayed for the three novel patterns

After conducting cross-validation experiments to validate the performance of ENGEP, we utilize it to predict the expression of genes that are not spatially measured but are the union of 2000 highly variable genes in each reference dataset. The predicted expression patterns of the unmeasured genes display clear stratification structures, which are confirmed by the corresponding ISH images (Fig. 3a). One of the genes, *Pde*1*a*, is found to be highly expressed in the L5 ET and L6 CT layers, as confirmed by a previous study that reports low DNA methylation, open chromatin, and strong cell type-specific expression in these layers [20]. Another gene, *Adarb*2, is observed to have a scattered expression pattern and is recognized as a marker gene for Vip/Sncg and Lamp5 [20], which are major subclasses of GABAergic neurons known to have a granular distribution [12]. Additionally, *Ctgf* and *Tle*4 are also found to be marker genes for L6b and L6a, respectively [21]. The validated prediction of expression for these genes, in alignment with existing literature, underscores ENGEP’s competence in offering pivotal biological insights that extend beyond the scope of spatial transcriptomics techniques.

We rigorously analyze the predicted expression profiles of novel spatial patterns not present in the measured gene dataset using a meticulous approach (outlined in the “Methods” section). We employ a graph-based clustering method to group the measured genes, and five known spatial patterns are identified (Fig. 3b, first row, Additional file 1: Fig. S10a). Subsequently, we categorize the predicted unmeasured genes into three distinct groups based on likelihood scores associated with known patterns: genes strongly associated with known patterns, genes weakly associated with known patterns, and genes that significantly deviate from known patterns (Fig. 3c). For genes strongly associated with known patterns, we assign them to their closest matching pattern. It is worth highlighting that none of the predicted unmeasured genes have been conclusively linked to known pattern 5. This could be due to several factors, such as the relatively small number of genes it encompasses compared to the other four patterns, indicating potentially less biological significance. Additionally, there might be noise in the expression profiles of the three constituent genes when compared to the corresponding ISH images (Additional file 1: Fig. S10b). The expression profiles of the remaining four known patterns, computed by averaging unmeasured genes (Fig. 3b, second row), exhibit an impressive alignment with those generated using measured genes (Fig. 3b, first row). Moreover, the representative unmeasured genes assigned to the known patterns closely correspond to the respective ISH images (Fig. 3b, third row), and these patterns exhibit co-localization with various cell types (Fig. 3b, bottom row), providing compelling evidence for the accuracy of ENGEP’s predictions.

We employ a method that encompasses gene filtering, gene clustering, and clustering filtering to unveil novel and biologically significant spatial patterns among genes that deviate from the known patterns. This method successfully identifies three distinct novel patterns, all of which are markedly different from the five known patterns (Fig. 3d, first row). To validate the identified novel spatial patterns, we select representative genes associated with these novel patterns and compare their expression patterns with ISH images. The well match between the expression levels and ISH images of these representative genes indicates the accuracy and feasibility of our predictions (Fig. 3d, second row).

We further investigate the biological significance of these novel spatial patterns by examining their co-localization with specific cell types (Fig. 3d, bottom row, Additional file 2: Table S2) and performing functional enrichment analysis on the genes they encompass (Fig. 3e, Additional file 2: Table S3). Notably, novel pattern 1 demonstrates a co-localization with oligodendrocytes, which primarily serve the vital role of supporting and insulating axons within the central nervous system by forming the protective myelin sheath around them [22, 23]. Our functional enrichment analysis highlights that the genes within novel pattern 1 are intricately associated with various oligodendrocyte-related biological processes, including myelination, ensheathment of neurons, axon ensheathment, oligodendrocyte differentiation, and others. Novel pattern 2 exhibits co-localization with astrocytes, a central cell type in the brain known for its multifaceted roles, including maintaining the blood-brain barrier, supporting neural development and repair, and providing metabolic support [24]. Within novel pattern 2, the genes are enriched in various biological processes that encompass angiogenesis and circulatory system functions, developmental regulation, tissue remodeling and repair, and metabolic processes. This strong alignment between the identified cell type and the enriched biological functions underscores the functional relevance of the genes within this pattern. In contrast, novel pattern 3 co-localizes with a diverse range of non-neuronal cell types, including Endo, SMC, Peri, PVM, and VLMC, all of which collaborate to maintain blood supply and circulation [25, 26]. The top enriched biological processes are also related to the development and maintenance of blood vessels and cell motility and tissue remodeling. It is worth noting that, unlike the neuronal cells that co-localize with the known patterns, the three novel patterns mainly co-localize with various non-neuronal cells. This might be partially attributed to the preponderance of measured genes serving as markers for neuronal cell types, while marker genes for non-neuronal cells are scarce [19]. These findings strengthen our confidence in the biological relevance of the novel spatial patterns, underscoring their potential to unveil previously unexplored biological insights from measured genes.

### ENGEP accurately predicts gene expression in unmeasured targets for osmFISH

OsmFISH [6] is a method for spatial transcriptomics sequencing that uses cyclic single-molecule fluorescence in situ hybridization to detect gene expression. It has a high sensitivity for recovering low levels of gene expression, with a relatively low rate of zero counts [27]. However, it may not be able to assess as many molecular species as other methods [18]. To address this limitation, it is useful to predict spatially unmeasured gene expression for osmFISH data. The osmFISH dataset considered in this study contains information about the expression levels of 33 genes in 3405 cells from somatosensory cortex. Three single-cell RNA sequencing datasets are used as references (Additional file 2: Table S1). The first reference dataset is the Zeisel [28] dataset, which is generated by the same lab and measures the same region as the osmFISH dataset. The second reference dataset is the AllenSSp [29] dataset, which measures the same region with a deep sequencing depth. Finally, the AllenVISp [30] dataset is used as the third reference, which measures a different but similar region with a sequencing depth similar to AllenSSp.

**Fig. 4:**
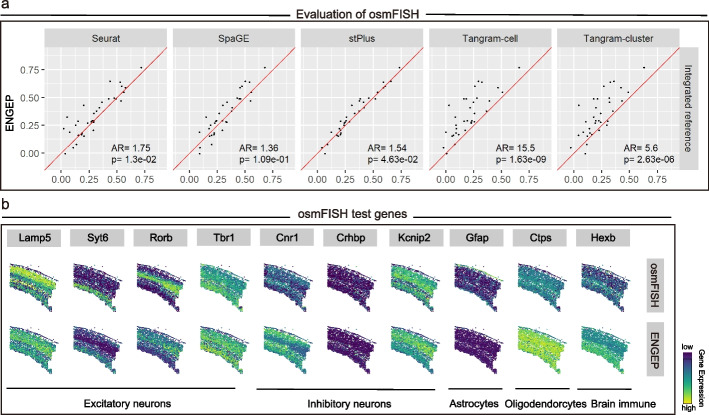
Prediction comparison for osmFISH. **a** Performance comparison via leave-one-out cross-validation. The scatter plots show PCC values of ENGEP (*y*-axis) and benchmarked methods (*x*-axis). In the lower right corner are the AR value and *P* value (Wilcoxon rank-sum test). **b** The expression patterns of the measured genes (top) and the predicted expression patterns (bottom). These genes are marker genes of different cell types

We test the ability of ENGEP on the 33 spatially measured genes using a leave-one-out cross-validation experiment. We find that the ensemble result generated by ENGEP is more accurate than the base results (Additional file 1: Fig. S11). Additionally, the weights assigned to base results are positively correlated with their accuracy (Additional file 1: Fig. S3, middle). These findings provide clear evidence of the effectiveness of our ensemble method. Furthermore, we compare ENGEP with the five other methods, for which we merge the three reference datasets into a single large dataset. Based on the comparison of PCC, as shown in Fig. 4a, ENGEP significantly outperforms the other methods, except for SpaGE (*P* value < 0.05, Wilcoxon rank-sum test). Although the statistical significance may be influenced by the limited number of genes measured, ENGEP produces better predictions on more genes than SpaGE, as the AR value is higher than 1. The results based on SCC and RMSE also demonstrate the superiority of ENGEP (Additional file 1: Fig. S12). When we apply these methods using a single reference dataset, ENGEP still performs better than the other methods that use the same reference (Additional file 1: Fig. S13).

The qualitative evaluation based on expression patterns of the spatially measured genes is also conducted. We visually compare the measured patterns with the predicted patterns and observe that they exhibit highly agreement (Fig. 4b, Additional file 1: Fig. S14a). For instance, the predicted expression patterns of marker genes of excitatory neurons (*Lamp*5 and *Rorb*) display cortical layer patterns, as expected, since excitatory neurons follow a spatial position in the layered structure of the cortex [6]. Additionally, *Gfap* is a marker of type 1 astrocytes derived from layer I, which locates on top of this region [28]. The predicted pattern of *Gfap* also predominantly expresses at the top of the region. In comparison to alternative methods, ENGEP generates expression patterns that are notably clearer and demonstrate a higher degree of similarity to the observed patterns. For example, ENGEP generates clearer spatial patterns for genes such as *Syt*6 and *Cnr*1 and faithfully represents expression patterns for genes *Ctps* and *Hexb*. In contrast, some existing methods encounter challenges in replicating these patterns (Additional file 1: Fig. S14b).

**Fig. 5:**
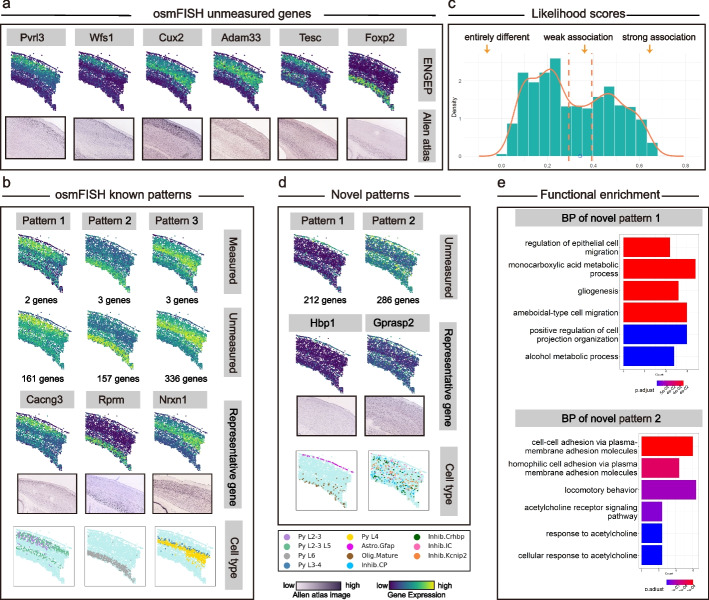
Prediction of expression levels of unmeasured genes for osmFISH. **a** The predicted expression patterns (top) of six spatially unmeasured genes and the corresponding ISH images (bottom). **b** Known patterns in osmFISH. Known patterns, averaged by measured genes, are shown in the first row. Expression levels averaged by unmeasured genes with similar profiles are presented in the second row. The number of genes within each pattern is listed. Representative unmeasured genes for each pattern and their corresponding ISH images are displayed in the third row. Each pattern co-localizes with distinct cell types (bottom). **c** Multimodality structure of unmeasured genes’ likelihood scores. The unmeasured genes are divided into three groups using dashed lines at s ± 0.05, where s (blue point) denotes the likelihood score at the low peak between two high peaks. **d** Discovery of novel patterns in osmFISH. Two novel expression patterns detected in osmFISH are shown in the first row, along with the number of genes constituting each pattern. The second row presents representative genes and their corresponding ISH images. These novel patterns exhibit co-localization with different cell types (bottom). **e** Functional enrichment analysis. The top six biological process-related enrichment results for the two novel patterns are presented

We utilize ENGEP to predict the expression levels of spatially unmeasured genes based on the 33 spatially measured genes. To validate the effectiveness of ENGEP in predicting expression of unmeasured genes, we visually compare the predicted expression patterns with the matching ISH images. Specifically, we choose six marker genes of pyramidal neurons in the mouse somatosensory cortex based on the previously published scRNA-seq data [28]. We discover that the predicted expression patterns of these genes, which are consistent with their ISH pictures (Fig. 5a), clearly show the cortical structures. For instance, *Cux*2 is identified as the restricted molecular marker of upper layer (II-IV) neurons [31] and *Foxp*2 is recognized as the marker of a subpopulation of neurons in layer 6 [32]. The predicted expression patterns of these two genes also show high expression levels in the corresponding layers. By accurately predicting these spatially unmeasured marker genes, ENGEP provides insights into the molecular characteristics and functional roles of these types within the tissue.

We continue our analysis by employing pattern analysis to detect novel spatial patterns within the predicted unmeasured genes. Initially, we utilize a graph-based clustering algorithm to partition the measured genes, successfully identifying six known patterns (Fig. 5b, first row, Additional file 1: Fig. S15). Notably, three of these patterns consist of only one gene, as detailed in Additional file 1: Fig. S15. We then categorize the predicted unmeasured genes into three groups based on their likelihood scores, indicating their association with known patterns (Fig. 5c). For genes closely associated with known patterns, we assign them to the patterns that provide the closest match. In cases where known patterns consist of multiple genes, we observe various predicted genes strongly related to them. However, for the three known patterns that include only one gene, there are no genes exhibiting a strong association with them. This may be attributed to the limitation of having only one gene in these patterns, making it challenging to capture biologically significant spatial patterns (Additional file 1: Fig. S15). Next, we derive the predicted expression profiles for the known patterns by averaging the unmeasured genes (Fig. 5b, second row). These patterns exhibit remarkable consistency with those obtained by averaging the measured genes (Fig. 5b, first row). Additionally, we select representative genes for each known pattern among the unmeasured genes. The predicted expression levels of these genes are validated through ISH images (Fig. 5b, third row). Moreover, these patterns show co-localization with various cell types (Fig. 5b, bottom row).

Moving beyond the known patterns, we identify two novel spatial patterns that distinctly deviate from the established ones (Fig. 5d, first row). Importantly, our predicted expression levels of representative genes consistently match the corresponding ISH images (Fig. 5d, second row). These novel patterns also exhibit co-localization with specific cell types (Fig. 5d, bottom row, Additional file 2: Table S4), and their member genes are significantly enriched in critical biological processes (Fig. 5e, Additional file 2: Table S5). For instance, novel pattern 1 co-localizes with astrocyte gfap and oligodendrocyte mature cell clusters as defined by the authors [6]. Our functional enrichment analysis reveals that its member genes intricately participate in biological processes related to cell migration and metabolic processes [33]. On the other hand, novel pattern 2 co-localizes with various inhibitory neurons (e.g., inhibitory CP, inhibitory Crhbp, inhibitory Kcnip2, and inhibitory IC), which play a fundamental role in maintaining the stability and functionality of the nervous system [34]. Its member genes exhibit enrichment across a range of biological processes, particularly those linked to cell interactions, neural development, and neurotransmission. Notably, the primary enriched processes, shown in Fig. 5e, largely revolve around cell adhesion molecules and acetylcholine. Cell adhesion molecules are instrumental in neural interactions, being expressed by inhibitory neurons to establish and maintain crucial connections necessary for proper neural function and regulation [35]. The influence of acetylcholine in modulating the activity of inhibitory neurons, especially during cerebral cortex functions like motor learning, is underscored by the significant enrichment observed in acetylcholine-related processes [36]. These findings underscore the importance of these novel patterns in untangling complex spatial gene expression and biological processes in the nervous system.

### ENGEP accurately predicts unmeasured gene expression and corrects measured genes for STARmap

Spatially resolved transcript amplicon readout mapping (STARmap) is a cutting-edge technology for 3D intact-tissue RNA sequencing [37]. While this technology is scalable to larger 3D tissue blocks, the current best throughput is only at around 1000 genes, and there is a challenge to sequence all genes simultaneously [27]. Additionally, STARmap has relatively lower gene detection sensitivity, leading to high gene sparsity [10]. To demonstrate the effectiveness of ENGEP on datasets generated by this technology, we utilize a STARmap dataset [37] comprising 1020 genes and 973 cells from a mouse brain slice from the visual area (VISp). The same three references as those for the osmFISH dataset are used.

**Fig. 6:**
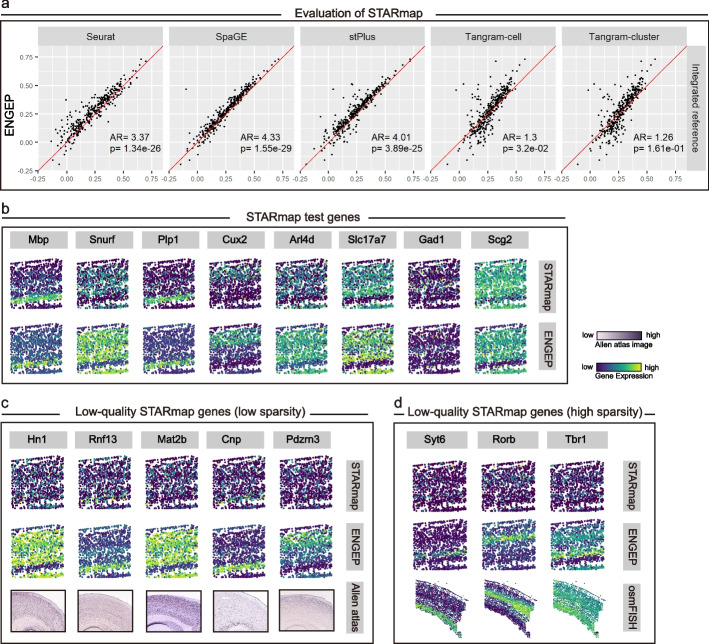
Prediction comparison for STARmap. **a** Performance comparison via fivefold cross-validation. ENGEP (*y*-axis) predicts expression of measured genes (with low sparsity) and shows its superiority to benchmarked methods (*x*-axis) by comparing PCC values. The AR values and *P* values are also reported. **b** The predicted expression patterns of measured genes (top) and the measured patterns of them (bottom). **c** Correction of low-quality genes (with low sparsity). Although the predicted patterns (middle) of these genes differ from the measured ones (top), they agree well with the ISH images (bottom). **d** Correction of low-quality genes (with high sparsity). The predicted patterns (middle) of these high sparsity genes are mismatched with the measured patterns of them (top) but are in agreement with patterns measured by osmFISH (bottom), a spatial transcriptomics technology with high sensitivity

A fivefold cross-validation is conducted to quantitatively evaluate the performance of different methods. Considering the substantial gene sparsity inherent in STARmap data, which has the potential to result in inaccurate measurements and influence performance evaluation, we choose to focus on 342 measured genes characterized by a gene sparsity below 0.7 for the purpose of the cross-validation experiment. ENGEP outperforms all other base predictions based on the mean PCC values (Additional file 1: Fig. S16). Our ensemble strategy’s effectiveness is also demonstrated by the positive correlation between the weights assigned to base predictions and their performance (Additional file 1: Fig. S3, right). In addition, the five compared methods are evaluated using a large dataset merged from the three reference datasets. As shown in Fig. 6a, ENGEP significantly outperforms the compared methods, except for Tangram-cluster, in terms of PCC (*P* value < 0.05, Wilcoxon rank-sum test). Although the difference is not significant, ENGEP still performs better than Tangram-cluster on more genes since the AR value is higher than 1. Moreover, based on the SCC and RMSE measurements, ENGEP exhibits better performance than the compared methods (Additional file 1: Fig. S17). Finally, ENGEP and the five benchmarked methods are evaluated using a same single reference dataset. ENGEP still exhibits superior performance compared to the benchmarked methods (Additional file 1: Fig. S18).

ENGEP can accurately capture expression patterns of spatially measured genes (Fig. 6b). Notably, ENGEP’s predicted expression patterns agree better with the measured patterns than the compared methods. For example, ENGEP generates clearer spatial patterns of *Mbp*, a representative marker for oligodendrocytes cells [37]. ENGEP also accurately predicts the expression patterns of genes *Snurf* and *Plp*1. In contrast, some existing methods fail to capture the correct patterns of these genes, with a few even producing entirely contradictory patterns compared to the measured ones (Additional file 1: Fig. S19). Moreover, ENGEP can also correct expression patterns for low-quality genes. For instance, although the predicted patterns of the genes shown in Fig. 6c are inconsistent with the measured patterns, they are consistent with the ISH images, confirming ENGEP’s ability to correct expression. Additionally, ENGEP can correct expression patterns for high sparsity genes (gene sparsity higher than 0.7) that are filtered out in the cross-validation experiment. Although the predicted expression of *Rorb*, *Syt*6, and *Tbr*1 are not consistent with the measured patterns, they are in agreement with the patterns measured by osmFISH [6], a high detection sensitivity technique that profiles the similar region as STARmap (Fig. 6d).

**Fig. 7:**
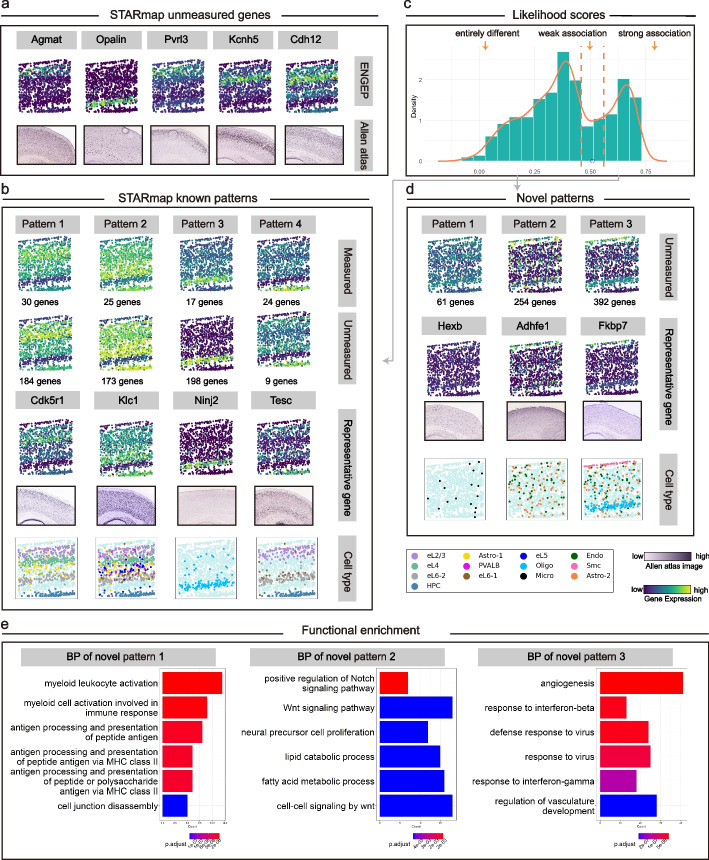
Prediction of expression levels of unmeasured genes for STARmap. **a** Predicted expression patterns of five unmeasured genes (top) and their ISH images (bottom). **b** Known patterns in STARmap. The first row shows the expression levels of known patterns, averaged by measured genes. The second row presents expression levels averaged by unmeasured genes. The number of genes within each pattern is listed. The third row showcases representative unmeasured genes and their corresponding ISH images. These patterns co-localize with distinct cell types (bottom). **c** Multimodality structure of likelihood scores of unmeasured genes. These genes can be divided into three categories using dashed lines at s ± 0.05, where s (blue point) represents the likelihood score at the low peak between two high peaks. **d** Discovery of three novel patterns in STARmap. The first row illustrates the expression levels of three novel patterns, along with the number of genes constituting each pattern. The second row displays representative genes and their corresponding ISH images. These novel patterns demonstrate co-localization with different cell types (bottom). **e** Functional enrichment analysis. Functional enrichment results are provided (top six) for the three novel patterns

Based on the selected 342 low sparsity genes, we use ENGEP to predict the expression levels of spatially unmeasured genes. We visually evaluate the accuracy of these predictions by comparing them to corresponding ISH images (Fig. 7a). The predicted expression patterns show cortical structures and match well with the ISH images. For example, the marker gene for subclasses of L2/3 IT from the VISp region [30], *Agmat*, is accurately predicted by ENGEP. Similarly, *Opalin*, a marker gene of oligodendrocyte subtypes [38], is also predicted to have high expression levels in the area of oligodendrocytes. Besides, *Kcnh*5, a marker gene of L4 neurons [39], is also well predicted by ENGEP. By accurately predicting these marker genes, ENGEP helps to understanding the cellular heterogeneity present in the tissue.

To confirm the presence of novel spatial patterns among the predicted unmeasured genes, we initiate the process by clustering the spatially measured genes, which leads to the identification of five known patterns (Fig. 7b, first row, Additional file 1: Fig. S20a). Subsequently, we calculate likelihood scores associated with the known patterns for the unmeasured genes (Fig. 7c), resulting in the categorization of genes into three distinct groups. Genes closely associated with the known patterns are then assigned to their corresponding counterparts. It is worth noting that no predicted unmeasured genes are conclusively linked to known pattern 5 (Additional file 1: Fig. S20a). This could be due to several factors. Firstly, when analyzing the measured expression levels of the four constituent genes within known pattern 5, we observe that they lack clear layer structures, unlike the other four known patterns, and do not closely match the ISH images. Additionally, known pattern 5 consists of significantly fewer genes compared to the other four known patterns, indicating its potentially lower biological significance (Additional file 1: Fig. S20b). When we average the predicted expression of the unmeasured genes designated to known patterns, we observe a harmonious consistency compared to the expression averaged from the measured genes (Fig. 7b, second row). Within each pattern, we identify representative genes from the pool of unmeasured genes, and their predicted expression levels align with ISH images (Fig. 7b, third row). Furthermore, the regions of elevated expression for these known patterns exhibit co-localization with various cell types (Fig. 7b, bottom row).

We proceed to cluster genes that exhibit distinct patterns differing from the known ones, and three novel patterns are identified (Fig. 7d, first row). Subsequently, we select representative genes for each novel pattern and confirm their expression patterns through ISH images (Fig. 7d, second row). These novel patterns are also notable for their widespread expression across various cell types (Fig. 7d, bottom row, Additional file 2: Table S6) and their involvement in critical biological processes (Fig. 7e, Additional file 2: Table S7). Novel pattern 1 closely associates with microglia, the central nervous system’s primary immune cells [40]. Examination of the genes within this pattern reveals significant enrichment in immune-related functions, including immune response regulation, antigen processing and presentation, and cell-mediated immune responses. Novel pattern 2 co-localizes within both astrocytes and endothelial cells, highlighting their collaborative role in the central nervous system. Genes in this pattern are intricately involved in signaling pathways, cell fate determination, proliferation, angiogenesis, and blood-brain barrier integrity [41, 42]. In addition to the two cell types associated with novel pattern 2, novel pattern 3 also co-locates with smooth muscle cells and oligodendrocytes. Smooth muscle cells and endothelial cells often collaborate within blood vessel tissues to regulate vascular tone and diameter [43]. On the other hand, oligodendrocytes and astrocytes, both present in the central nervous system, work together to support neuronal functions and provide protection [33]. Astrocytes can also interact with endothelial cells, influencing blood vessel function and the regulation of the blood-brain barrier [41, 42]. Genes within this pattern are enriched with various biological processes, including those related to angiogenesis and vascular development, immune responses, cell adhesion and migration, viral processes, cytokine signaling, and cellular proliferation and morphogenesis. These patterns shed light on novel aspects of cellular interactions and functions within the central nervous system.

### ENGEP exhibits computational efficiency in terms of both time and peak memory usage

**Fig. 8:**
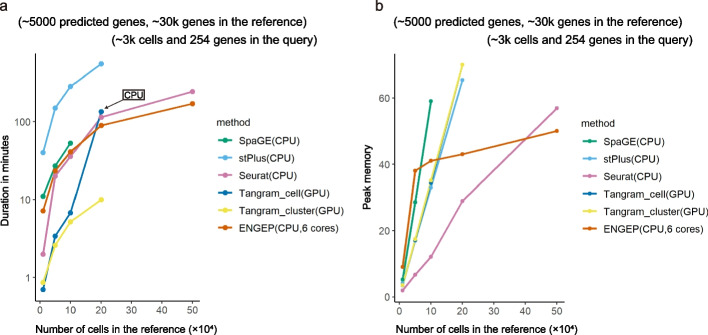
Comparison of running time and peak memory. Running time (**a**) and peak memory (**b**) of different methods. We use different methods to predict 5000 unmeasured genes in spatial dataset containing 3700 cells and 254 genes with reference datasets comprising 30,000 genes and varying numbers of cells, specifically, 10,000, 50,000, 100,000, 200,000, and 500,000 cells. We run both Tangram-cell and Tangram-cluster on our GPU platform, except for when we run Tangram-cell with a reference containing 200,000 cells. In that case, we encounter a memory error while using GPU, so we switch to using CPU instead

To facilitate a comprehensive comparison of computational resources, we conduct benchmarking tests to evaluate the execution time and peak memory utilization of different methods. We utilize references from the Brain Image Library (used in the MERFISH experiment) to predict the expression of 5000 unmeasured genes within the MERFISH dataset. We sample cells and generate reference datasets comprising 30,000 genes with varying numbers of cells, specifically 10,000, 50,000, 100,000, 200,000, and 500,000 cells, to assess the impact of cell numbers on reference datasets. We employ a research server equipped with an Intel(R) Xeon(R) Silver 4214 CPU (48 cores and 128GB of memory) and a Tesla V100-PCIE-16GB GPU (CUDA Version 11.4) to execute all methods. Notably, Tangram supports GPU processing, so we run both Tangram-cell and Tangram-cluster on our GPU platform. ENGEP supports multi-core parallel operation, utilizing six cores when run on the CPU platform. Other methods are executed using a single core on the CPU platform. Figure 8 shows that only Seurat and ENGEP can predict with large references containing 500,000 cells and 30,000 genes. It is noteworthy that ENGEP is the most computationally efficient method, as its execution time and peak memory usage increase gradually with the rise in the number of cells.

## Discussion

Recent advances in imaging-based spatial transcriptomics have enabled the mapping of gene expression and spatial localization at single-cell resolution, allowing the study of spatially organized patterns of gene expression in tissues. However, a major limitation of these techniques is the number of genes that can be measured. To address this issue, we have developed a new computational tool, ENGEP, that predicts the expression levels of spatially unmeasured genes. We have conducted extensive experiments to evaluate the performance of ENGEP, and the results demonstrate that it is a highly accurate, scalable, fast, and memory-efficient tool. ENGEP’s superior performance positions it as an ideal choice for uncovering previously untapped biological insights within spatial transcriptomics data. Moreover, by applying ENGEP to predict the expression levels of spatially unmeasured genes, we uncover unique spatial expression patterns that differ from those captured by spatially measured genes, offering profound biological significance.

In contrast to their predecessors, such as Seurat, SpaGE, stPlus, Tangram Cell, and Tangram Cluster, ENGEP addresses the limitations of limited robustness and variable performance across datasets by embracing an ensemble learning approach. Previous tools rely on singular reference datasets and prediction methods, which result in disparities in their effectiveness. For example, SpaGE excels in MERFISH and osmFISH datasets but struggles with STARmap, while Tangram Cell and Tangram Cluster display competitive performance on STARmap but falter on the other datasets. In contrast, ENGEP integrates a diverse array of reference datasets and prediction methods through ensemble learning, including *k*-NN regression employing various similarity metrics and *k* values. This multifaceted strategy obviates the need for selecting an optimal combination of reference datasets, similarity measures, and *k* values, resulting in predictions that are not only more robust but also notably accurate. Furthermore, ENGEP is designed to be user-friendly, which means it does not require meticulous parameter tuning.

Throughout our preceding experiments, we underscore the efficacy of our ensemble approach, consistently observing superior performance from ensemble results compared to base results. We further delve into the impact of the number of references on outcomes through a series of experiments (Additional file 1: Section S3.1). Our findings reveal that datasets with fewer references (e.g., osmFISH and STARmap) invariably benefit from an increasing number of references. Conversely, datasets with a substantial number of references (e.g., MERFISH) exhibit an initial performance ascent followed by saturation (Additional file 1: Fig. S21). Furthermore, we conduct analyses on the ten measures employed in this study to ascertain their consistent performance [44] in both accuracy and runtime (Additional file 1: Section S3.2). We find no singular similarity measure that universally outperforms others across all datasets. This underscores the fundamental necessity of amalgamating results emanating from diverse reference and prediction methods, reinforcing the paramount value of our ensemble approach.

Batch effect correction is widely used in previous tools for searching for neighbors in the reference for query cells. However, in this study, we do not consider batch effect correction for several reasons. Firstly, it is unclear which batch effect correction method is most suitable for this task. Secondly, using an inappropriate batch correction method can lead to over-correction and produce misleading results. Thirdly, some batch correction methods are time-consuming and require a large amount of memory, which would increase the time and memory cost of ENGEP. What is more important, our experiment results have demonstrated that even without using batch effect correction, ENGEP outperforms the five methods that carefully design batch effect corrections. Therefore, we focus on developing a robust and accurate method that avoids the complexity and potential errors associated with batch effect correction.

The current version of ENGEP is focused on predicting gene expression levels of unmeasured genes using shared genes between query and reference datasets, without accounting for the spatial arrangement of cells within the query data. Recent research has illuminated a structural correspondence between cell distances in expression space and their physical locations, underscoring the importance of incorporating spatial data for accurate gene expression prediction [1, 45]. We plans to enhance ENGEP by integrating cell spatial information. Furthermore, innovative studies like CSOmap [45] have unveiled that cellular spatial organization is orchestrated through ligand-receptor interactions, which can be leveraged to reconstruct cell spatial organization from scRNA-seq data. Novel methodologies can be devised to incorporate this spatial information into ENGEP’s framework. Moving forward, after generating predictions for spatially unmeasured genes, an array of downstream analyses can be undertaken. These include exploring spatial gene expression patterns, conducting spatial trajectory inference, investigating cell-cell communication patterns, and constructing spatially resolved gene regulatory networks, among others. While our current study has primarily centered on exploring spatial gene expression patterns, our future research endeavors aim to expand ENGEP’s capabilities to encompass a diverse array of additional downstream analyses guided by the predictions generated by our ensemble methods.

## Conclusions

In conclusion, our study introduces ENGEP, an accurate and scalable tool for predicting gene expression levels of spatially unmeasured genes. By leveraging an ensemble learning strategy that integrates multiple reference datasets and prediction methods, ENGEP outperforms existing state-of-the-art tools. ENGEP also enables the detection of novel patterns that are unknown in the measured genes. This highlights the importance of predicting spatially unmeasured genes. Our results demonstrate the efficiency of ENGEP for gene expression prediction and its potential for advancing the field of spatial transcriptomics.

## Methods

### ENGEP algorithm

ENGEP is designed to predict the expression of genes that are not present in the spatial (query) dataset but are present in multiple scRNA-seq (reference) datasets. To achieve this, ENGEP takes as input a set of gene expression matrices from the reference datasets, as well as a gene expression matrix from the query dataset. The method consists of two main steps: (i) generating multiple base results using *k*-nearest-neighbor (*k*-NN) regression; different reference datasets, similarity measures, and numbers of neighbors (*k*) are used for this step; and (ii) combining these base results into a consensus result using a weighted average ensemble strategy. In this step, weights are assigned to different reference datasets to take into account their predictive power.

#### Step 1: Generating base results

ENGEP generates multiple base results using *k*-NN regression from three perspectives: (i) different reference datasets, (ii) different similarity measures, and (iii) different values of *k*.

Different sequencing technologies have their own strengths and weaknesses [46, 47]. To account for data quality variations, ENGEP utilizes multiple reference datasets for predicting gene expression levels of unmeasured genes. Specifically, ENGEP assumes that several scRNA-seq datasets profiling the same or similar region and sharing common genes with the spatial dataset are available. To adapt ENGEP to handle large reference datasets, each scRNA-seq dataset is randomly partitioned into $\lceil n / n_0\rceil$ equal-sized sub-reference datasets, where *n* is the number of cells in the scRNA-seq dataset and $\lceil \cdot \rceil$ is the ceiling function. By default, $n_0$ is set to 8000. The set of sub-reference datasets is represented as $\left\{ X^{\left( r\right) } \right\} _{r=1}^R$, where $X^{\left( r\right) }$ is an $n_r \times p$ gene expression matrix of the *r*-th sub-reference with rows representing cells and columns representing genes, *R* is the number of sub-reference datasets, $n_r$ is the number of cells in the *r*-th sub-reference, and *p* is the number of genes shared by all references. The gene expression matrix of the query dataset, *Y*, has *m* cells and *q* genes. ENGEP generates multiple base predictions for the $p - g$ unmeasured genes in the spatial dataset by using different sub-reference datasets, where $g = p \bigcap q$ represents the set of genes shared by the reference and query datasets.

For *k*-NN regression, it is important to use a similarity metric to find the closest neighboring cells in the reference data for the query cells. However, the optimal similarity measure for these two types of datasets has not been clearly established [14]. To address this, ten different similarity measures are employed: Pearson correlation coefficient, Spearman correlation coefficient, cosine similarity, Manhattan distance, Canberra distance, Euclidean distance, $\rho _{p}$ proportionality measure, $\phi _{s}$ proportionality measure, weighted rank correlation, and Jaccard index. For consistency, distance measures (such as Manhattan distance, Canberra distance, Euclidean distance, and $\phi _{s}$ proportionality measure) are transformed into similarity scores using $s = 1/(1+d)$, where a higher value indicates a greater similarity across all similarity measures. We denote the set of similarity measures used as $\mathbb {T}$.

The prediction results can vary with the chosen value of *k*. Determining the optimal value of *k* is still a challenge. To address this issue, we use multiple values of *k* (i.e., $\mathbb {K} = {20, 30, 40, 50}$) to generate multiple base predictions, making the method more flexible and providing a variety of base results. For correlation measures that can have negative values (e.g., Pearson correlation coefficient, Spearman correlation coefficient, cosine similarity, and weighted rank correlation), if the similarity score of the *k* nearest neighbors is negative, it is set to 0.

Mathematically, each sub-reference $X^{\left( r\right) }$ is split into two matrices: $X^{\left( r,1\right) }$, an $n_r \times g$ matrix representing the expression levels of *g* genes shared by the reference and query, and $X^{\left( r,2\right) }$, an $n_r \times \left( p-g\right)$ matrix representing the expression levels of $p-g$ genes unique to the sub-reference. Additionally, $Y^{\left( 1\right) }$ is denoted as the $m\times g$ submatrix of *Y* that corresponds to the expression levels of *g* genes shared by the sub-reference and query. Given a sub-reference *r* ($r=1,2,\ldots ,R$), a similarity measure *t* ($t \in \mathbb {T}$), and a value of *k* ($k \in \mathbb {K}$), the expression levels of gene $\ell \in \left( p-g\right)$ in query cell *i* are predicted as follows. First, based on $X^{\left( r,1\right) }$ and $Y^{\left( 1\right) }$, the *k* nearest neighbors (denoted as $N_{rtk}\left( i\right)$) from the $n_r$ cells in sub-reference *r* are identified, using similarity measure *t*. Then, the similarity between spatial cell *i* and its nearest neighbors $j \in N_{rtk}\left( i\right)$ is calculated, denoted as $w_{ij}^{\left( rtk\right) }$. The predicted expression of gene $\ell$ in query cell *i* (denoted as $\hat{Y}_{i \ell }^{\left( 2,rtk\right) }$) is then calculated as a weighted average of the nearest neighbor cells in sub-reference *r*,

1 $$\begin{aligned} \hat{Y}_{i \ell }^{\left( 2,rtk\right) } = \frac{\sum _{j \in N_{rtk}\left( i\right) } w_{ij}^{\left( rtk\right) } X^{\left( r,2\right) }_{j \ell }}{\sum _{j \in N_{rtk}\left( i\right) } w_{ij}^{\left( rtk\right) } }. \end{aligned}$$

In doing so, $R|\mathbb {T}||\mathbb {K}|$ base results for each cell *i* and gene $\ell$ are obtained using different combinations of *r*, *t* and *k*, where $|\cdot |$ is the cardinality of a set.

#### Step 2: Combining base results

After obtaining the base prediction results, we develop a weighted average ensemble method to produce a consensus prediction. To account for differences in the quality of the references and batch effects between the reference and query datasets, we propose a metric to quantify the predictive power of each sub-reference. The base predictions are then integrated by taking into account the estimated predictive power of each sub-reference.

Intuitively, a sub-reference that can better predict the common genes shared between the reference and the query has a stronger predictive power for genes that have not been spatially measured. Similar to Eq. (1), we first use *k*-NN regression to make predictions for common genes $\ell ' \in g$:

2 $$\begin{aligned} \hat{Y}_{i \ell '}^{\left( 1,rtk\right) } = \frac{\sum _{j \in N_{rtk}\left( i\right) } w_{ij}^{\left( rtk\right) } X^{\left( r,1\right) }_{j \ell '}}{\sum _{j \in N_{rtk}\left( i\right) } w_{ij}^{\left( rtk\right) } }. \end{aligned}$$

Then, the results from the base predictions are combined across different similarity measures and values of *k* to obtain a consensus prediction for each sub-reference:

3 $$\begin{aligned} \hat{Y}_{i \ell '}^{\left( 1,r\right) } = \frac{\sum _{t \in \mathbb {T}} \sum _{k \in \mathbb {K}} \hat{Y}_{i \ell '}^{\left( 1,rtk\right) }}{|\mathbb {T}||\mathbb {K}|}. \end{aligned}$$

The square of the Pearson correlation coefficient between the prediction expression matrix $\hat{Y}^{\left( 1,r\right) }$ and the observed expression matrix $Y^{\left( 1\right) }$ is used to estimate the predictive power of each sub-reference *r*: $s_r = cor^2 \left( \hat{Y}^{\left( 1,r\right) }, Y^{\left( 1\right) }\right)$. Finally, we use a linear map to convert the predictive power $s_r$ to a weight $\omega _r$, with a range of values between 0.1 and 0.9.

To take into account the predictive power of each sub-reference, we use the following weighted average ensemble method to obtain the consensus prediction result based on the $R|\mathbb {T}||\mathbb {K}|$ base prediction results:

4 $$\begin{aligned} \hat{Y}_{i \ell }^{\left( 2,r\right) } = \frac{ \sum _{r = 1}^R \sum _{t \in \mathbb {T}} \sum _{k \in \mathbb {K}} \omega _r \hat{Y}_{i \ell '}^{\left( 2,rtk\right) }}{|\mathbb {T}||\mathbb {K}| \sum _{r = 1}^R \omega _r }. \end{aligned}$$

Note that here we treat each similarity measure and number of neighbors equally based on the assumption that predictions produced using different similarity measures and number of neighbors are comparable. Therefore, the weight assigned to each base prediction is only related to the sub-reference, not to the similarity measure or the value of *k*.

### Datasets

We conduct experiments using three spatial transcriptomics datasets generated by different technologies: MERFISH, osmFISH, and STARmap. To analyze each spatial dataset, we collect multiple scRNA-seq reference datasets that profile the same or similar tissue as the spatial dataset (see Additional file 2: Table S1).

The MERFISH dataset is obtained from the Brain Image Library [19] and is measured from MOp (mouse brain primary motor cortex). We use scRNA-seq reference datasets provided by the BRAIN Initiative Cell Census Network [20] (BICCN) which profile the same region as the MERFISH dataset, including seven single-cell or single-nucleus transcriptomics datasets (scRNA-seq and snRNA-seq using 10x v2, v3 and SMART-Seq v4).

The osmFISH dataset [6] is measured from SMSc (mouse brain somatosensory cortex), and we use the following three scRNA-seq datasets as references. The Zeisel [28] dataset is measured by the same lab as the osmFISH dataset. The AllenSSp [29] dataset is more deeply sequenced than the Zeisel dataset and is measured from the same region as the osmFISH dataset. The AllenVISp [30] dataset is measured from a different region, VISc (mouse brain visual cortex), which is similar as SMSc.

The STARmap dataset [37] is measured from VISc. Its reference datasets are the same as those for the osmFISH dataset, as they are all measured from the same or similar mouse brain region.

### Data preprocessing

For the spatial transcriptomics datasets, we obtain the MERFISH datasets by selecting slices such as “$mouse2\_slice300$,” “$mouse2\_slice50$,” “$mouse1\_slice31$,” and “$mouse1\_slice313$.” Considering that there are four of the 258 genes measured by MERFISH showing poor staining [19], we use the rest 254 genes in this study. For the osmFISH dataset, we only use cells from cortical regions, and no genes are filtered from the osmFISH dataset. We use the smaller 1020-gene replicate (containing 973 cells) as the STARmap dataset, as it has lower gene sparsity compared to the larger one (containing 1549 cells). We filter out genes with sparsity higher than 0.7 and use the remaining 342 genes to train the algorithm. No cells are filtered from the STARmap dataset.

For scRNA-seq datasets, cells in the seven BICCN references datasets are filtered based on the quality-control files provided. For the Zeisel dataset, we use cells from the somatosensory cortex. For the AllenSSp and AllenVISp datasets, we filter out low quality cells according to metadata information. No filtration is applied on genes.

We use the Seurat R package to preprocess both the scRNA-seq and spatial datasets. We begin by selecting the genes that are common to both datasets. Then, we apply the NormalizeData function in the Seurat R package to normalize both the scRNA-seq and spatial datasets on these common genes.

### Cross-validation on spatially measured genes

We evaluate the performance of different methods on spatially measured genes through cross-validation. We randomly divide a set of *g* genes, shared by both the spatial and scRNA-seq datasets, into *N* equal-sized subsets. In each iteration, we leave out one of these subsets for evaluation and use the remaining $N-1$ subsets as common genes to predict the expression of the left-out set of genes. This process is repeated *N* times, each time with a different set of genes left out, so that we obtain predictions for all *g* genes. For the MERFISH and STARmap datasets, which have more shared genes, we set $N=5$ and perform a fivefold cross-validation. For the osmFISH dataset, which has fewer shared genes, we set *N* as the number of shared genes and conduct a leave-one-out cross-validation experiment.

We assess the performance by computing three metrics: Pearson correlation coefficient (PCC), Spearman correlation coefficient (SCC), and root mean square error (RMSE) between the measured and predicted expression for each gene. Better performance is indicated by higher PCC and SCC values as well as a lower RMSE value. Furthermore, we calculate an accuracy ratio (AR) to facilitate a clearer comparison between ENGEP and a compared method. The AR is determined as the ratio of the number of genes for which ENGEP provides better predictions to the number of genes for which ENGEP performs worse than the compared method. An AR value greater than 1 indicates that ENGEP performs better than the compared method. In addition to the quantitative metrics, we also conduct a visual comparison of the measured and predicted expression patterns. Due to technical noise, the measured expression levels of some genes may not be reliable. Therefore, a mismatch between the measured and predicted gene expression patterns does not necessarily indicate poor performance. Thus, we also compare the predicted patterns with those obtained from in situ hybridization (ISH) images in the Allen Brain Atlas or with patterns obtained using other high-sensitivity spatial transcriptomics technologies.

### Prediction of gene expression in unmeasured genes

We train the model using all genes shared by the reference and query datasets and predict the expression levels of the genes that are exclusive to the reference datasets. To improve the sensitivity of the analysis, we focus on the union of 2000 highly variable genes in each reference dataset and predict their expression levels. For the MERFISH, osmFISH, and STARmap datasets, we predict expression levels for 5234, 3433, and 2810 unmeasured genes, respectively.

### Identification of known spatial patterns from measured gene expression

We identify known spatial patterns through a graph-based clustering of spatially measured genes. Initially, we select spatially variable genes by employing Moran’s *I* with a stringent threshold, requiring an adjusted *P*-value of less than 0.05. To ensure robust results, we also exclude genes expressed in fewer than 5% of the cells. Subsequently, we calculate a Spatial Cross-Correlation Index (SCI) [48] for all remaining gene pairs. It is worth noting that our approach differs from the original MERINGUE approach [48], which does not consider self-loops when constructing the cell adjacency relationship network. In our implementation, we adapt the cell adjacency relationship matrix by normalizing it so that the total weight shared among neighboring cells in each row or column sums up to 0.5, while the diagonal element is set to 0.5. This adjustment allows us to assign higher SCI scores to pairs of genes exhibiting similar expression patterns across the same cells. We then construct a weighted gene network using the computed SCI scores among genes. We retain elements in the similarity matrix that surpass a certain threshold (at the 10th percentile) and replace the rest with zeros. Finally, we employ Louvain clustering on the constructed gene network, with the default resolution parameter set to 1. This partitions the genes into distinct expression patterns, and the average expression of genes within each pattern serves as the representative expression profile of a known pattern. Note that in the MERFISH and STARmap datasets, which encompass a broader set of measured genes, we employ a filter to remove identified patterns composed of just a single gene.

### Identification of novel spatial patterns from predicted gene expression

For spatially unmeasured genes predicted by our method, we also employ Moran’s I to filter out genes that do not exhibit spatial patterns and exclude genes expressed in fewer than 5% of the cells. Among the remaining genes, we identify the closest known spatial pattern by calculating the SCI scores between their predicted gene expressions and the expression profiles of all known patterns. The highest SCI score is regarded as the likelihood score, which indicates the degree of similarity between an unmeasured gene and a known pattern. Subsequently, we utilize Hartigan’s dip test to analyze the distribution of likelihood scores across all unmeasured genes to detect any potential novel patterns. The presence of a multimodality structure in the distribution of likelihood scores indicates the existence of novel patterns, while the absence of such a structure suggests that no novel patterns are present.

If novel patterns are present, we classify unmeasured genes into three distinct groups: those that exhibit a strong association with known patterns, those that show a weak association with known patterns, and those that are entirely different from the known patterns. Genes showing a strong association with known patterns are categorized within those established patterns. Conversely, genes that are distinctly different from the known patterns are considered part of novel patterns not previously observed in measured genes. Initially, we employ a kernel density estimation method to estimate the density function of the probability distribution for likelihood scores. This is achieved by using the density R function with default parameters. Subsequently, we identify the two high peaks within this estimated density function and and determine the likelihood score at the low peak between the two high peaks, denoted as *s*. Genes with likelihood scores greater than $s+0.05$ are classified as members of known patterns. Conversely, genes with likelihood scores lower than $s-0.05$ are designated as belonging to novel patterns. The remaining genes are considered weakly associated with known patterns. It is crucial to emphasize that our classification method does not rely on a mere threshold to assign unmeasured genes to either known or unknown pattern categories. Instead, it categorizes them into three distinct groups, enabling a clear differentiation between genes associated with novel patterns and those linked to known patterns, leading to the discovery of valuable insights into entirely new realms of biology.

For genes associated with known patterns, we straightforwardly assign them to the most closely matching established patterns. However, when dealing with genes linked to novel patterns, we follow a meticulous three-step process to unveil these novel spatial patterns. First, we apply a filter to exclude genes with lower biological variability. Specifically, we eliminate the lowest $10\%$ of genes based on their mean expression levels. Subsequently, we further refine this set by removing the lowest $10\%$ of genes with the least variability, employing the Seurat R package (FindVariableFeatures function). Next, akin to the procedure for identifying known spatial patterns from the measured genes, we utilize a graph-based clustering method to categorize the remaining genes into different clusters. Notably, we opt for a lower resolution parameter of 0.6 to prevent the formation of numerous small clusters, given the larger number of unmeasured genes in comparison to the measured ones. Finally, to ensure the biological relevance of our findings, we employ a stringent filtering process. Initially, we filter out clusters based on a three-times standard deviation rule. We calculate the mean similarity score (SCI) within each gene cluster and denote it as *Z*. Clusters are retained only if *Z* surpasses the mean by three times the standard deviation of SCI scores. Subsequently, we refine the retained clusters by iteratively eliminating genes with fewer than ten connections within their respective sub-gene networks. These refined clusters, characterized by their average gene expression, are considered as novel patterns.

Following the identification of novel spatial patterns, we conduct a multifaceted analysis to explore their biological significance. We begin by selecting representative genes from each pattern and assess their expression by comparing them to the corresponding ISH images in the Allen Brain Atlas. Next, we delve deeper into the predicted expression of these novel spatial patterns, investigating their potential associations with specific cell types within the tissue, as outlined in Additional file 1: Section S3.3. This analysis aims to establish links with distinct cell types or states, shedding light on the patterns’ biological relevance. Finally, we employ Gene Ontology enrichment analysis using the clusterProfiler R package to unveil the biological processes associated with these novel spatial patterns. Notably, for this analysis, the background gene list comprises the union of genes included in the reference datasets.

### ENGEP for single reference datasets

Despite being designed for multiple reference datasets, ENGEP can still be applied in situations where only a single reference dataset is available. In these cases, ENGEP generates base predictions by utilizing different similarity measures and different values of *k*. The final ensemble result is obtained by taking the average of these base predictions.

### Parameter settings for ENGEP

ENGEP does not require meticulously selected parameters, making it robust and user-friendly. However, there are still some parameters to consider: those related to pre-processing steps, the maximum number $n_0$ of cells in each sub-reference dataset when partitioning extensive reference datasets, and the array of *k* values used in KNN regression.

Our method does not require the use of specialized data pre-processing techniques. After filtering out cells and genes based on data quality control, we normalize the data using the Seurat R package. Within the function, we utilize the “LogNormalize” method as the “normalization.method” parameter, while keeping other parameters at their default values. The parameter $n_0$ determines the maximum number of cells in each sub-reference dataset, which can affect computational time and memory usage. We default to setting $n_0$ to 8000.

The selection of *k* values used in KNN regression may significantly influences performance and involves a trade-off between bias and variance. Lower *k* values result in reduced bias but increased variance, whereas higher *k* values yield lower variance but elevated bias. To strike an optimal balance between bias and variance, we adopt an ensemble learning approach that utilizes multiple *k* values, rather than a single value. We execute ENGEP with a range of *k* values (specifically, $\mathbb {K} = \{20, 30, 40, 50\}$) as the default. For users with reference datasets containing a modest number of cells, utilizing smaller *k* values is recommended. Conversely, for those with more extensive reference datasets, increasing the *k* values is advisable.

### Benchmarked methods

We evaluate the performance of ENGEP in comparison to five state-of-the-art methods: Seurat [9], SpaGE [10], stPlus [11], Tangram Cell, and Tangram Cluster [12]. Tangram-cluster is a variation of Tangram that runs its mapping process at the cell cluster level. All of the methods are applied using their default parameters or the settings specified in their accompanying documentation. We follow the data processing procedures, such as normalization and scaling, as outlined in the source code of each method. In addition, considering that some benchmarked methods report memory errors when comparing performance on MERFISH with large reference integrated by seven references, we use the union of high variable genes of every reference instead of all genes as the input features to run the benchmarked methods.

### Supplementary Information

**Additional file 1.** Supplementary Figures S1-S22 and Supplementary texts.

**Additional file 2.** Supplementary Tables S1-S7.

**Additional file 3.** Review history.

## Data Availability

The datasets analyzed during the current study are all publicly available. Spatial transcriptomics datasets are as follows: MERFISH (https://doi.org/10.35077/g.21) [49], osmFISH (http://linnarssonlab.org/osmFISH/) [50], and STARmap (https://kangaroo-goby.squarespace.com/data) [51]. ScRNA-seq (snRNA-seq) datasets are as follows: seven datasets from BRAIN Initiative Cell Census Network (https://assets.nemoarchive.org/dat-ch1nqb7) [52], Zeisel (http://linnarssonlab.org/cortex/) [53], AllenSSp, and AllenVISp (https://portal.brain-map.org/atlases-and-data/rnaseq) [54]. Images from Allen Brain Atlas are available at http://mouse.brain-map.org/ [55]. ENGEP is available as the open-source R package ENGEP, with source code freely available at https://github.com/Zhangxf-ccnu/ENGEP [56] and corresponding documentation at https://github.com/Zhangxf-ccnu/ENGEP-examples [57]. Additionally, the source code used in the manuscript is also deposited in Zenodo with a DOI assignment (DOI: https://doi.org/10.5281/zenodo.8365572) [58]. ALL repositories are released under MIT license.

## References

[1] Nitzan M, Karaiskos N, Friedman N, Rajewsky N (2019). Gene expression cartography. Nature..

[2] Moffitt JR, Bambah-Mukku D, Eichhorn SW, Vaughn E, Shekhar K, Perez JD (2018). Molecular, spatial, and functional single-cell profiling of the hypothalamic preoptic region. Science..

[3] Ståhl PL, Salmén F, Vickovic S, Lundmark A, Navarro JF, Magnusson J (2016). Visualization and analysis of gene expression in tissue sections by spatial transcriptomics. Science..

[4] Stickels RR, Murray E, Kumar P, Li J, Marshall JL, Di Bella DJ (2021). Highly sensitive spatial transcriptomics at near-cellular resolution with Slide-seqV2. Nat Biotechnol..

[5] Eng C-HL, Lawson M, Zhu Q, Dries R, Koulena N, Takei Y (2019). Transcriptome-scale super-resolved imaging in tissues by RNA seqFISH+. Nature.

[6] Codeluppi S, Borm LE, Zeisel A, La Manno G, van Lunteren JA, Svensson CI (2018). Spatial organization of the somatosensory cortex revealed by osmFISH. Nat Methods..

[7] Zhuang X (2021). Spatially resolved single-cell genomics and transcriptomics by imaging. Nat Methods..

[8] Moses L, Pachter L (2022). Museum of spatial transcriptomics. Nat Methods..

[9] Stuart T, Butler A, Hoffman P, Hafemeister C, Papalexi E, Mauck WM (2019). Comprehensive integration of single-cell data. Cell..

[10] Abdelaal T, Mourragui S, Mahfouz A, Reinders MJT. SpaGE: Spatial gene enhancement using scRNA-seq. Nucleic Acids Res. 2020;48:e107–e. 10.1093/nar/gkaa740 PMC7544237 32955565

[11] Chen S, Zhang B, Chen X, Zhang X, Jiang R (2021). stPlus: a reference-based method for the accurate enhancement of spatial transcriptomics. Bioinformatics..

[12] Biancalani T, Scalia G, Buffoni L, Avasthi R, Lu Z, Sanger A (2021). Deep learning and alignment of spatially resolved single-cell transcriptomes with Tangram. Nat Methods..

[13] Tran HTN, Ang KS, Chevrier M, Zhang X, Lee NYS, Goh M (2020). A benchmark of batch-effect correction methods for single-cell RNA sequencing data. Genome Biol..

[14] Skinnider MA, Squair JW, Foster LJ (2019). Evaluating measures of association for single-cell transcriptomics. Nat Methods..

[15] Lein ES, Hawrylycz MJ, Ao N, Ayres M, Bensinger A, Bernard A (2007). Genome-wide atlas of gene expression in the adult mouse brain. Nature..

[16] Quinn TP, Richardson MF, Lovell D, Crowley TM (2017). propr: An R-package for identifying proportionally abundant features using compositional data analysis. Sci Rep..

[17] Chen KH, Boettiger AN, Moffitt JR, Wang S, Zhuang X (2015). Spatially resolved, highly multiplexed RNA profiling in single cells. Science..

[18] Lewis SM, Asselin-Labat M-L, Nguyen Q, Berthelet J, Tan X, Wimmer VC (2021). Spatial omics and multiplexed imaging to explore cancer biology. Nat Methods..

[19] Zhang M, Eichhorn SW, Zingg B, Yao Z, Cotter K, Zeng H (2021). Spatially resolved cell atlas of the mouse primary motor cortex by MERFISH. Nature..

[20] Yao Z, Liu H, Xie F, Fischer S, Adkins RS, Aldridge AI (2021). A transcriptomic and epigenomic cell atlas of the mouse primary motor cortex. Nature..

[21] Muñoz-Castañeda R, Zingg B, Matho KS, Chen X, Wang Q, Foster NN (2021). Cellular anatomy of the mouse primary motor cortex. Nature..

[22] Sherman DL, Brophy PJ (2005). Mechanisms of axon ensheathment and myelin growth. Nat Rev Neurosci..

[23] Tomassy GS, Dershowitz LB, Arlotta P (2016). Diversity matters: a revised guide to myelination. Trends Cell Biol..

[24] Molofsky AV, Krenick R, Ullian E, Tsai H-H, Deneen B, Richardson WD (2021). Astrocytes and disease: a neurodevelopmental perspective. Genes Dev..

[25] Dudley AC, Griffioen AW (2023). Pathological angiogenesis: mechanisms and therapeutic strategies. Angiogenesis..

[26] Bergers G, Song S (2005). The role of pericytes in blood-vessel formation and maintenance. Neuro Oncol..

[27] Waylen LN, Nim HT, Martelotto LG, Ramialison M (2020). From whole-mount to single-cell spatial assessment of gene expression in 3D. Commun Biol..

[28] Zeisel A, Muñoz-Manchado AB, Codeluppi S, Lönnerberg P, La Manno G, Juréus A (2015). Cell types in the mouse cortex and hippocampus revealed by single-cell RNA-seq. Science..

[29] Chatterjee S, Sullivan HA, MacLennan BJ, Xu R, Hou Y, Lavin TK (2018). Nontoxic, double-deletion-mutant rabies viral vectors for retrograde targeting of projection neurons. Nat Neurosci..

[30] Tasic B, Yao Z, Graybuck LT, Smith KA, Nguyen TN, Bertagnolli D (2018). Shared and distinct transcriptomic cell types across neocortical areas. Nature..

[31] Ferrere A, Vitalis T, Gingras H, Gaspar P, Cases O (2006). Expression of Cux-1 and Cux-2 in the developing somatosensory cortex of normal and barrel-defective mice. Anat Rec A Discov Mol Cell Evol Biol..

[32] Ferland RJ, Cherry TJ, Preware PO, Morrisey EE, Walsh CA (2003). Characterization of Foxp2 and Foxp1 mRNA and protein in the developing and mature brain. J Comp Neurol..

[33] John DC, Ben E, Amit K, Lynette CF, Jennifer LZ, Karen SC (2008). A transcriptome database for astrocytes, neurons, and oligodendrocytes: A new resource for understanding brain development and function. J Neurosci..

[34] McBain CJ, Fisahn A (2001). Interneurons unbound. Nat Rev Neurosci..

[35] Dalva MB, McClelland AC, Kayser MS (2007). Cell adhesion molecules: signalling functions at the synapse. Nat Rev Neurosci..

[36] Ren C, Peng K, Yang R, Liu W, Liu C, Komiyama T (2022). Global and subtype-specific modulation of cortical inhibitory neurons regulated by acetylcholine during motor learning. Nat Rev Neurosci..

[37] Wang X, Allen WE, Wright MA, Sylwestrak EL, Samusik N, Vesuna S (2018). Three-dimensional intact-tissue sequencing of single-cell transcriptional states. Science..

[38] Kippert A, Trajkovic K, Fitzner D, Opitz L, Simons M (2008). Identification of Tmem10/Opalin as a novel marker for oligodendrocytes using gene expression profiling. BMC Neurosci..

[39] Oishi K, Nakagawa N, Tachikawa K, Sasaki S, Aramaki M, Hirano S (2016). Identity of neocortical layer 4 neurons is specified through correct positioning into the cortex. eLife..

[40] Hanisch U-K, Kettenmann H (2007). Microglia: active sensor and versatile effector cells in the normal and pathologic brain. Nat Neurosci..

[41] Abbott NJ, Rönnbäck L, Hansson E (2006). Astrocyte-endothelial interactions at the blood-brain barrier. Nat Rev Neurosci..

[42] Munji RN, Soung AL, Weiner GA, Sohet F, Semple BD, Trivedi A (2019). Profiling the mouse brain endothelial transcriptome in health and disease models reveals a core blood-brain barrier dysfunction module. Nat Neurosci..

[43] Lilly B (2014). We have contact: endothelial cell-smooth muscle cell interactions. Physiology..

[44] Li B, Zhang W, Guo C, Xu H, Li L, Fang M (2022). Benchmarking spatial and single-cell transcriptomics integration methods for transcript distribution prediction and cell type deconvolution. Nat Methods..

[45] Cang Z, Nie Q (2020). Inferring spatial and signaling relationships between cells from single cell transcriptomic data. Nat Commun..

[46] Olsen TK, Baryawno N (2018). Introduction to single-cell RNA sequencing. Curr Protoc Mol Biol..

[47] Ziegenhain C, Vieth B, Parekh S, Reinius B, Guillaumet-Adkins A, Smets M (2017). Comparative analysis of single-Cell RNA Sequencing methods. Mol Cell..

[48] Miller BF, Bambah-Mukku D, Dulac C, Zhuang X, Fan J (2021). Characterizing spatial gene expression heterogeneity in spatially resolved single-cell transcriptomic data with nonuniform cellular densities. Genome Res..

[49] Zhuang X, Zhang M. Brain Image Library. 10.35077/g.21. Accessed 4 Dec 2021.

[50] Codeluppi S, Borm LE, Zeisel A, La Manno G, van Lunteren JA, Svensson CI, et al. osmFISH Dataset. http://linnarssonlab.org/osmFISH/. Accessed 21 Nov 2021.

[51] STARmap Resources. https://kangaroo-goby.squarespace.com/data. Accessed 16 Oct 2021.

[52] Yao Z, Liu H, Xie F, Fischer S, Booeshaghi AS, Adkins RS, et al. The Neuroscience Multi-omic Data Archive. https://assets.nemoarchive.org/dat-ch1nqb7. Accessed 23 Dec 2021.

[53] Zeisel A, Muñoz-Manchado AB, Codeluppi S, Lönnerberg P, La Manno G, Juréus A, et al. Zeisel dataset. http://linnarssonlab.org/cortex/. Accessed 26 Nov 2021.

[54] Allen Brain Map Knowledge Base. Cell types database: RNA-Seq data. http://linnarssonlab.org/cortex/. Accessed 28 Oct 2021.

[55] Allen Mouse Brain Atlas. http://mouse.brain-map.org/. Accessed 10 May 2022.

[56] Yang ST, Zhang XF. R package ENGEP. Github. https://github.com/Zhangxf-ccnu/ENGEP. Accessed 18 Apr 2023.

[57] Yang ST, Zhang XF. A tutorial of R package ENGEP. Github. https://github.com/Zhangxf-ccnu/ENGEP-examples. Accessed 18 Apr 2023.

[58] Yang ST, Zhang XF. R package ENGEP. Zenodo. 10.5281/zenodo.8365572. Accessed 21 Sep 2023.

