# Supplementary material for: ENGEP: advancing spatial transcriptomics with accurate unmeasured gene expression prediction
Source: Genome Biol. 2023 Dec 21;24:293. doi: 10.1186/s13059-023-03139-w (PMC10734203; doi:10.1186/s13059-023-03139-w)
Supplement: Supplementary file 1 — Additional file 1. Supplementary Figures S1-S22 and Supplementary texts. [file 13059_2023_3139_MOESM1_ESM.docx]

Supplementary Information

ENGEP: advancing spatial transcriptomics with accurate unmeasured gene expression prediction

[1 Index of Supplementary Tables 2](#_Toc146491059)

[2 Supplementary Figures 4](#_Toc146491060)

[3 Supplementary Texts 26](#_Toc146491061)

[3.1 Impact of the number of references on performance 26](#_Toc146491062)

[3.2 Analysis of similarity measures 27](#_Toc146491063)

[3.2.1 Performance evaluation of similarity measures 27](#_Toc146491064)

[3.2.2 Computational time of similarity measures 28](#_Toc146491065)

[3.3 Identification of cell types colocalized with spatial patterns 28](#_Toc146491066)

# 1 Index of Supplementary Tables

**Table S1. Summary of datasets used by ENGEP.**

We use three spatial datasets containing MERFISH, osmFISH, and STARmap. For each spatial dataset, we collect multiple scRNA-seq (snRNA-seq) datasets to apply ENGEP. Different datasets differ in the number of cells, the number of genes, and tissues. MOp: primary motor cortex; SMSc: Somatosensory cortex; VISc: Visual cortex.

**Table S2. The cumulative Pearson correlation of patterns and cell types in MERFISH.**

For each spatial pattern, we rank the cell types based on their correlations with the pattern's gene expression. Cell types marked with asterisks (*) indicate those that have been selected as co-located with this pattern. The cumulative correlation is provided as each cell type is iteratively added. To illustrate, let's consider known pattern 1 as an example: the first row displays the correlation between known pattern 1 and the L6 CT cell type, while the second row presents the correlation between this pattern and two cell types (L6CT and L5 IT), and so forth. Astro, astrocyte; CT, corticothalamic; Endo, endothelial; ET, extratelencephalically projecting; IT, intratelencephalically projecting; Micro, microglial cell; Oligo, oligodendrocyte; OPC, oligodendrocyte precursor cell; Peri, pericyte; PVM, perivascular macrophage; SMC, smooth muscle cell; VLMC, vascular leptomeningeal cell.

**Table S3. Genes and functions related to the novel patterns in MERFISH.**

Three novel patterns are identified in MERFISH, each comprising 202, 206, and 284 genes, respectively. Functional enrichment analysis of genes within each pattern revealed significant enrichments in 37, 333, and 560 biological processes (BP), respectively.

**Table S4. The cumulative Pearson correlation of patterns and cell types in osmFISH.**

For each spatial pattern, we rank the cell types based on their correlations with the pattern's gene expression. Cell types marked with asterisks (*) indicate those that have been selected as co-located with this pattern. The cumulative correlation is provided as each cell type is iteratively added. Take the known pattern 1 as an example, the first row shows the correlation between the known pattern 1 and the Pyramidal L2-3 cell type, while the second row shows the correlation between this pattern and two cell types (Pyramidal L2-3 and Pyramidal L2-3 L5), and so forth.

**Table S5. Genes and functions related to the novel patterns in osmFISH.**

Two novel patterns are identified in osmFISH, each comprising 202 and 286 genes. Functional enrichment analysis of genes within each pattern revealed significant enrichments in 17 and 35 biological processes (BP).

**Table S6. The cumulative Pearson correlation of patterns and cell types in STARmap.**

For each spatial pattern, we rank the cell types based on their correlations with the pattern's gene expression. Cell types marked with asterisks (*) indicate those that have been selected as co-located with this pattern. The cumulative correlation is provided as each cell type is iteratively added. Take the known pattern 1 as an example, the first row shows the correlation between the known pattern 1 and the eL4 cell type, while the second row shows the correlation between this pattern and two cell types (eL4 and eL2/3), and so forth. Astro, astrocytes; Oligo, oligodendrocytes; Smc, smooth muscle cells; Endo, endothelial cells; HPC, hippocampus; Other, other unclassified cells; Micro, microglia; inhibitory (VIP, Reln, SST, Lhx6, and NPY); excitatory (eL2/3, eL5–1, eL5–2, eL5–3, eL6–1, and eL6–2).

**Table S7. Genes and functions related to the novel patterns in STARmap.**

Three novel patterns are identified in STARmap, each comprising 61, 254, and 392 genes, respectively. Functional enrichment analysis of genes within each pattern revealed significant enrichments in 384, 230, and 486 biological processes (BP), respectively.

Supplementary Tables are available in Additional file 2: Supplementary Tables.

# 2 Supplementary Figures

**
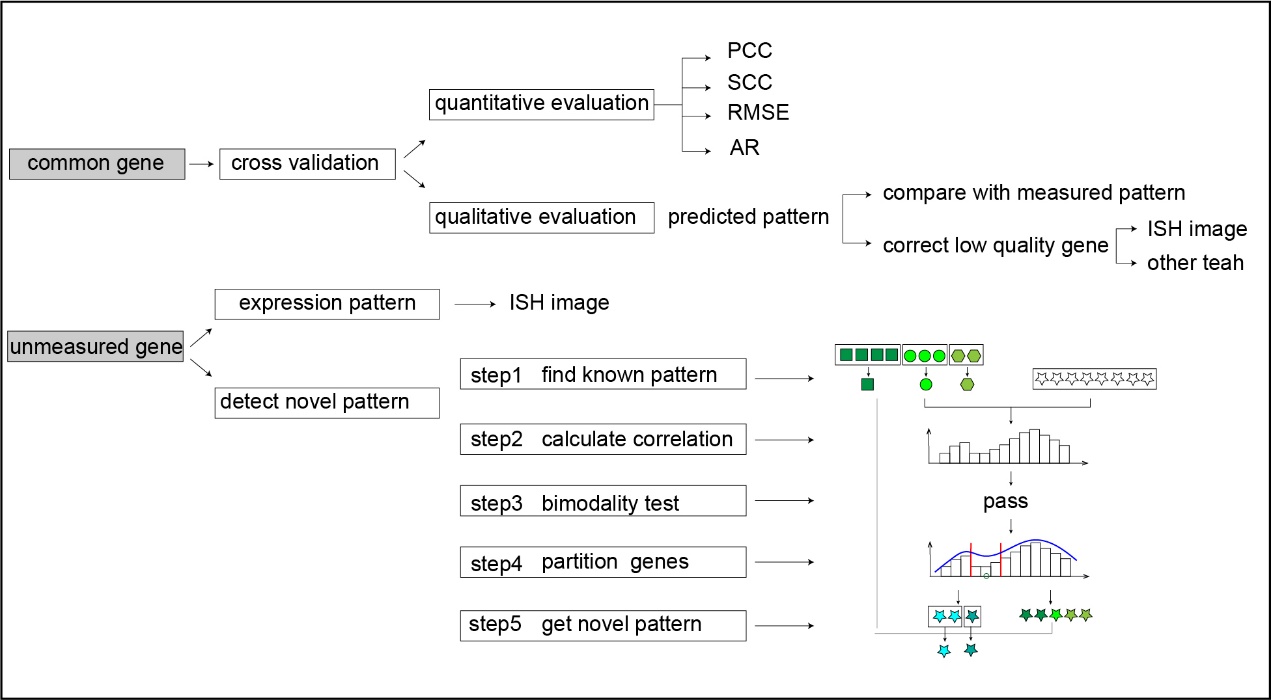
Fig. S1. Experiment procedure of ENGEP.** We test the ability of ENGEP from two aspects. First, we conduct cross-validation on genes shared by the query and reference datasets. We use four quantitative evaluations to compare the performance of ENGEP and benchmarked methods. Then we evaluate qualitatively in terms of predicted expression patterns by visually comparing them with the measured ones and assessing ENGEP’s ability to correct for low-quality genes. Second, we demonstrate the ability of ENGEP to predict spatially unmeasured genes. The predicted expression of these genes can be compared with ISH images from the Allen Brain Atlas. Employing spatial pattern analysis, we also identify previously uncharacterized spatial expression patterns in the predicted unmeasured genes.

**
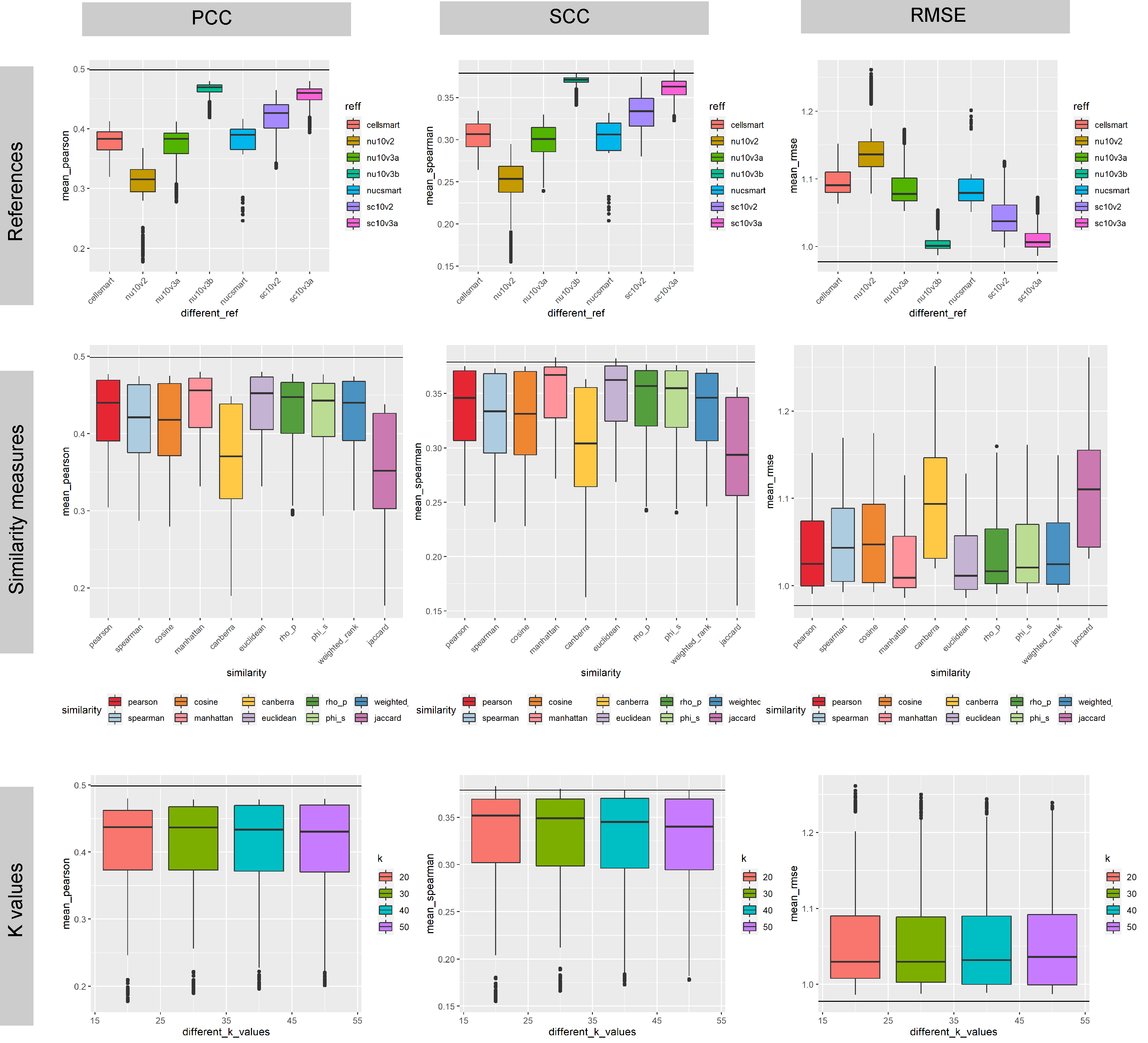
Fig. S2. Comparison of performance between the ensemble result and individual base predictions in MERFISH.** Each boxplot illustrates the disparity in performance when employing distinct references, similarity measures, and $k$ values. The columns signify the utilization of PCC, SCC, and RMSE metrics for assessment, while the rows present performance across diverse references, similarity measures, and $k$ values. The central black line represents the mean value of the evaluation scores for the ensemble outcome.


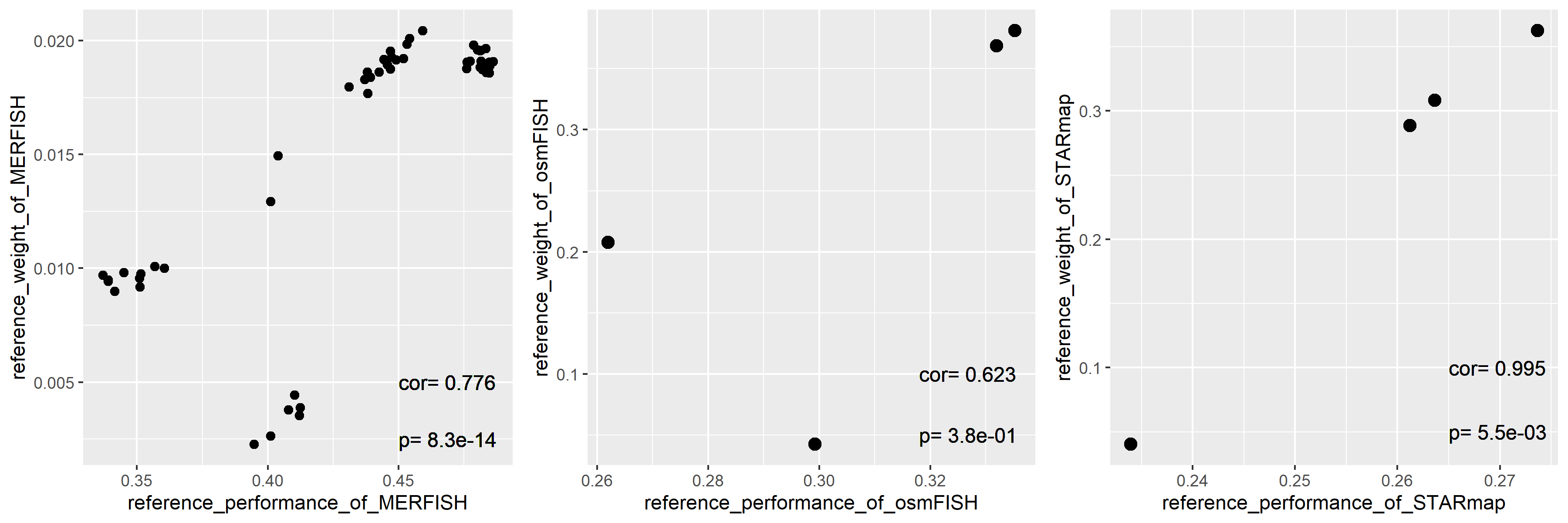


**Fig. S3. Relationship between weights and performance of base predictions in ENGEP**. This figure illustrates the correlation between the weights (y-axis) assigned by ENGEP to each base prediction and the corresponding performance (x-axis, mean PCC value) of the base predictions. In each plot, the lower right corner presents the Pearson correlation coefficient (cor) and p-value from the correlation test. The left plot represents the MERFISH result, whereas the middle and right plots depict the outcomes of osmFISH and STARmap, respectively.

**
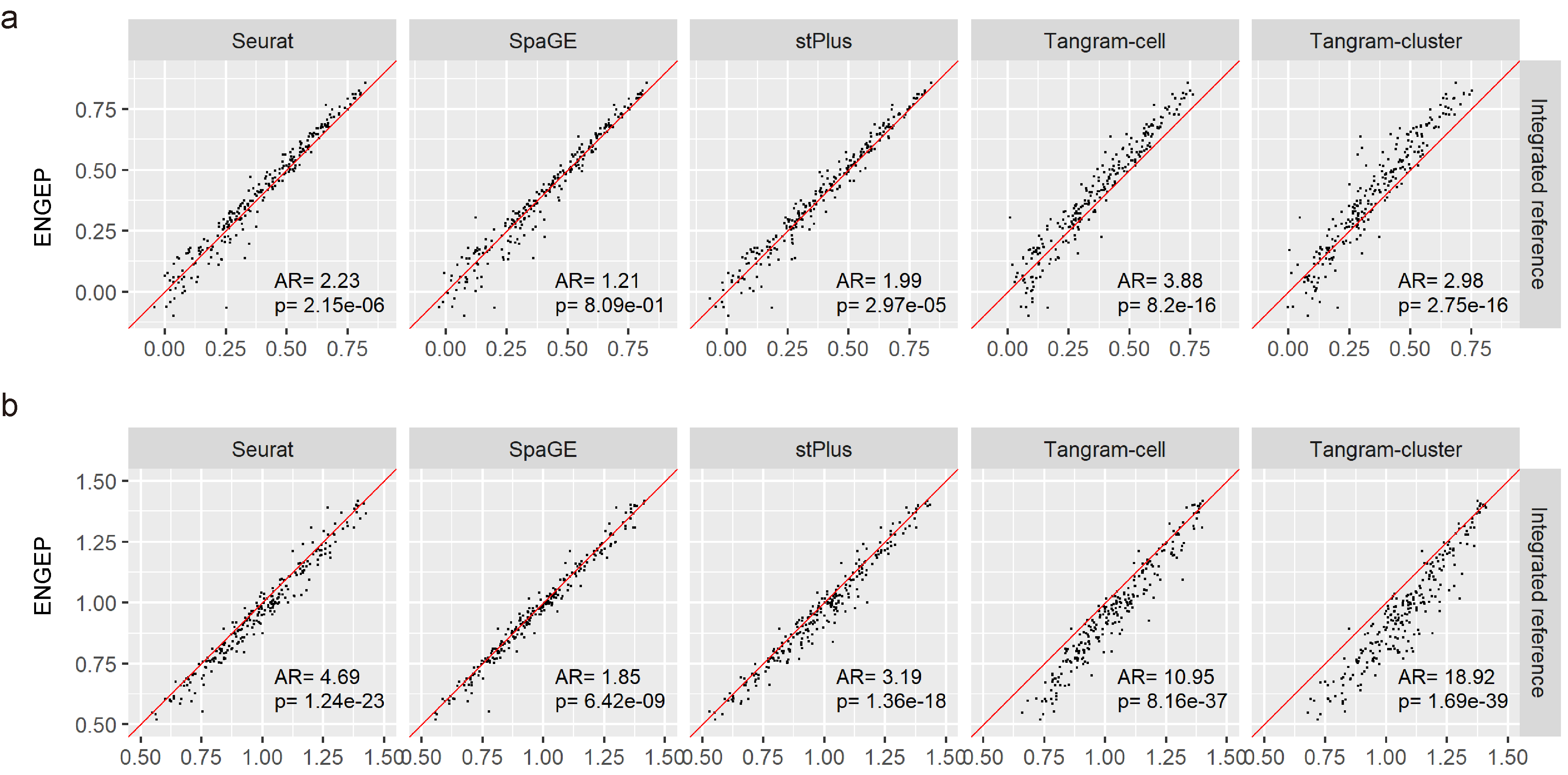
 Fig. S4. Performance comparison on predicting spatially measured genes for MERFISH via 5-fold cross-validation.** The comparison encompasses ENGEP and benchmarked methods, evaluated through SCC (a) and RMSE (b) metrics. The red line signifies the y=x reference line. The included P-value highlights significant disparities between ENGEP and alternative methods based on the Wilcoxon rank-sum test. The AR value quantifies the ratio of genes predicted with superior performance by ENGEP relative to other methods.


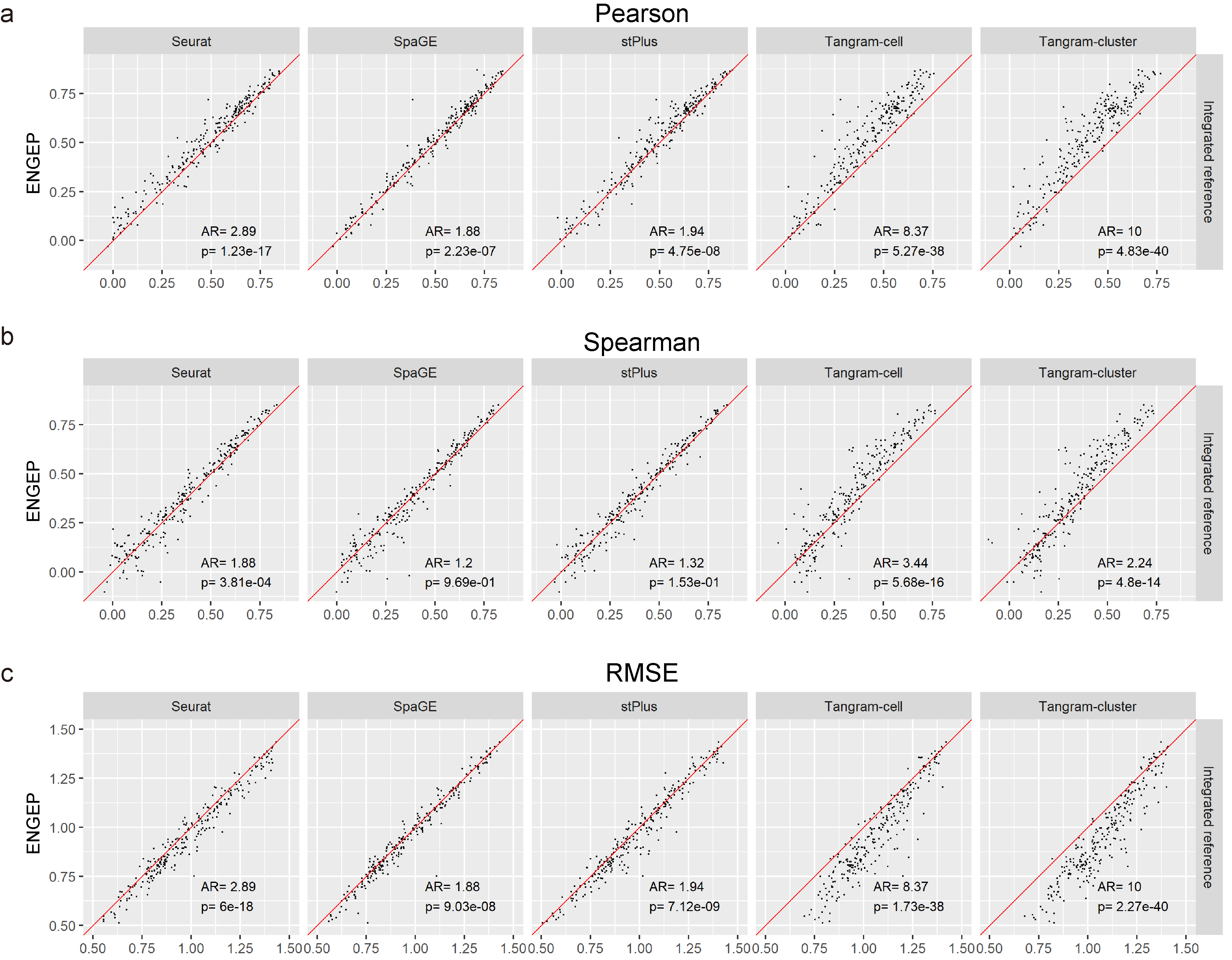


**Fig. S5. Performance comparison on predicting spatially measured genes for MERFISH slice “mouse2-slice50” via 5-fold cross-validation.** The comparison encompasses ENGEP and benchmarked methods, evaluated through PCC (a), SCC (b) and RMSE (c) metrics. The red line signifies the y=x reference line. The included P-value highlights significant disparities between ENGEP and alternative methods based on the Wilcoxon rank-sum test. The AR value quantifies the ratio of genes predicted with superior performance by ENGEP relative to other methods.

**
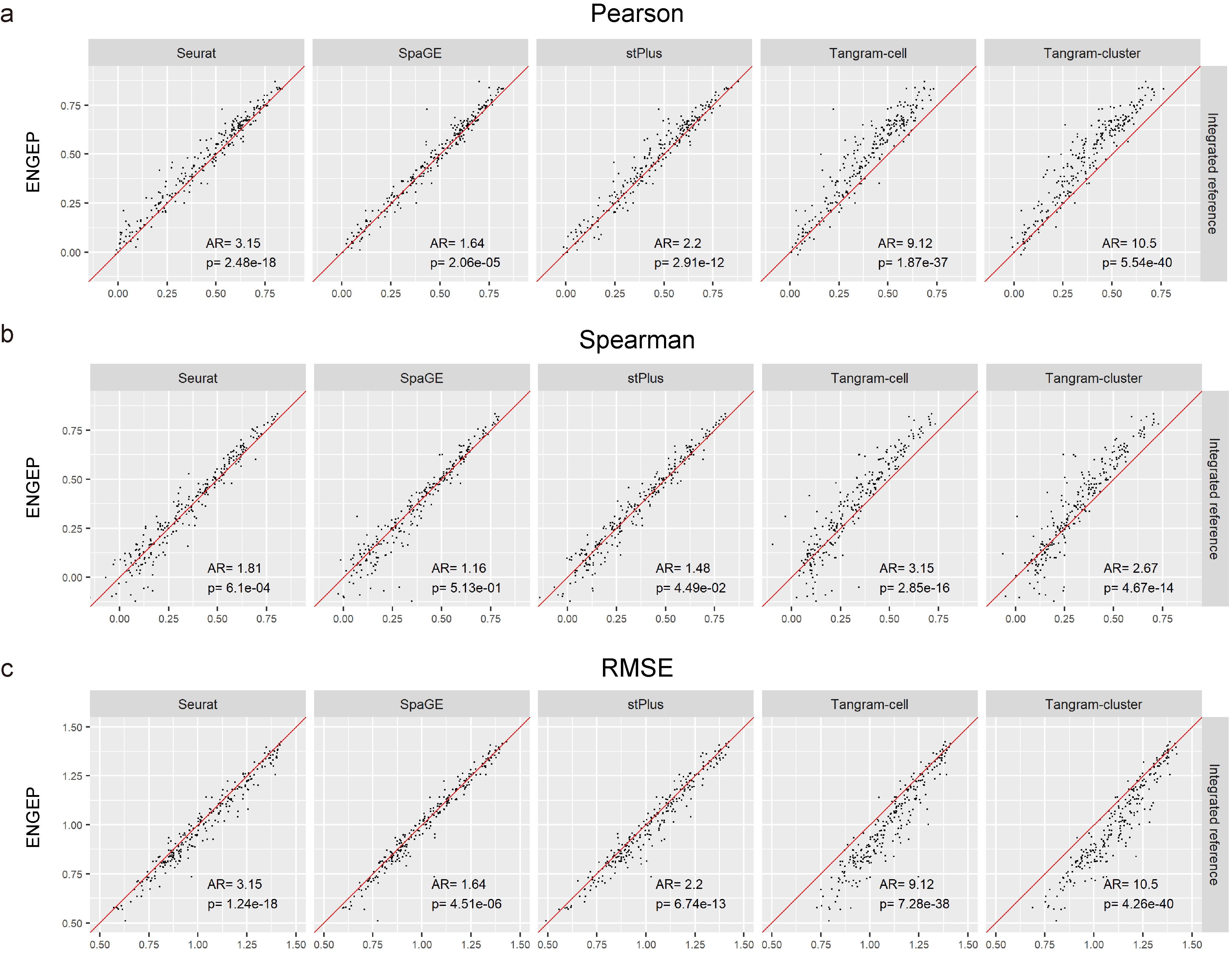
**

**Fig. S6. Performance comparison on predicting spatially measured genes for MERFISH slice “mouse1-slice31” via 5-fold cross-validation.** The comparison encompasses ENGEP and benchmarked methods, evaluated through PCC (a), SCC (b) and RMSE (c) metrics. The red line signifies the y=x reference line. The included P-value highlights significant disparities between ENGEP and alternative methods based on the Wilcoxon rank-sum test. The AR value quantifies the ratio of genes predicted with superior performance by ENGEP relative to other methods.

**
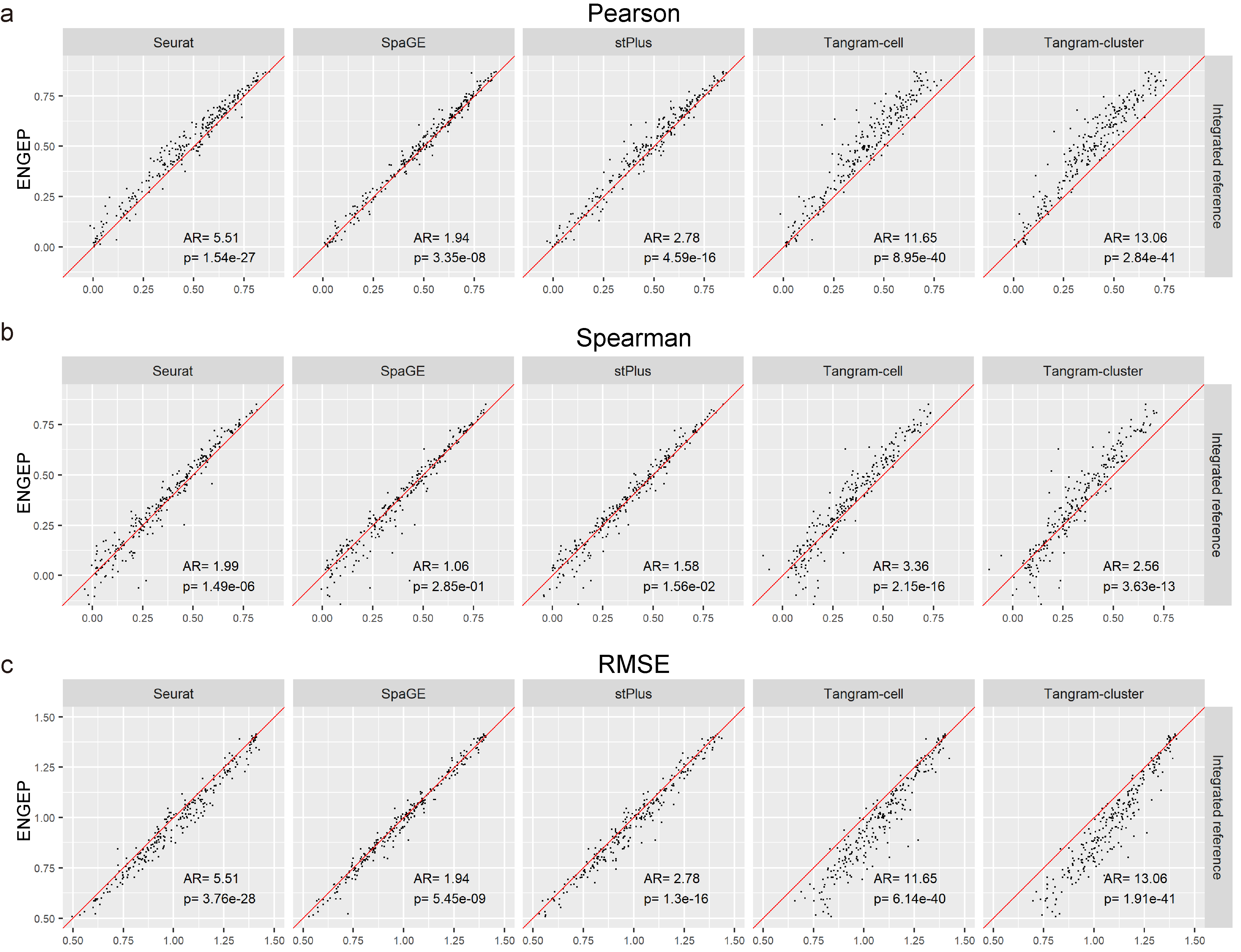
**

**Fig. S7. Performance comparison on predicting spatially measured genes for MERFISH slice “mouse1-slice313” via 5-fold cross-validation.** The comparison encompasses ENGEP and benchmarked methods, evaluated through PCC (a), SCC (b) and RMSE (c) metrics. The red line signifies the y=x reference line. The included P-value highlights significant disparities between ENGEP and alternative methods based on the Wilcoxon rank-sum test. The AR value quantifies the ratio of genes predicted with superior performance by ENGEP relative to other methods.


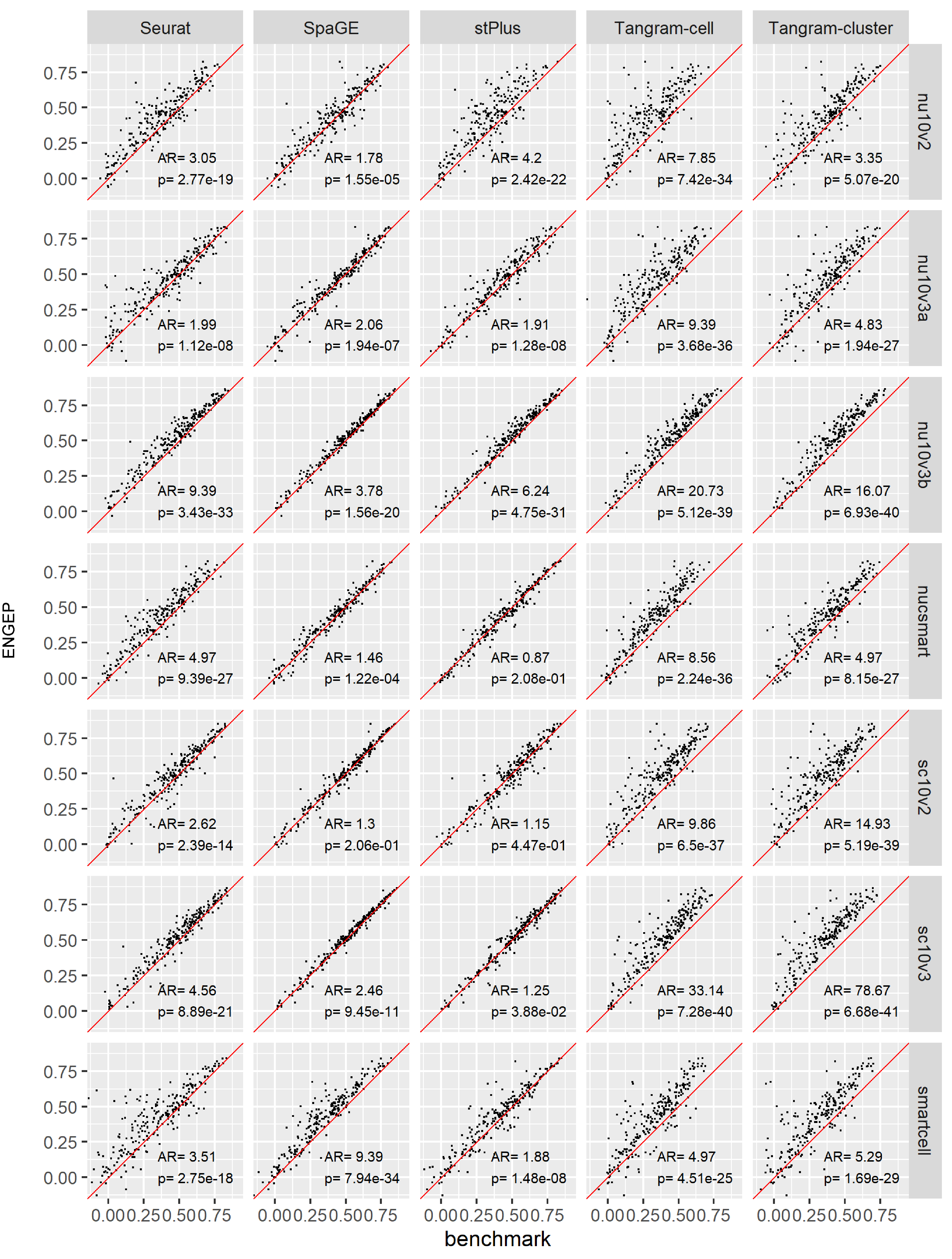


**Fig. S8. Performance comparison between ENGEP and other methods by using only one reference to predict spatially measured genes for MERFISH via 5-fold cross-validation.** From left to right, there are five columns representing five benchmarked methods, including Seurat, SpaGE, stPlus, Tangram-cell and Tangram-cluster. From top to bottom, there are seven rows, which represent seven different references, including nu10v2, nu10v3a, nu10v3b, smartnuc, sc10v2, sc10v3, and smartcell. We run ENGEP and the five compared methods seven times, each time using only one reference dataset.

**
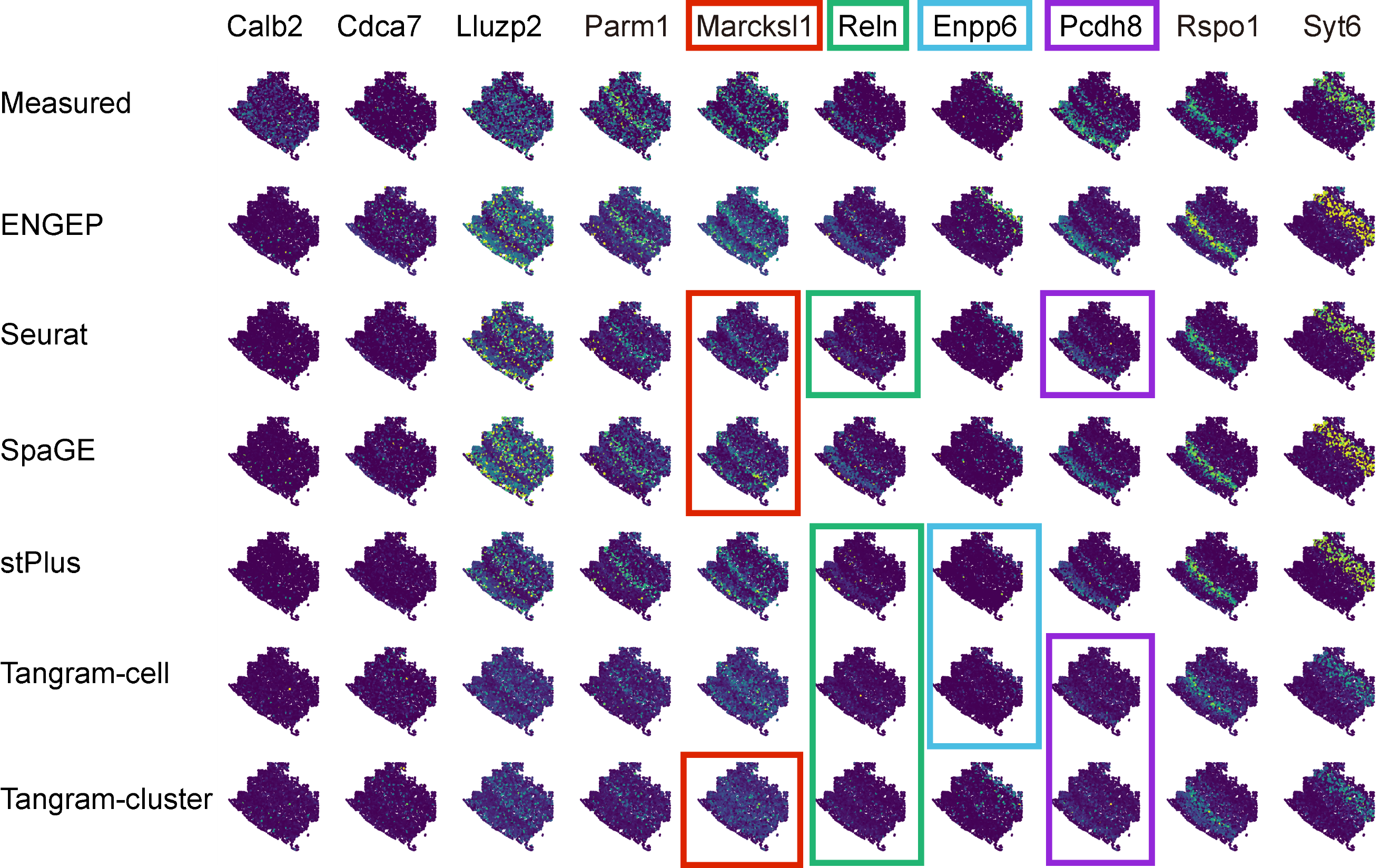
 Fig. S9. Expression patterns of measured genes in MERFISH predicted by different methods.** The expression patterns predicted by ENGEP are more consistent with the measured patterns than the benchmarked methods. To illustrate, Seurat, SpaGE, and Tangram-cell exhibit shortcomings in accurately predicting expression in the upper region of *Marcksl1*, primarily inhabited by oligodendrocyte cells. Similarly, when assessing the layer structure of *Reln*, characterized by its prominent expression in the L2/3 IT layer, Seurat, stPlus, and Tangram do not effectively capture this pattern. In case of *Enpp6*, which displays a high level of expression in oligodendrocyte cells, both Stplus and Tangram-cell yield patterns with reduced contrast. Lastly, for *Pcdh8* with a high expression in the L5 ET and L2/3 IT layers, Seurat and Tangram produce patterns with lower contrast in the L5 ET layer and struggle to reproduce patterns in the L2/3 IT layer.


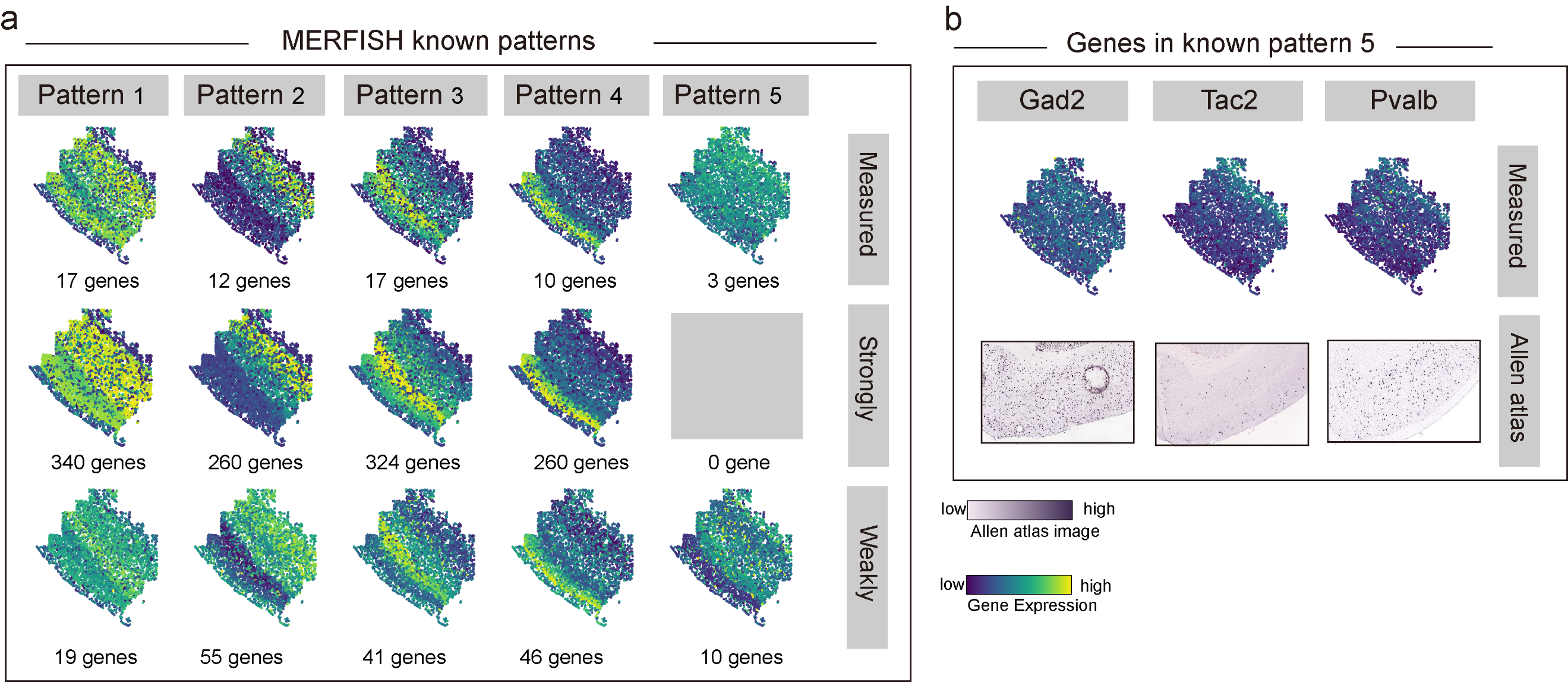


**Fig. S10. Alignment of unmeasured genes to the five known patterns in MERFISH.** a. The top row displays the expression levels of the five known patterns along with the number of genes associated with each pattern. The middle row shows the expression of known patterns, averaged using strongly associated genes. Notably, there are no genes strongly associated with known pattern 5. The bottom row presents the expression of known patterns, averaged using weakly associated genes. b. Depicted are the measured expression levels and corresponding ISH images of three constituent genes within known pattern 5. The three constituent genes do not closely resemble the corresponding ISH images, implying the presence of latent noise in the measured values. Known pattern 5 includes significantly fewer genes than the other four known patterns, and these three constituent genes exhibit limited similarity to each other, suggesting potentially lower biological significance. The noise in the expression profiles of these genes and the smaller number of genes it includes may provide an explanation for the absence of strongly associated predicted unmeasured genes with known pattern 5.

**
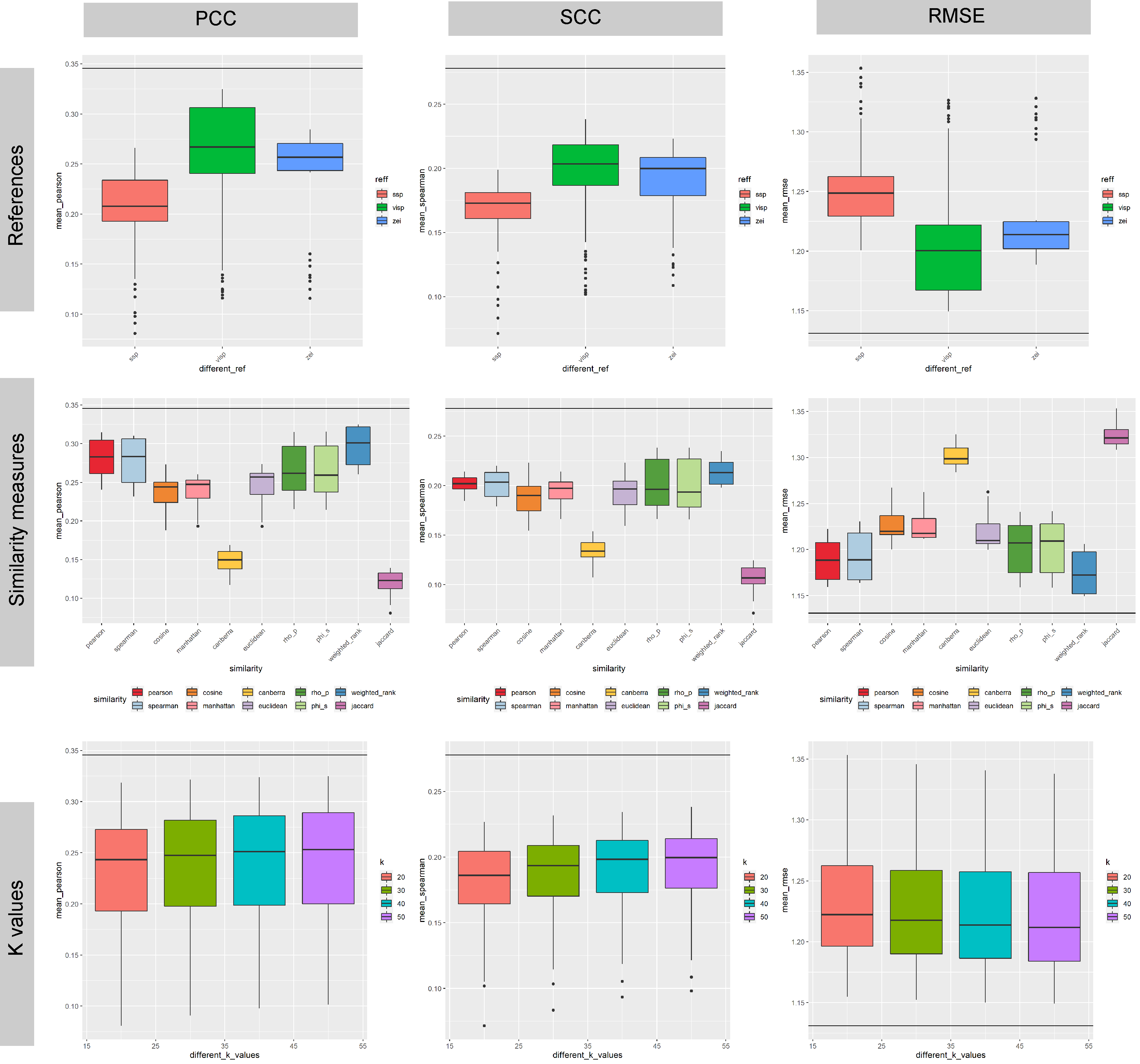
**

**Fig. S11. Comparison of performance between the ensemble result and individual base predictions in osmFISH.** Each boxplot illustrates the disparity in performance when employing distinct references, similarity measures, and $k$ values. The columns signify the utilization of PCC, SCC, and RMSE metrics for assessment, while the rows present performance across diverse references, similarity measures, and $k$ values. The central black line represents the mean value of the evaluation scores for the ensemble outcome.


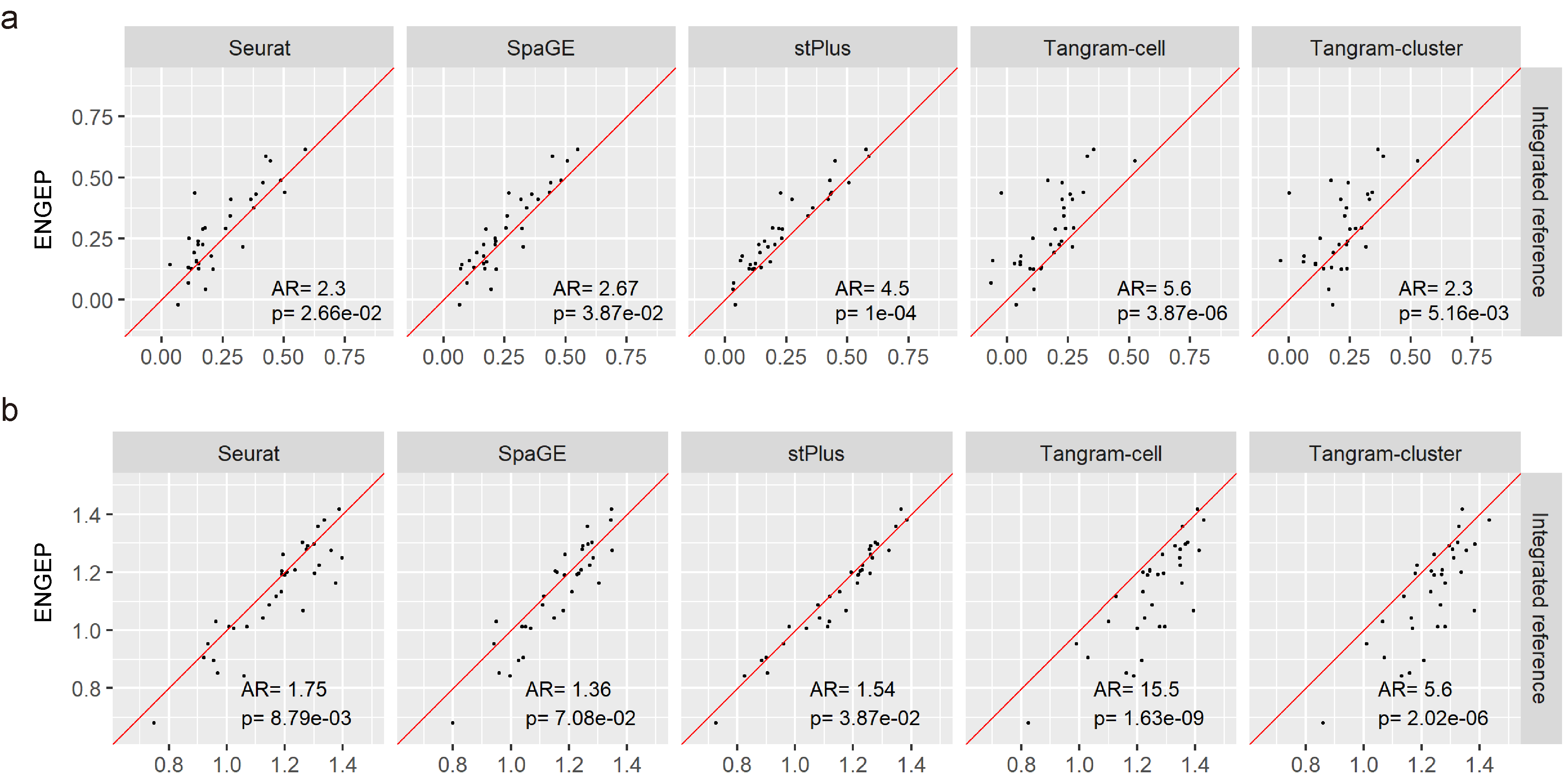


**Fig. S12. Performance comparison on predicting spatially measured genes for osmFISH via leave-one-gene-out cross-validation.** The comparison encompasses ENGEP and benchmarked methods, evaluated through SCC (a) and RMSE (b) metrics. The red line signifies the y=x reference line. The included P-value highlights significant disparities between ENGEP and alternative methods based on the Wilcoxon rank-sum test. The AR value quantifies the ratio of genes predicted with superior performance by ENGEP relative to other methods.


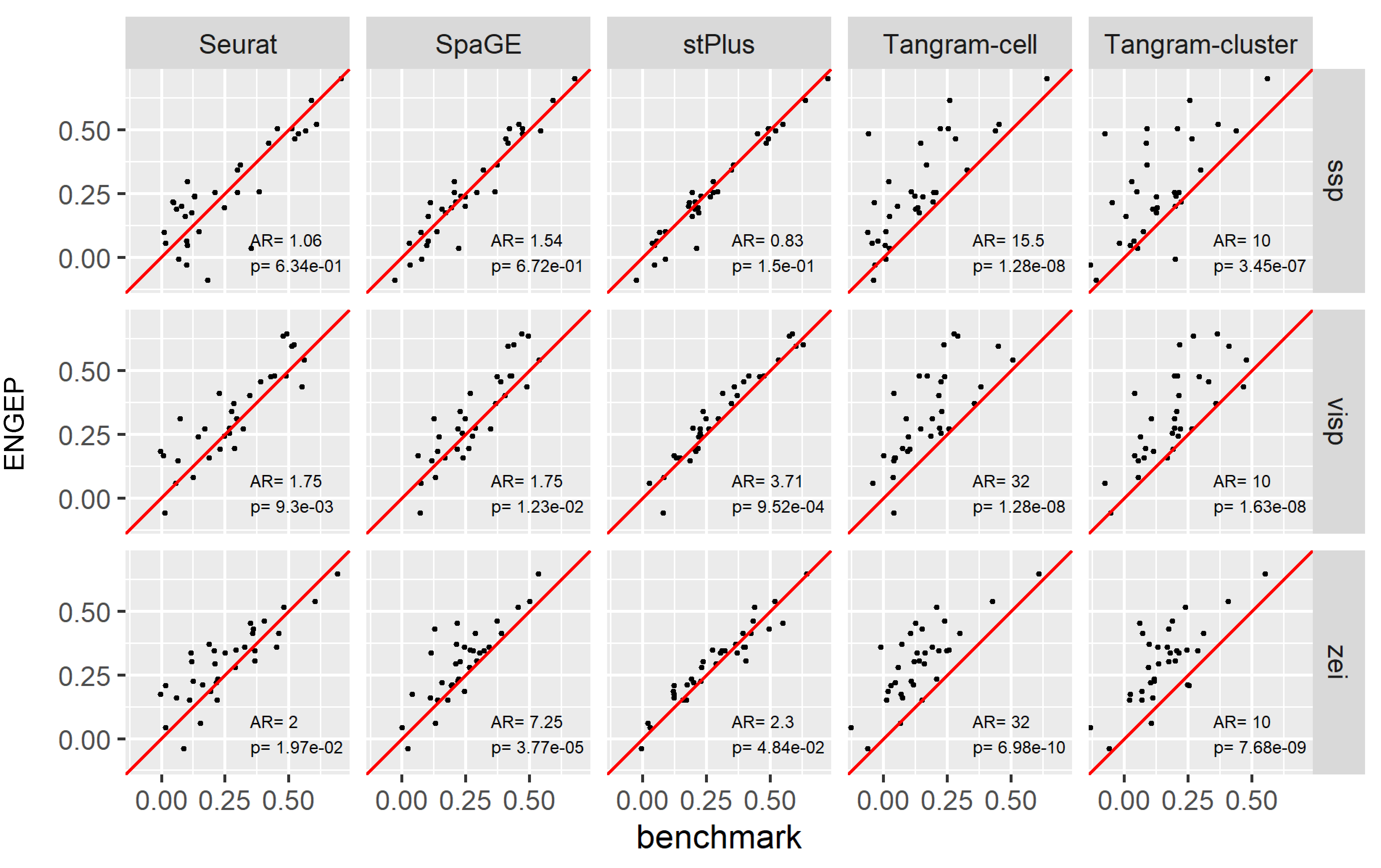


**Fig. S13. Performance comparison between ENGEP and other methods by using only one reference to predict spatially measured genes for osmFISH.** From left to right, there are 5 columns representing 5 benchmarked methods. From top to bottom, there are 3 rows, each representing 3 different references, including SSP, VISP, and Zeisel (zei). These scatter subplots show the PCC values of each gene predicted by ENGEP and benchmarked methods with different references. The red line is the y=x line. The values in the lower right corner are the p-value (Wilcoxon rank-sum test) and the AR value.


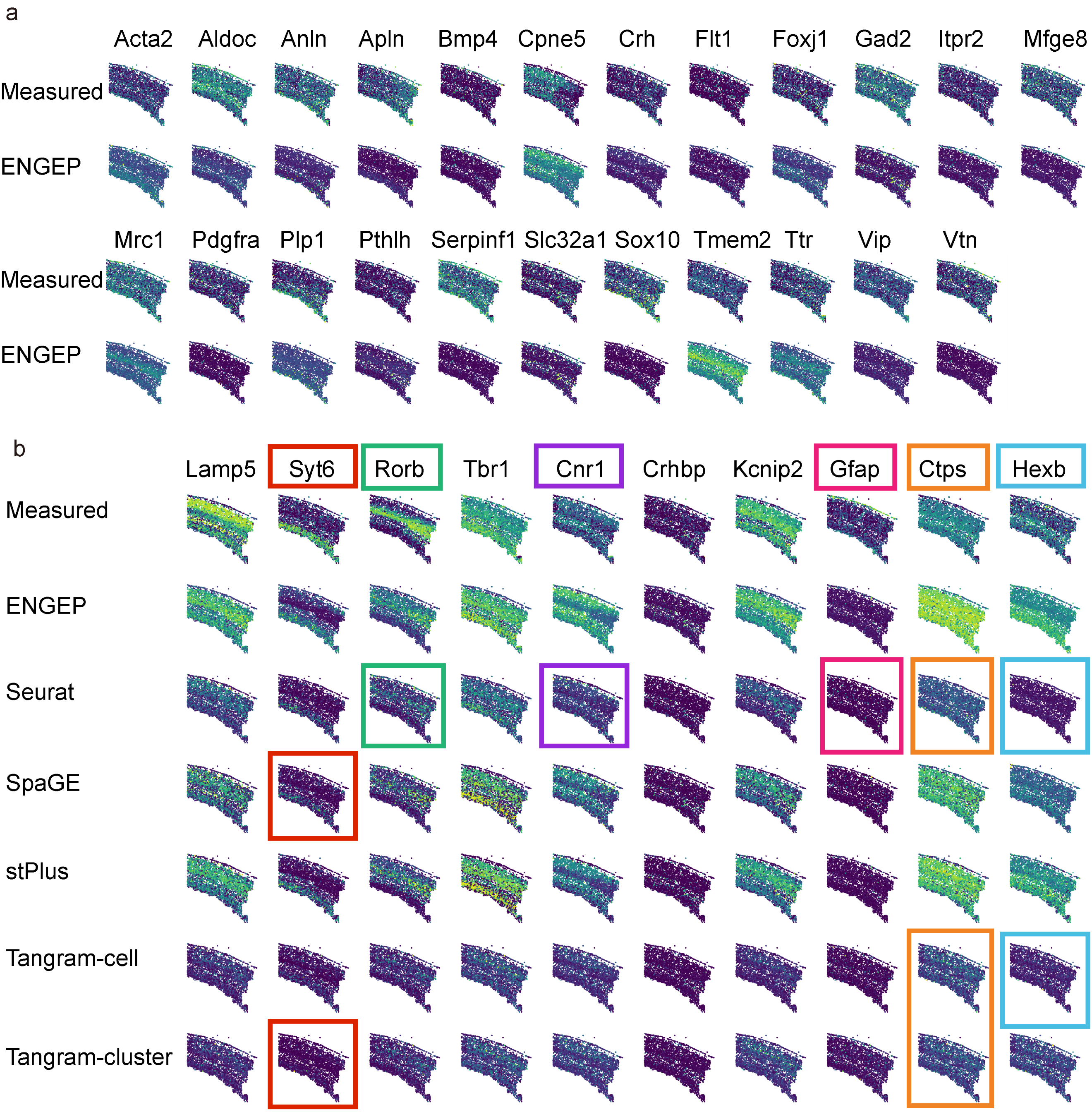


**Fig. S14. Performance comparison in osmFISH in terms of expression patterns.** a. The patterns of measured genes (first row) and the predicted genes (second row). b. The patterns of genes predicted by ENGEP and the benchmarked methods. Compared to benchmarked methods, ENGEP consistently generates expression patterns that align more closely with the measured ones. Specifically, SpaGE and Tangram-cluster result in expression patterns with lower contrast for *Syt6* which shows a high expression level in the Py L6 layer, while Seurat and Tangram produce dissimilar patterns for *Ctps* and *Hexb*. Furthermore, Seurat fails to accurately capture the layer-specific patterns for several genes, *Rorb* in Py L4 layer, *Cnr1* in Py L2-3 and Py L2-3 L5 layers, and *Gfap* in Astro. Gfap located at the top of the region.


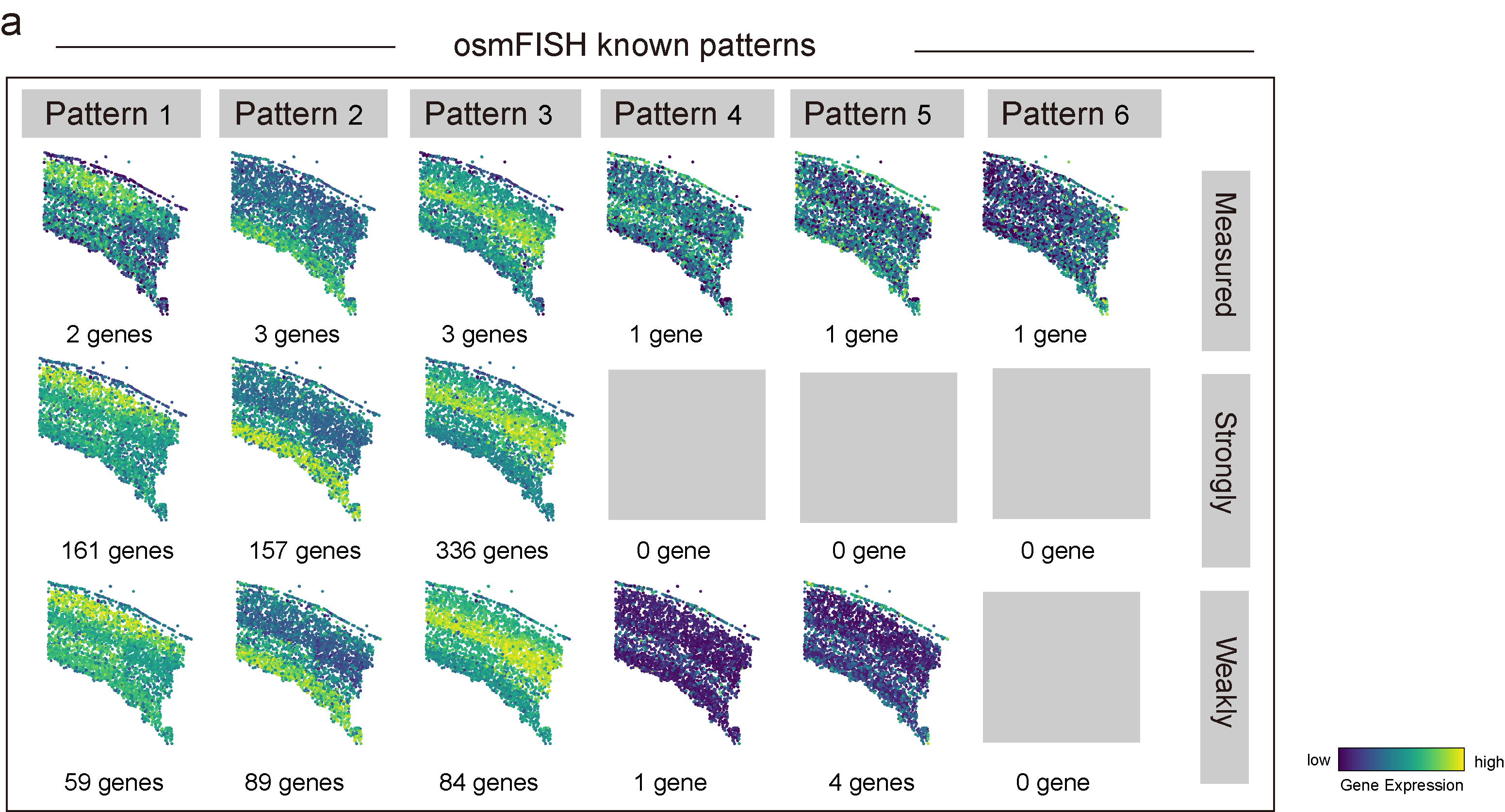


**Fig. S15. Alignment of unmeasured genes to the known patterns in osmFISH.** The top row displays the expression levels of six known patterns along with the number of genes associated with each pattern. In the second row, the expression of known patterns averaged using strongly associated genes is presented. Notably, there are no genes strongly associated with known patterns 4, 5, and 6, each of which includes only one gene. This may be due to the fact that each of them includes only one gene, which cannot capture biologically significant spatial patterns. In the third row, the expression of known patterns averaged using weakly associated genes is presented. There are no genes weakly associated with known pattern 6.

**
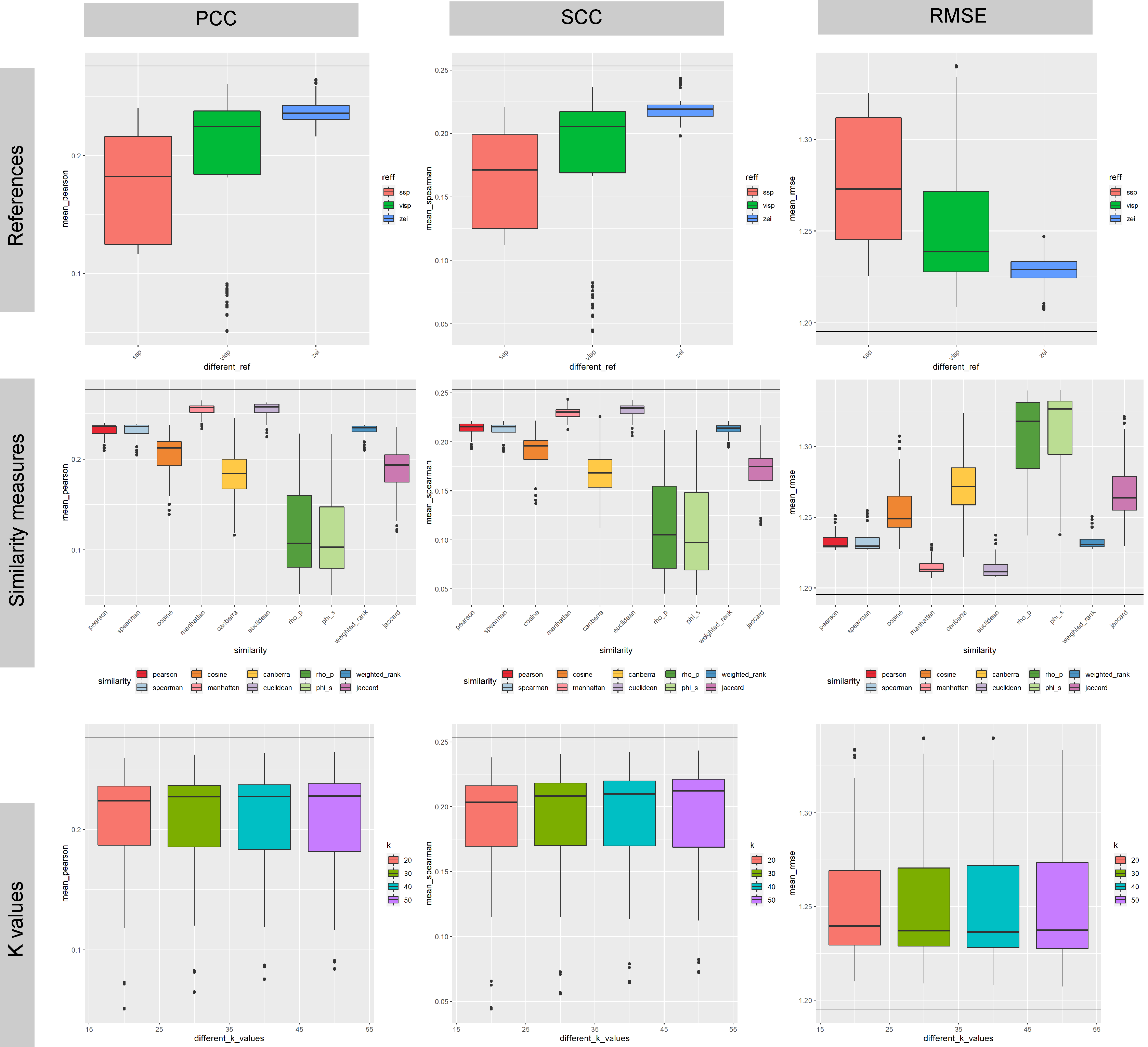
**

**Fig. S16. Comparison of performance between the ensemble result and individual base predictions in STARmap.** Each boxplot illustrates the disparity in performance when employing distinct references, similarity measures, and $k$ values. The columns signify the utilization of PCC, SCC, and RMSE metrics for assessment, while the rows present performance across diverse references, similarity measures, and $k$ values. The central black line represents the mean value of the evaluation scores for the ensemble outcome.


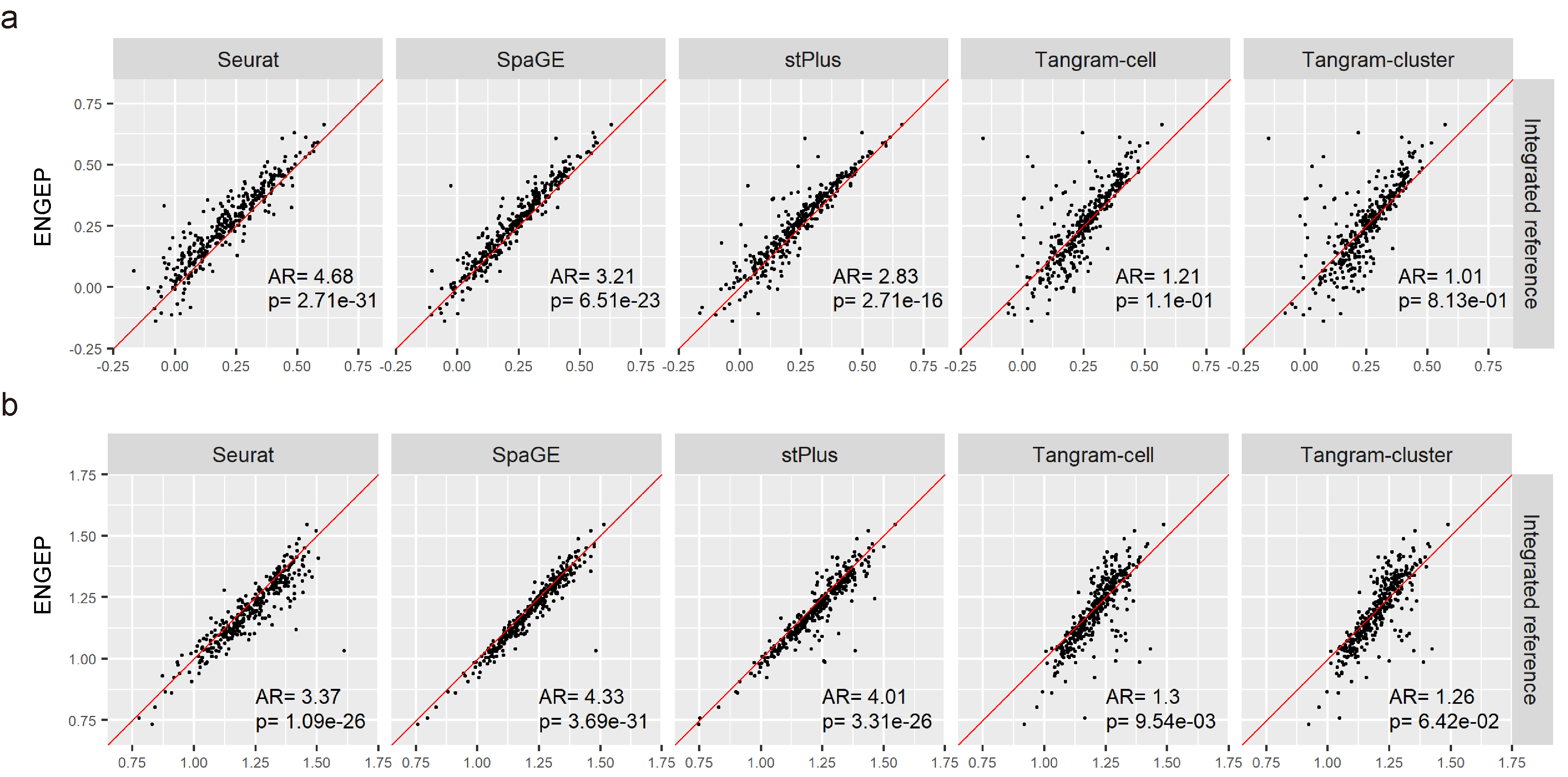


**Fig. S17. Performance comparison on predicting spatially measured genes for STARmap via 5-fold cross-validation.** The comparison encompasses ENGEP and benchmarked methods, evaluated through SCC (a) and RMSE (b) metrics. The red line signifies the y=x reference line. The included P-value highlights significant disparities between ENGEP and alternative methods based on the Wilcoxon rank-sum test. The AR value quantifies the ratio of genes predicted with superior performance by ENGEP relative to other methods.

**
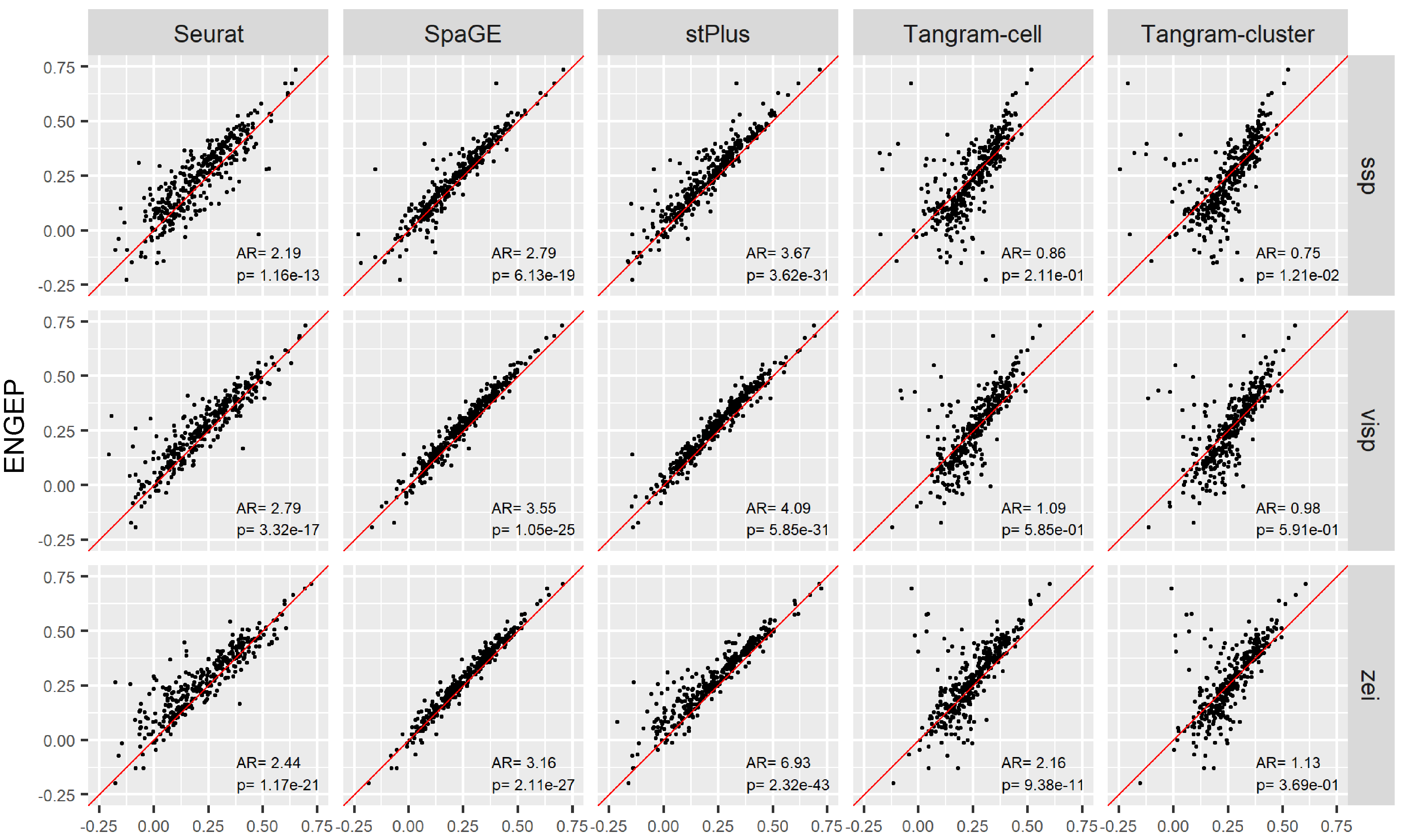
Fig. S18. Performance comparison between ENGEP and other methods by using a single reference to predict spatially measured genes for STARmap.** In this plot, from left to right, there are 5 columns representing 5 benchmarked methods. From top to bottom, there are 3 rows, each representing 3 different references, including SSP, VISP, and Zeisel (zei). These scatter plots show the PCC values of each gene across two methods in each reference. The red line is the y=x line. The values in the lower right corner are the p-value (Wilcoxon rank-sum test) and the AR value.


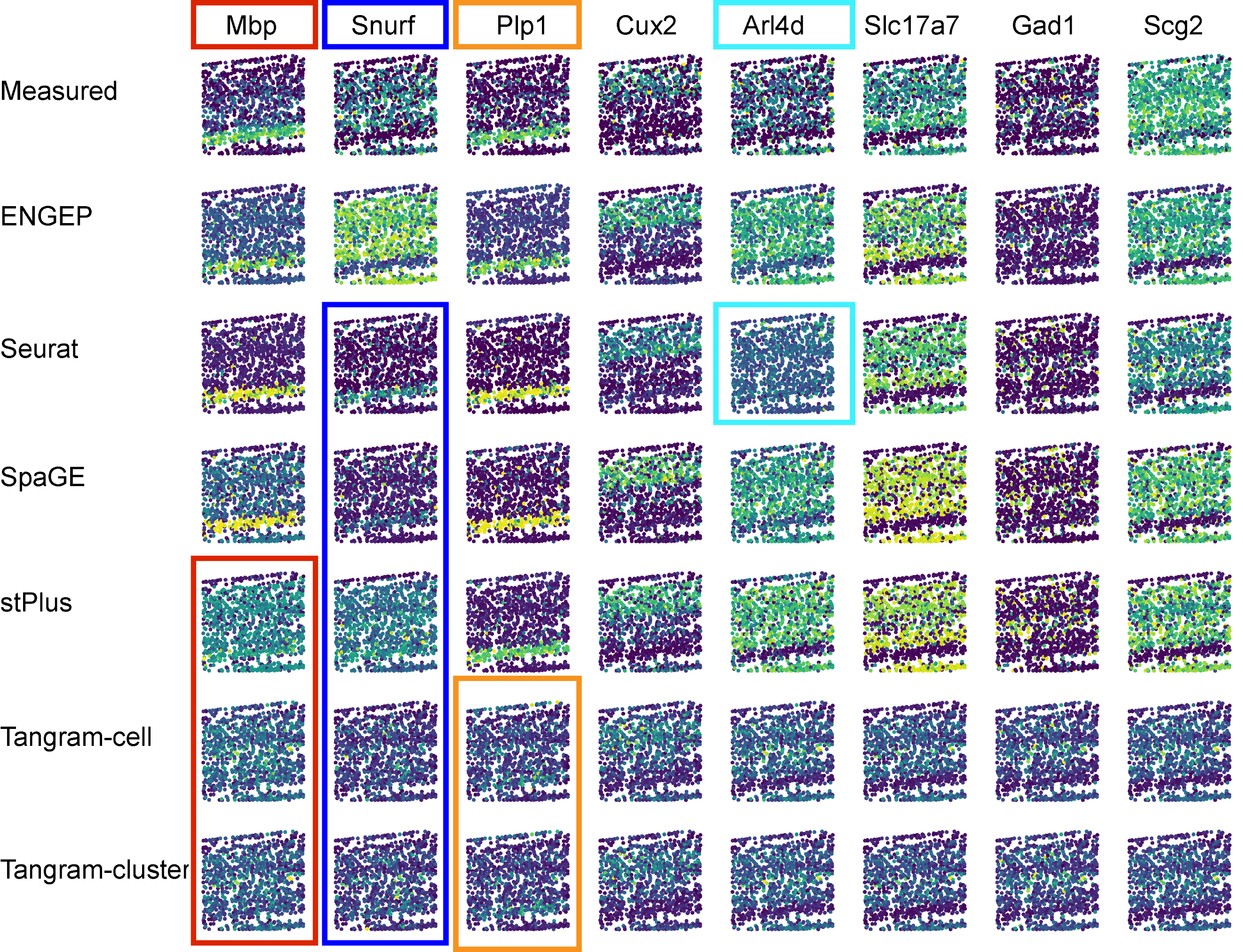


**Fig. S19. Expression patterns of measured genes in STARmap predicted by different methods.** The expression patterns predicted by ENGEP exhibit a higher degree of consistency with the observed patterns compared to benchmarked methods. For instance, stPlus and Tangram exhibit shortcomings in predicting the correct pattern for *Mbp*, which shows a high expression level in the oligodendrocyte cells. Likewise, the existing methods fail to capture the correct pattern for *Snurf*, which exhibits no expression in the oligodendrocyte cells. It is worth highlighting that Seurat and SpaGE even predict entirely contrasting patterns compared to the measured one. In the case of *Plp1*, Tangram falls short in generating the correct expression pattern, characterized by a high level of expression in oligodendrocyte cells. While Seurat struggles to predict the accurate pattern for *Arl4d*, where no expression is observed in oligodendrocyte cells.


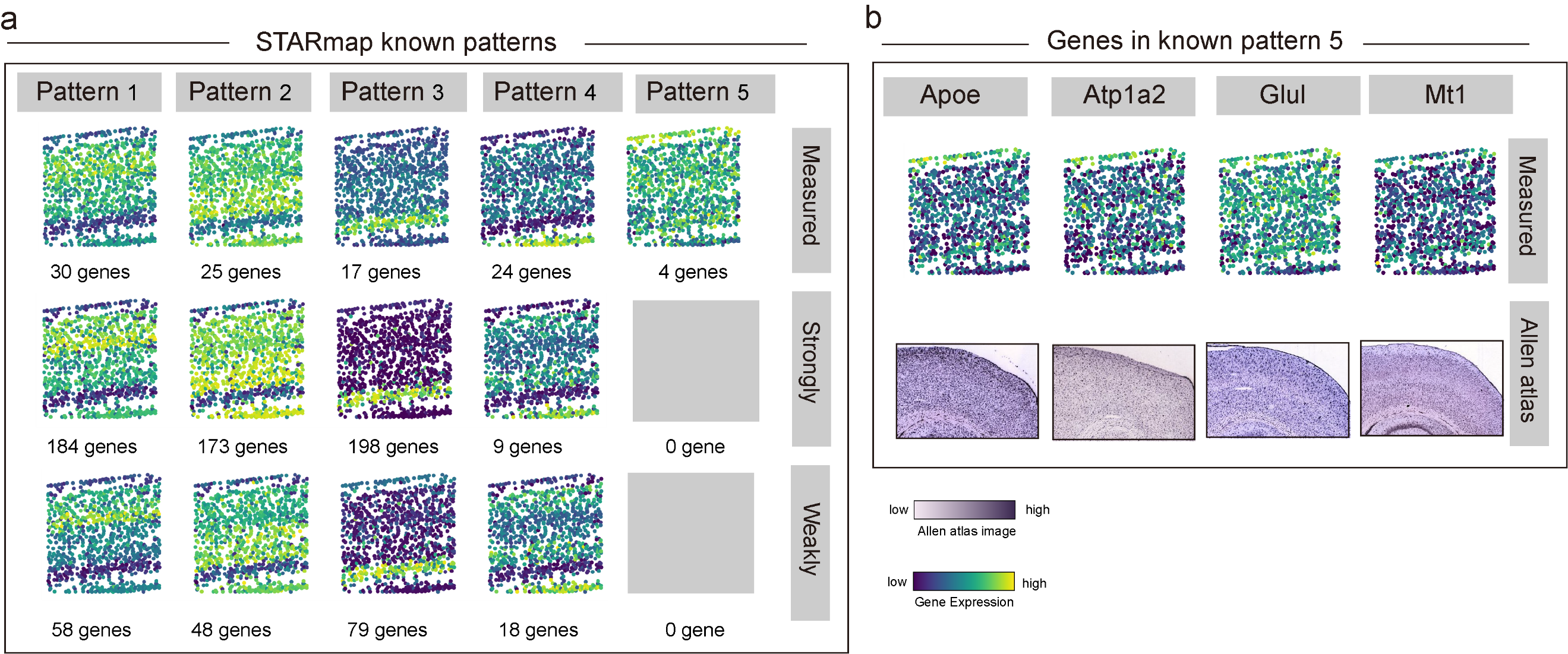


**Fig. S20. Alignment of unmeasured genes to the five known patterns in STARmap.** a. In the top row, the expression levels of five known patterns along with the number of genes associated with each pattern are displayed. The second row displays the expression of known patterns, averaged using strongly associated genes. It is important to note that there are no genes strongly associated with known pattern 5, which includes four genes. Moving to the third row, the expression of known patterns averaged using weakly associated genes is presented. There are still no genes weakly associated with known pattern 5. b. The measured expression levels and corresponding ISH images of the four constituent genes within known pattern 5 are presented. It is evident that the measured expression levels of these four constituent genes do not exhibit clear layer structures, unlike the other four known patterns, and do not closely match the ISH images. Additionally, known pattern 5 comprises a notably smaller number of genes compared to the other four known patterns, suggesting that it may possess less biological significance. These observations collectively contribute to our understanding of why no predicted genes are associated with known pattern 5.
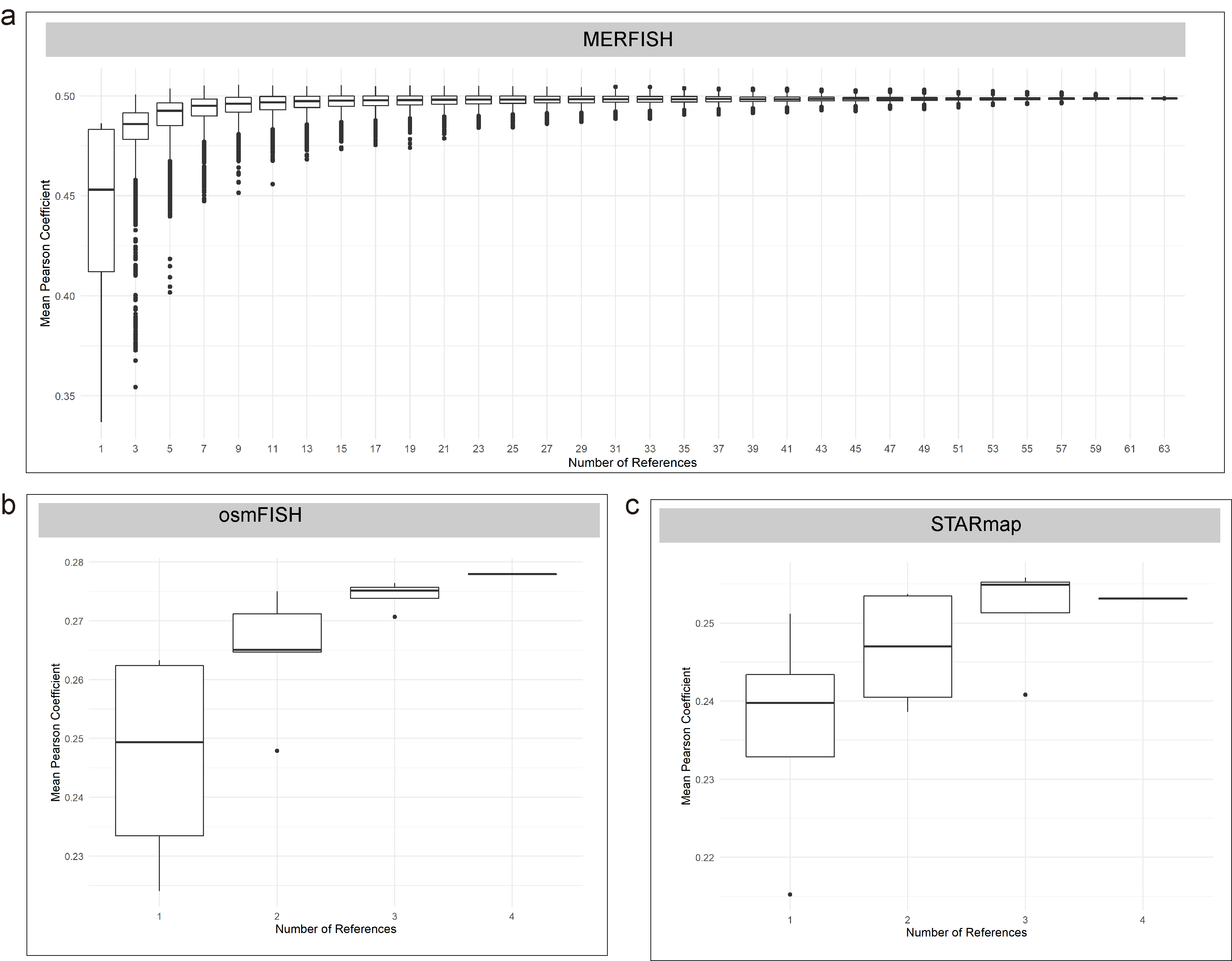


**Fig. S21. Impact of the number of references on performance.** Each plot contains boxplots illustrating ENGEP’s performance while utilizing varying numbers of sub-reference datasets for gene expression prediction. The performance is evaluated following the method described in the supplementary text. The x-axis represents the number of selected references for model training, and the y-axis represents the performance assessed through PCC values. (a) MERFISH, (b) osmFISH, and (c) STARmap.

**
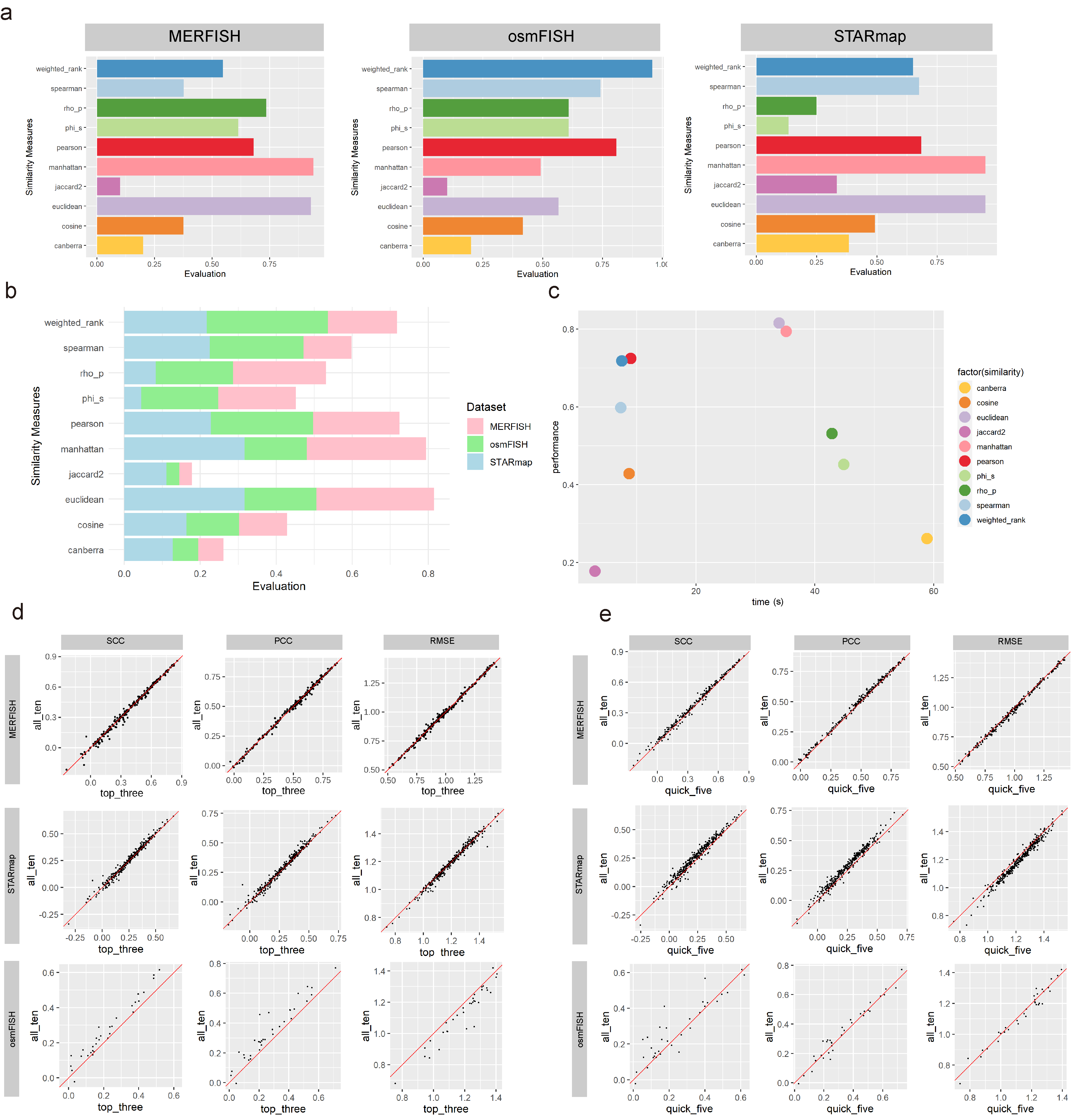
Fig. S22. Performances and computation times of different similarity measures.** a. AS scores, where a larger value indicates better performance, demonstrate the performance of ENGEP when utilizing different similarity measures in each spatial dataset. b. AS scores provide an overview of the overall performance of different similarity measures across all three datasets. c. The performance (on the y-axis) and computation times (on the x-axis) associated with various similarity measures are presented. d. The x-axis represents ensemble results obtained using the top three high-accuracy similarity measures, namely Euclidean distance, Manhattan distance, and Pearson correlation coefficient. However, these results do not surpass the ensemble results achieved by all ten similarity measures displayed on the y-axis. e. Likewise, the x-axis displays ensemble results produced by the top five computationally efficient similarity measures. Nevertheless, these results also fall short of the ensemble results attained by all ten similarity measures shown on the y-axis.

# **3 Supplementary Texts**

## 3.1 Impact of the number of references on performance

In this section, we delve into the influence of the number of reference datasets on the performance of our tool. Our investigations involve experiments on genes that are shared by both reference and query data. This is achieved through a cross-validation framework, mirroring the methodology expounded in the main text. Performance evaluation is conducted using the Pearson correlation coefficient (PCC).

For the MERFISH dataset, which comprises 63 sub-references obtained through the partitioning of the seven reference datasets, we delve into the evolution of performance as we vary the number of sub-references (denoted as $N$) within the range of 1 to 63, with an increment of 2. Notably, these sub-references exhibit a hierarchical relationship characterized by distinct $N$ values, where a reference set with a larger number of sub-references encompasses those with fewer sub-references. For instance, when $N$ is set to 1, a single dataset is randomly selected. Upon increasing $N$ to 3, the dataset selection encompasses the one previously chosen for $N$ = 1, coupled with two additional datasets selected at random. This iterative process is replicated for a spectrum of $N$ values. Due to the random nature of sub-reference dataset selection, we undertake 5000 repetitions of the experiments, presenting the outcomes using boxplots. For the osmFISH and STARmap datasets, where only four sub-references are available due to the limited collection of datasets (with a maximum of 14249 cells from three references), we vary the number of sub-references denoted as $N$. Our implementation of ENGEP spans $N$ values from 1 to 4. In each instance of $N$, we conduct the number of combinations corresponding to selecting $N$ items from a set of 4, replicating the experiments. This involves selecting datasets through distinct combinations and presenting the results visually using boxplots.

Our findings unveil a nuanced pattern: datasets with a substantial number of references (e.g., MERFISH) initially exhibit performance improvement followed by saturation (Fig. S21a), while datasets with fewer references (e.g., osmFISH and STARmap) consistently benefit from an increasing number of references (Fig. S21b, c). Therefore, we recommend that users leverage all available datasets when the number of reference datasets is limited, as this approach consistently enhances the method’s performance. In contrast, for datasets that encompass an extensive array of references, the potential for performance saturation emerges, indicating the feasibility of economizing on computational time by training the model with a reduced number of reference datasets.

While we indeed observe performance saturation with ten sub-references in the MERFISH dataset, it is important to acknowledge that this threshold could fluctuate across diverse datasets. Determining the optimal number of references poses a challenging question. Given this, in our experiments involving the MERFISH dataset, we choose to employ all available references for model training rather than selecting a subset. We recognize the opportunity to develop statistical learning theory to delve into the interplay between performance and the number of references, employing the variance-bias tradeoff theory. This pursuit aims to ascertain the tipping point at which performance saturation occurs in forthcoming endeavors.

## 3.2 Analysis of similarity measures

Here, we delve into the evaluation of various similarity measures, coupled with their computational efficiency. Our goal is to furnish readers with insights to guide the selection of suitable similarity metrics. From a study that appraises 17 metrics across five distinct scenarios, we cull ten similarity measures. These ten are chosen based on their superior performance in diverse scenarios and relatively expedited computational speed.

### 3.2.1 Performance evaluation of similarity measures

We undertake a comprehensive examination of these ten selected similarity measures in the realm of predicting spatially unmeasured genes. Employing a cross-validation setup akin to the approach outlined in the main text, we conduct experiments on genes common to both reference and query data. Given our adoption of three evaluation metrics (PCC, SCC, and RMSE), we introduce the accuracy score (AS) metric to amalgamate their outcomes, thereby gauging overall performance.

To provide further clarity, within a given spatial dataset, ENGEP generates multiple base results by employing a range of distinct similarity measures, varying k-values, and employing diverse reference datasets. For every individual base result and each evaluation metric, we evaluate gene-level performance and subsequently calculate the average score. By aggregating these scores for base results across different reference datasets and k-values, but with the same similarity measure, we establish an average performance score specific to that measure. Subsequently, we undertake the ranking of the ten similarity measures for each evaluation metric, where elevated rankings signify superior performance (larger rank values). This systematic approach yields RANKPCC, RANKSCC, and RANKRMSE rankings. By computing the mean of these three rankings, we derive the overall performance (AS). Higher AS scores correspond to superior performance.

In Fig. S22a, we present the AS scores of these ten similarity measures across three different spatial transcriptomic datasets. The findings highlight variable performance of different similarity measures across diverse datasets, with no one measure emerging as the consistent winner. For instance, the Manhattan distance excels in the MERFISH and STARmap datasets but falters in the osmFISH dataset. Conversely, weighted-rank correlation achieves its highest performance in the osmFISH dataset, while its performance is less impressive in the MERFISH dataset. These findings underscore the importance of integrating multiple similarity metrics.

Aggregating rank averages across the three spatial datasets yields the overall performance of these ten similarity measures (Fig. S22b). Notably, the Euclidean distance, Manhattan distance, and Pearson correlation coefficient emerge as the top three performers. Given the insights drawn from the superior yet efficient performance of the Euclidean distance, Manhattan distance, and Pearson correlation coefficient, we also generate ensemble results utilizing solely these three similarity measures. Subsequently, we conduct a comparison with the outcomes yielded by utilizing all ten similarity measures. The results depicted in Fig. S22d underscore a noteworthy observation: while the performance of both approaches remains comparable in the MERFISH dataset, the integration of all ten similarity measures significantly surpasses the ensemble formed by the top three similarity measures in the osmFISH and STARmap datasets. This underscores that a comprehensive integration of all ten similarity measures leads to a more robust and accurate outcome than relying solely on the top three similarity measures.

### 3.2.2 Computational time of similarity measures

We conducted a systematic evaluation of the computational time required for the ten distinct similarity measures. This comprehensive assessment encompassed various reference datasets, k-values, and spatial datasets. Fig. S22c visually illustrates the outcomes concerning both accuracy and running time for each similarity measure. Our findings emphasize the presence of a trade-off between accuracy and computational time, highlighting that no single method emerges as the optimal choice in terms of both accuracy and running time. For instance, similarity measures displaying superior performance, such as Manhattan and Euclidean distances, demand more computational resources when compared to slightly less performing measures like the Jaccard index.

Next, we investigate whether integrating the base results produced by the top five computationally efficient similarity measures, including Jaccard index, Spearman correlation coefficient, weighted rank correlation, cosine similarity, and Pearson correlation coefficient, can produce comparable performance to the ensemble results attained by all ten similarity measures. As depicted in Fig. S22e, the ensemble results yielded by the top five computationally efficient similarity measures still fall short of the ensemble results produced by all ten measures, emphasizing the efficacy of our ensemble approach. Additionally, it is pertinent to acknowledge that the computational time requirements across the various similarity measures are comparable, with the slowest measure taking up to six times longer than the fastest one. Consequently, we recommend users leverage all available similarity measures. Doing so ensures accurate predictions, model generalization, and a manageable increase in running times.

## 3.3 Identification of cell types colocalized with spatial patterns

In this section, we present our approach for identifying cell types that strongly co-localize with specific spatial gene expression patterns. The central premise guiding our approach is straightforward: cell types located in spatial proximity to a given pattern should exhibit a positive correlation with its gene expression profile.

Our method systematically incorporates cell types into an evolving “cell type set” based on their correlation with the pattern. Initially, this set is empty, but we progressively populate it with cell types as our analysis proceeds. We begin by calculating the Pearson correlation between the pattern’s gene expression and the binary representation of each cell type’s presence within the spatial context. Starting with the cell type showing the highest correlation, we iteratively add more cell types to the set and recalculate the correlation of the evolving set with the pattern. Importantly, when a set comprises multiple cell types, we encode the binary cell type vector such that a value of 1 denotes a cell belonging to one of the cell types in the set, and 0 otherwise. Throughout this iterative process, we closely monitor changes in correlation. When the increase in correlation falls below a user-defined threshold (1.05 by default), it signals that further additions of cell types no longer significantly enhance the correlation. Consequently, the algorithm identifies this set of cell types as those closely co-located with the spatial pattern. For detail, please refer to Algorithm 1. In Additional file 2: Tables S2, S4, and S6, the cumulative correlation as more cell types are iteratively added, along with the identified cell types closely co-localizing with the spatial patterns for MERFISH, osmFISH, and STARmap, respectively.

It is essential to note that a single spatial pattern can encompass multiple distinct cell types, each contributing to specific biological functions. Therefore, a spatial pattern may simultaneously co-localize with multiple cell types. Theoretically, as we progressively include cell types into the evolving set, the correlation trend may exhibit an initial increase followed by a decrease. This trend aligns with the idea that initially included cell types are positively correlated with the spatial pattern, while subsequently added cell types may exhibit negative correlations due to their different spatial distributions. This correlation dynamics, governed by changes in the composition of the evolving cell type set during the analysis, guides our algorithm in determining which cell types closely co-localize with the spatial pattern.

| **Algorithm 1** |
| --- |
| **Input:**  $y$: Gene expression vector representing the spatial pattern of interest;  $z$: Binary matrix representing the presence (1) or absence (0) of different cell types within the spatial context;  $\tau$: User defined threshold for change in correlation. We set $\tau=1.05$ by default.  **Output:**  $celltype$: Set of cell types closely co-located with the spatial pattern   1. Calculate the Pearson correlation between y and each column (cell type) in $z$, storing results in $C_{yz}$. 2. Sort the elements of $C_{yz}$in decreasing order and keep track of their original indices, storing the sorted indices in $C_{idx}$. 3. Initialize:   $n_{c}:$ (array of length equal to the number of cell types with all elements set to 0)  $C_{label}:$ (a binary vector representing the cell types included in the set, starting with the first cell type)  $C_{change}:$ (an array to keep track of changes in correlation)   1. Calculate the correlation between $y$ and the first cell type $(z\left[ ,C_{idx}\left[ 1 \right] \right])$ and store it in $n_{c}\left[ 1 \right].$ 2. For $i = 2$ to length$(C_{idx}) - 1$:   a. Add the cell type $(z[, C_{idx}[i]])$ to the $C_{label}$vector  b. Calculate the correlation between $y$ and the current set of cell types ($C_{label})$ and store it in $n_{c}[i]$  c. Calculate the change in correlation relative to the previous iteration:  $C_{change}[i-1] = n_{c}[i] / n_{c}[i-1]$  d. If $C_{change}[i-1] < \tau$:  - set $celltype$ to the indices of cell types up to the current index:  $celltype = idx[1:(i-1)]$  - Exit the loop   1. **Return** the $celltype$ set as the output. |
