# Supplementary material for: ENGEP: advancing spatial transcriptomics with accurate unmeasured gene expression prediction
Source: Genome Biol. 2023 Dec 21;24:293. doi: 10.1186/s13059-023-03139-w (PMC10734203; doi:10.1186/s13059-023-03139-w)
Supplement: Supplementary file 3 — Additional file 3. Review history. [file 13059_2023_3139_MOESM3_ESM.docx]

Review History

**First round of review**

**Reviewer 1**

**Were you able to assess all statistics in the manuscript, including the appropriateness of statistical tests used?**

**Yes:** I did not see any clear issues with the statistics in the manuscript.

**Were you able to directly test the methods?**

No

**Comments to author:**

The authors developed a new method to impute gene expression values by integrating single cell and spatial transcriptomics data. There are already numerous methods, many of them listed in the manuscript, to perform this task. The authors compare against a few of the most popular tools using a cross validation strategy where their method performed favorably. My main concerns are about the scope of the contribution. Specifically, I don't know if this methodology represents a large enough technical contribution in terms of the methodology, there are already many tools available to perform this task, and there was not any biological discovery or novel dataset generated.

The author's method is functionally a bootstrap aggregation of kNN models. This does not seem to be a huge technical contribution to the field given that these models are simple to implement in a number of programming languages like R and Python.

There are dozens of tools available to impute missing values in spatial transcriptomics data so there is not a clear need for incremental improvement given many of these methods perform well already.

Many of the other methods that also impute missing values have extensive additional functionality besides the imputation. Seurat for instance is the most popular package for scRNA-seq and ST analysis with numerous methods for preprocessing, clustering, integration, and visualization. Tangram can perform multiomic integration experiments and deconvolution experiments.

There is not a clear biological discovery that was made using their newly available algorithm which could not have been made using existing tools.

**Reviewer 2**

**Were you able to assess all statistics in the manuscript, including the appropriateness of statistical tests used?**

**Yes:** Statistical methods are appropriate. Required significances are reported.

**Were you able to directly test the methods?**

No

**Comments to author:**

While imaging-based spatial transcriptomics techniques offer valuable spatial and gene expression data at the single-cell level, their current limitation lies in the restricted profiling of a limited number of genes per cell. To overcome this challenge, Yang and Zhang developed a new tool, ENGEP, to predict expression levels of unmeasured genes in imaging-based spatial transcriptomics data. ENGEP utilizes ensemble learning to combine diverse reference datasets and prediction methods, surpassing previous approaches that rely on a single reference dataset or prediction method. The performance of ENGEP was assessed using three spatial transcriptomics datasets obtained from various technologies. The results indicated that ENGEP outperforms state-of-the-art methods while also demands lower computational resources. In my opinion, the presented tool is intriguing, and the manuscript is well-structured and well-written. I have a few questions regarding this paper.

(1) In the method section (Step 1: Generating base results), the authors described how ENGEP handles large reference datasets. They randomly partitioned each large scRNA-seq dataset into multiple equal-sized sub-datasets, which are then referred to as sub-reference datasets. However, it is unclear in the paper whether the original large scRNA-seq datasets and the divided sub-datasets are both referred to as reference datasets. To avoid confusion, I recommend that the authors clarify this terminology in their statements. Additionally, it would be helpful to include a new figure in the supplementary data to illustrate the process of dividing the large scRNA-seq datasets into small sub-datasets.

(2) The authors have used ten different similarity measures. Why were these ten similarity measures selected in this study? Are they complementary or are there some measures that consistently perform better while others do not? Can we consider using subsets of these measures? Additionally, in the evaluation of performance (Figures S2, S7, and S11), it would be helpful if the authors visually differentiate the results based on the specific similarity measures and values of k, using different colors or shapes for comparison, which would enhance the understanding of the complementarity among these measures.


(3) It would be informative to discuss the computational efficiency of each similarity measure. Are there any measures that are particularly slow or computationally demanding? If so, it would be valuable for the authors to highlight this aspect. Furthermore, the authors could investigate the effect of excluding slow similarity measures on the overall performance. Are there specific measures that, when excluded, lead to a significant decrease in performance? Understanding the trade-off between computational efficiency and performance would provide practical insights for researchers and practitioners using these similarity measures in their analyses.

(4) For the MERFISH dataset, the authors specifically selected the "mouse2 slice300" slice from the second mouse. However, to evaluate the generalization ability and robustness of the proposed methods, it would be advantageous for the authors to expand their experiments to include a larger number of slices. This could involve incorporating multiple slices from the same mouse or even different mice. By diversifying the datasets, the findings can be validated across a wider range of samples, thereby enhancing the confidence in the generalizability of the results.

(5) The authors employed manifold mixup to assign the unmeasured genes into groups categorized as known archetypes or novel archetypes. Based on the definitions, it appears that the values of $g_{ij}$ are random due to the random sampling of $\lambda$ from a uniform distribution between 0 and 1. Consequently, the threshold used to distinguish genes as known or novel archetypes, which is determined by the average likelihood scores of all synthesized genes $g_{ij}$, could potentially be random. This raises the question of whether the threshold would vary significantly with different generations of synthesized genes and if it would consequently affect the partitioning of unmeasured genes into known and novel archetype groups. It would be valuable for the authors to address this concern and investigate the potential impact of different generations of synthesized genes on the threshold and resulting partitioning.
===
Please consider the following questions:
- Are the methods appropriate to the aims of the study, are they well described, and are necessary controls included?
- Are the conclusions adequately supported by the data shown?
- Are sufficient details provided to allow replication and comparison with related analyses that may have been performed?
- Is the method likely to be of broad utility? Is any software component easy to install and use? Please indicate briefly the novel features and/or advantages of the method, and/or please reference the relevant publications and which methods, if any, it should be compared with.
- Is the paper of broad interest to others in the field, or of outstanding interest to a broad audience of biologists?

**Reviewer 3**

**Were you able to assess all statistics in the manuscript, including the appropriateness of statistical tests used?**

**Yes:** No additional comments here.

**Were you able to directly test the methods?**

No

**Comments to author:**

The authors presented ENGEP, a method that uses multiple reference datasets and parameters to predict the gene expression levels in image-based spatial transcriptomics data. This method appears promising and can potentially be of interest to users. However, there are multiple concerns that need to be addressed.

Major concerns:

1. In Figs. 3d, 5d, and 7d, the authors showed novel archetypes and the cell types they may correspond to. First, the cell types that are colored in these figures seem to be selected cell types. Please clarify how these cell types are selected. Second, since the pattern of these novel archetypes tend to be much noisier than the known archetypes, it is hard to draw correspondence between the novel archetypes and the highlighted cell types. The authors can consider providing quantitative measures to show that the highlighted cell types are the ones that match the novel archetypes better than other cell types.

2. In Figs. 3e, 5e, and 7e, the authors showed Gene Ontology analysis terms of the genes in the novel archetypes. First, the authors did not mention what background gene list was used for GO analysis. In case the complete gene list of the corresponding species is used, the authors should re-do this analysis with the background gene list that contains only the expressed genes in the dataset. Second, the presented GO terms do not sufficiently support the proposed cell types for the novel archetypes. Further discussions with references need to be provided to support the consistency between the top GO terms and the selected cell types that correspond to the novel archetypes.

3. In multiple figures where predicted gene expression patterns are compared with ISH images, it is hard to observe the claimed consistency. Examples include Fig. 2d, Fig. 3c-d. The authors should make an effort to make this consistency easier to observe. Some suggestions are: First, the ISH images can be shown in the same direction as the images of predicted gene expression. Second, please add color legends (color code) that shows the correspondence between colors and gene expression for all ISH and predicted gene expression images. Third, highlight certain parts in the images which can help show the consistency.

4. In order to use this tool, users need to collect multiple reference datasets. One question is how many datasets are sufficient. Is it the more the better, or can it reach saturation at some point? Please provide discussion on this which can be helpful for users.

5. There is no discussion on how to set parameters for ENGEP or which hyper parameters are used to generate the presented results. The parameters include those used in pre-processing steps. Please add this discussion to guide users for parameter selection.

6. Lines 323-326: the explanation of why no genes are assigned to known archetype 5 is not supported. To verify that this is indeed due to the low gene detection sensitivity, the authors can compare the detection sensitivity of genes in known archetype 5 and genes in other known archetypes.

Minor:

Fig. S1: dimodality → bimodality
Line 300: "a major cell type" - please specify the cell type.
Fig. 2c: nagative → negative

**Authors Response**

**Point-by-point responses to the reviewers’ comments:**

**Referee #1:**

The authors developed a new method to impute gene expression values by integrating single cell and spatial transcriptomics data. There are already numerous methods, many of them listed in the manuscript, to perform this task. The authors compare against a few of the most popular tools using a cross validation strategy where their method performed favorably. My main concerns are about the scope of the contribution. Specifically, I don’t know if this methodology represents a large enough technical contribution in terms of the methodology, there are already many tools available to perform this task, and there was not any biological discovery or novel dataset generated.
**Response:**

We appreciate the thoughtful feedback provided by the referee regarding our work. In response to the concerns raised, we have diligently considered each point, and we would like to provide a more refined and detailed response. Please refer to the specific responses provided below for a more comprehensive understanding of our work in terms of technical contribution in methodology, complementing existing tools, and biological discovery.

1. The author’s method is functionally a bootstrap aggregation of kNN models. This does not seem to be a huge technical contribution to the field given that these models are simple to implement in a number of programming languages like R and Python.
**Response:**

We appreciate the referee’s thoughtful comments and have given careful consideration to the points raised. While our ENGEP method, at its core, employs an aggregation of k-nearest neighbors (KNN) regression models, we believe it provides a substantial and multifaceted technical contribution to the field for several compelling reasons:

**(1) Innovative approach**: While our ENGEP method utilizes an aggregation of KNN models at its core, it represents a substantial technical contribution for several reasons. First, it addresses a significant challenge in the spatial transcriptomics field, which is the prediction of unmeasured gene expressions in spatial data generated by imaging-based techniques targeting only a subset of genes. Existing methods often rely on complex mathematical models for matching reference and spatial datasets or correcting batch effects, making them challenging to implement and computationally intensive. ENGEP offers an innovative ensemble technique based on KNN regression, which seamlessly integrates a diverse array of references, similarity measures, and k values. This approach eliminates the need for manual reference dataset selection and simplifies the tool, making it accessible to a broader user base. Moreover, the ensemble method significantly enhances ENGEP’s overall performance, resulting in superior prediction accuracy, robustness, computational efficiency, and user-friendliness compared to existing methods.

**(2) Outstanding accuracy**: To comprehensively evaluate ENGEP in terms of prediction accuracy, we have conducted a series of experiments using datasets generated by various spatial technologies (MERFISH, osmFISH, and STARmap). We benchmark ENGEP against five state-of-the-art methods across 19 experiments, encompassing different tissues, samples, numbers of genes, cells, and scenarios involving both single and multiple reference datasets. We employ three evaluation metrics, namely Pearson correlation coefficient (PCC), Spearman correlation coefficient (SCC), and root mean square error (RMSE), to quantitatively assess accuracy. The experimental results consistently demonstrate that ENGEP outperforms existing methods, firmly establishing its unique advantage in predictive accuracy.

**(3) Robustness across datasets**: As previously mentioned, we have conducted a diverse set of experiments under various scenarios, revealing ENGEP’s exceptional stability across a wide range of datasets. In contrast, many existing methods display dataset-dependent variations in their predictive capabilities. As discussed in the “Discussion” section, these variations occur because previous tools rely on single reference datasets and prediction methods. This limitation results in inconsistent performance across datasets. For instance, SpaGE performs well with MERFISH and osmFISH datasets but faces challenges with STARmap, while Tangram Cell and Tangram Cluster excel with STARmap but struggle with other datasets. ENGEP’s robustness is attributed to its ensemble learning approach, which seamlessly integrates information from multiple reference datasets and employs diverse prediction methods. This multifaceted strategy not only ensures superior accuracy but also maintains consistent performance across different data contexts.

**(4) Efficient computational resource usage**: Many existing tools heavily rely on complex mathematical models to match reference and spatial datasets or correct batch effects, often demanding significant computational resources. In contrast, ENGEP employs a KNN model to generate base predictions, which is computationally less intensive. Furthermore, these base predictions can be computed in parallel, reducing processing time when multiple computer cores are available. These characteristics make ENGEP suitable for efficiently handling large datasets. Fig. 8 illustrates ENGEP’s superior computational efficiency, including execution time and memory utilization. Its scalability further positions ENGEP as an ideal choice for managing the ever-expanding volumes of single-cell and spatial transcriptomics data.

**(5) User-friendly design**: ENGEP’s user-friendly design is a significant contribution, making it accessible to researchers without strong backgrounds in mathematics and computer science. Many existing tools require users to manually select optimal reference datasets and fine-tune complex hyperparameters, which can be daunting for non-experts. In contrast, ENGEP’s ensemble approach automatically assigns weights to references and does not require intricate parameter tuning. The simplicity of its R package ensures that researchers can readily harness its capabilities to enhance spatial transcriptomics data analysis without being encumbered by technical intricacies.

In summary, while the foundational concept of ENGEP may appear straightforward, its multifaceted contributions collectively establish it as a valuable asset in the field of spatial transcriptomics. ENGEP’s combination of exceptional accuracy, robustness, computational efficiency, user-friendliness, and innovative problem-solving approaches positions it as an indispensable addition to the spatial transcriptomics researcher’s toolkit. We firmly believe that ENGEP not only elevates the quality of gene expression predictions but also serves as a valuable resource, facilitating significant advancements in the realm of spatial transcriptomics research.

2. There are dozens of tools available to impute missing values in spatial transcriptomics data so there is not a clear need for incremental improvement given many of these methods perform well already. Many of the other methods that also impute missing values have extensive additional functionality besides the imputation. Seurat for instance is the most popular package for scRNA-seq and ST analysis with numerous methods for preprocessing, clustering, integration, and visualization. Tangram can perform multiomic integration experiments and deconvolution experiments.

**Response:**

We genuinely appreciate the referee’s thoughtful consideration of existing tools for imputing missing values in spatial transcriptomics data. It is crucial to acknowledge that, despite the availability of these methods, significant opportunities for improvement persist in several critical aspects, encompassing prediction accuracy, robustness, computational efficiency, and user-friendliness. Firstly, as evidenced in Figs. 2, 4, and 6, existing tools often fall short in terms of achieving high prediction accuracy. This indicates a substantial scope for enhancing accuracy in gene expression prediction. Secondly, existing methods are built upon varying strategies and models, requiring meticulous parameter tuning for specific datasets. This variability in approach leads to inconsistent performance across datasets, rendering these tools less robust. Thirdly, some existing tools encounter considerable runtime and memory usage challenges when dealing with extensive reference datasets (Fig. 8), limiting their usability as single-cell and spatial transcriptomics data continue to grow. Lastly, the requirement for users to carefully select reference datasets and fine-tune hyperparameters poses a significant hurdle, especially for non-experts in the field. To address these challenges, we have developed ENGEP. The experimental results presented in this manuscript underscore ENGEP’s ability to effectively tackle these issues, excelling in terms of accuracy, robustness, computational efficiency, and user-friendliness.

While other methods may offer extensive additional functionality, ENGEP’s primary focus is on gene expression prediction in spatial transcriptomics data. However, inspired by the referee’s constructive comments, we plan to expand ENGEP’s capabilities to include a wide range of downstream analyses. This expansion will encompass exploring spatial gene expression patterns, conducting spatial trajectory inference, investigating cell-cell communication patterns, and constructing spatially resolved gene regulatory networks, all guided by the predictions generated by our ensemble methods. This enhancement will significantly broaden ENGEP’s utility for addressing various aspects of spatial transcriptomics data analysis. We have incorporated this clarification in the “Discussion” section to emphasize the potential for future enhancements in ENGEP’s functionality.

3. There is not a clear biological discovery that was made using their newly available algorithm which could not have been made using existing tools.

**Response:**

We appreciate the referee’s emphasis on the importance of biological discovery in scientific research. While our primary objective in this work is the development and evaluation of the ENGEP method for predicting gene expression, we understand the significance of demonstrating its utility for biological discovery. We have made additional efforts to explore potential biological discoveries using ENGEP, as outlined below:

**(1) Comparing gene expression patterns predicted by different methods**: To emphasize how ENGEP represents a significant advancement over existing methods by revealing novel biological insights, we conduct a detailed visual comparison of predicted gene expression patterns from different methods. Our analysis reveals instances where ENGEP accurately captures gene expression patterns, while certain existing methods produce predicted patterns that significantly diverge from the actual measurements. In the MERFISH dataset, ENGEP stands out by accurately preserving the layer structure for genes like *Marcksl1* and *Reln*. Moreover, it excels in producing more distinct spatial patterns for genes such as *Enpp6* and *Pcdh8*. These accomplishments surpass the capabilities of certain existing methods, as illustrated in Additional file 1: Fig. S9. This trend is consistently observed in the osmFISH and STARmap datasets (Additional file 1: Fig. S14b, Fig. S19). These compelling findings validate ENGEP's progress beyond existing methods, underscoring its capacity to unveil novel biological insights.

**(2) Correcting low-quality measured genes**: We have embarked on an exploration of novel biological discoveries by utilizing ENGEP’s predictions. Our efforts begin with the enhancement of data quality for low-quality genes in the MERFISH and STARmap datasets. The validity of predicted expression patterns is confirmed through comparisons with ISH images in the Allen Brain Atlas or those generated by other highly sensitive spatial technologies (Fig. 2d, Fig. 6c, and Fig. 6d).

**(3) Exploring novel spatial patterns:** ENGEP is further harnessed to predict the expression levels of spatially unmeasured genes, unveiling previously undiscovered spatial patterns concealed within them. **It is noteworthy that previous studies did not conduct experiments aimed at identifying these novel spatial patterns or confirming their functions.** In total, we identify three novel patterns in the MERFISH dataset (Fig. 3), two in the osmFISH dataset (Fig. 5), and three in the STARmap dataset (Fig. 7). A comprehensive analysis is undertaken to explore the biological significance of these newly identified patterns. This includes selecting representative genes from each pattern and comparing their expression with corresponding ISH images, investigating associations with specific cell types, and conducting Gene Ontology enrichment analysis to reveal associated biological processes. These analyses serve to confirm the biological significance of the identified novel spatial patterns.

In summary, while our primary focus in this work is the development and evaluation of the ENGEP method for gene expression prediction, we respond to the referee’s concerns by demonstrating that ENGEP can provide valuable support for biological discovery. We showcase how ENGEP outperforms existing tools in capturing certain expression patterns and makes new discoveries based on the predicted expression of unmeasured genes.

**Referee #2:**

While imaging-based spatial transcriptomics techniques offer valuable spatial and gene expression data at the single-cell level, their current limitation lies in the restricted profiling of a limited number of genes per cell. To overcome this challenge, Yang and Zhang developed a new tool, ENGEP, to predict expression levels of unmeasured genes in imaging-based spatial transcriptomics data. ENGEP utilizes ensemble learning to combine diverse reference datasets and prediction methods, surpassing previous approaches that rely on a single reference dataset or prediction method. The performance of ENGEP was assessed using three spatial transcriptomics datasets obtained from various technologies. The results indicated that ENGEP outperforms state-of-the-art methods while also demands lower computational resources. In my opinion, the presented tool is intriguing, and the manuscript is well-structured and well-written. I have a few questions regarding this paper.
**Response:**

We appreciate the referee's positive assessment of our manuscript and careful consideration of the analytical details. Based on the suggestions provided, we have made revisions to improve the quality of our manuscript.

(1) In the method section (Step 1: Generating base results), the authors described how ENGEP handles large reference datasets. They randomly partitioned each large scRNA-seq dataset into multiple equal-sized sub-datasets, which are then referred to as sub-reference datasets. However, it is unclear in the paper whether the original large scRNA-seq datasets and the divided sub-datasets are both referred to as reference datasets. To avoid confusion, I recommend that the authors clarify this terminology in their statements. Additionally, it would be helpful to include a new figure in the supplementary data to illustrate the process of dividing the large scRNA-seq datasets into small sub-datasets.
**Response:**

We appreciate the referee's constructive suggestion. In our revised manuscript, we distinguish between the original reference dataset and the partitioned dataset by referring to the latter as “sub-reference datasets”. In the “Overview of ENGEP” section, we have added the following sentence to highlight this: “To manage large-scale reference datasets, we first partition each substantial reference dataset into smaller sub-reference datasets”. In the “Methods” section, we also clarify this using the following text: “To adapt ENGEP to handle large reference datasets, each scRNA-seq dataset is randomly partitioned into $\left\lceil n/n_{0} \right\rceil$ equal-sized sub-reference datasets, where $n$ is the number of cells in the scRNA-seq dataset and $\left\lceil\cdot\right\rceil$ is the ceiling function.”

Based on the referee’s suggestion, we have revised Fig. 1 in the main text to provide a more detailed illustration of the process of dividing the large scRNA-seq datasets into small sub-datasets. We believe this revision will help clarify the terminology and assist users in better understanding our methods.

(2) The authors have used ten different similarity measures. Why were these ten similarity measures selected in this study? Are they complementary or are there some measures that consistently perform better while others do not? Can we consider using subsets of these measures? Additionally, in the evaluation of performance (Figures S2, S7, and S11), it would be helpful if the authors visually differentiate the results based on the specific similarity measures and values of $k$, using different colors or shapes for comparison, which would enhance the understanding of the complementarity among these measures.
**Response:**

We appreciate the referee’s thoughtful questions regarding our choice of similarity measures and the presentation of our results. These ten similarity measures are selected based on a study [1] that systematically evaluates 17 association measures across various tasks in single-cell transcriptomics. We prioritize measures that demonstrate strong performance and computational efficiency, aligning with ENGEP’s design. In the “Overview of ENGEP” section, we provide clarification with the following text: “These ten similarity measures are chosen based on a study [14], which evaluates different association measures in single-cell transcriptomics. We prioritize measures that show strong performance and computational efficiency, making them suitable for integration into ENGEP.”

Regarding the consistent performance of these measures, we conduct a comprehensive examination, and the results are reported in Additional file 1: Figs. S2, S11, S16, and S22, as well as Additional file 1: Section S3.2.1. Our findings reveal that no single similarity measure consistently outperforms others across all datasets. This highlights the necessity of amalgamating results from diverse similarity measures, reinforcing the value of our ensemble approach. We also have added text in the “Discussion” section to emphasize this point (the third paragraph).

Additionally, we have conducted experiments to explore whether using subsets of these ten measures can produce results comparable to using all ten measures. In Additional file 1: Section S3.2.1, we generate ensemble results using only three similarity measures: Euclidean distance, Manhattan distance, and Pearson correlation coefficient, which demonstrate relatively strong performance (Additional file 1: Fig. S22b). Subsequently, we compare these results with those obtained using all ten similarity measures. The outcomes, depicted in Fig. S22d, underscore a significant observation: while the performance remains comparable for the MERFISH dataset, the integration of all ten similarity measures significantly surpasses the ensemble formed by the top three measures in the osmFISH and STARmap datasets. This emphasizes the importance of comprehensively integrating all ten similarity measures for robust and accurate predictions.

Furthermore, we acknowledge the referee’s suggestion to visually differentiate the results based on specific similarity measures and $k$ values. Considering the large number of base results for the MERFISH data, for clarity of representation, we use box plots to illustrate the variations in base results from three aspects: different reference datasets, different similarity measures, and different values of $k$. The new plots are presented in Figs. S2, S11, and S16 in Additional file 1.

Reference

[1] Skinnider MA, Squair JW, Foster LJ. Evaluating measures of association for single-cell transcriptomics. Nat Methods. 2019;16:381–6.

(3) It would be informative to discuss the computational efficiency of each similarity measure. Are there any measures that are particularly slow or computationally demanding? If so, it would be valuable for the authors to highlight this aspect. Furthermore, the authors could investigate the effect of excluding slow similarity measures on the overall performance. Are there specific measures that, when excluded, lead to a significant decrease in performance? Understanding the trade-off between computational efficiency and performance would provide practical insights for researchers and practitioners using these similarity measures in their analyses.

**Response:**

We appreciate the referee’s suggestion regarding the computational efficiency of the similarity measures used in our study. In response, we have conducted experiments to study the trade-off between computational efficiency and performance in the revised manuscript (Additional file 1: Section S3.2.2).

To begin with, we conduct a systematic evaluation of the computational time required for the ten distinct similarity measures. Fig. S22c provides a visual representation of the results, showcasing the trade-off between accuracy and computational time for each similarity measure. Our findings underscore this trade-off, emphasizing that no single method emerges as the optimal choice simultaneously considering both accuracy and running time. It is worth noting that the computational time requirements across the various similarity measures are comparable, with the slowest measure taking up to six times longer than the fastest one. This indicates that none of the similarity measures exhibit excessive slowness. Importantly, this is a result of our careful selection process, where we prioritize similarity measures based on their performance and computational efficiency.

We have also conducted experiments to assess the impact of excluding slower similarity measures on overall performance. Specifically, we integrate the base results generated by the top five computationally efficient similarity measures, including the Jaccard index, Spearman correlation coefficient, weighted rank correlation, cosine similarity, and Pearson correlation coefficient, and compare them to the results obtained using all ten measures. As illustrated in Fig. S22e, the ensemble results produced by the top five computationally efficient similarity measures do not match the ensemble results achieved by utilizing all ten measures. Furthermore, since the computational time requirements for these various similarity measures are reasonably comparable, we recommend that users utilize all available similarity measures. This approach ensures accurate predictions, enhances model generalization, and results in a manageable increase in running times.

(4) For the MERFISH dataset, the authors specifically selected the "mouse2 slice300" slice from the second mouse. However, to evaluate the generalization ability and robustness of the proposed methods, it would be advantageous for the authors to expand their experiments to include a larger number of slices. This could involve incorporating multiple slices from the same mouse or even different mice. By diversifying the datasets, the findings can be validated across a wider range of samples, thereby enhancing the confidence in the generalizability of the results.
**Response:**

We appreciate the referee’s suggestion to expand the scope of our experiments for the MERFISH dataset. In the revised manuscript, we have applied ENGEP in an additional slice “mouse2-slice50” from the same mouse and two additional slices “mouse1-slice31” and “mouse1-slice313” from the different mouse. The results of these additional three slices are shown in Figs. S5-S7 in Additional file 1. The consistently superior performance underscores the generalizability and robustness of ENGEP.

(5) The authors employed manifold mixup to assign the unmeasured genes into groups categorized as known archetypes or novel archetypes. Based on the definitions, it appears that the values of $g_{ij}$ are random due to the random sampling of $\lambda$ from a uniform distribution between 0 and 1. Consequently, the threshold used to distinguish genes as known or novel archetypes, which is determined by the average likelihood scores of all synthesized genes $g_{ij}$, could potentially be random. This raises the question of whether the threshold would vary significantly with different generations of synthesized genes and if it would consequently affect the partitioning of unmeasured genes into known and novel archetype groups. It would be valuable for the authors to address this concern and investigate the potential impact of different generations of synthesized genes on the threshold and resulting partitioning.

**Response:**

We appreciate the referee’s insightful observation regarding the potential randomness in the threshold defined based on manifold mixup. In response to this concern, we have conducted experiments to study whether the threshold significantly varies with different generations of synthesized genes and if this variability affects the partitioning of unmeasured genes. Specifically, for each dataset, we repeat the threshold calculation process 50 times and record the threshold value for each repetition. To assess the impact of randomness in threshold values on gene partitioning, we use the threshold values obtained from each repeated experiment to partition the genes. We then calculate the Jaccard coefficient between the partitions obtained from each repetition and those obtained using the threshold values as described in the previous paper. The results, illustrated in Fig. R1, indicate that while the threshold values calculated by this method exhibit some degree of randomness, the variation is relatively small, and it does not significantly affect gene partitioning. This suggests that the approach remains robust despite the random element in threshold determination.

It is important to note that, in our revised manuscript, we adopt a new approach to classify unmeasured genes into three distinct groups: those exhibiting a strong association with known patterns, those showing a weak association with known patterns, and those entirely different from the known patterns. This enables a clear differentiation between genes associated with novel patterns and those linked to known patterns, leading to the discovery of valuable insights into entirely new realms of biology. Specifically, after confirming the presence of a multimodality structure in the distribution of likelihood scores, we use the density function in R to estimate the probability distribution of likelihood scores. We then determine the likelihood score at the low peak between the two high peaks, denoted as $s$. Genes are divided into three groups using two thresholds, $s+0.05$ and $s-0.05$. Genes with likelihood scores greater than $s+0.05$ are classified as members of known patterns, while genes with likelihood scores lower than $s-0.05$ are designated as belonging to novel patterns. The remaining genes are considered weakly associated with known patterns. Detailed descriptions of this approach are provided in the “Identification of novel spatial patterns from predicted gene expression” section, and the density distribution of likelihood scores for three spatial datasets is shown in Figs. 3b, 5b, and 7b.

This modification results in a more reliable and interpretable classification of unmeasured genes, aiding in the identification of entirely new spatial patterns that differ from the observed known patterns. We have updated the manuscript accordingly to reflect this modification.

**Fig. R1 Randomness in the threshold for gene partitioning**. The first column illustrates the randomness in the threshold across 50 repetitions of experiments conducted on different spatial datasets. The second column displays the Jaccard coefficient, indicating the similarity between the partitioning of unmeasured genes into novel patterns using the randomly computed threshold in each repetition and the threshold employed in the previous manuscript. Each row corresponds to a different dataset.

**Referee #3:** The authors presented ENGEP, a method that uses multiple reference datasets and parameters to predict the gene expression levels in image-based spatial transcriptomics data. This method appears promising and can potentially be of interest to users. However, there are multiple concerns that need to be addressed.
**Response:**

We deeply appreciate the thoughtful and constructive feedback provided by the referee. Your comments have been instrumental in improving the quality and clarity of our manuscript. We have taken into account each of the concerns raised by the referee and have made revisions and clarifications accordingly.

Major concerns:

1. In Figs. 3d, 5d, and 7d, the authors showed novel archetypes and the cell types they may correspond to. First, the cell types that are colored in these figures seem to be selected cell types. Please clarify how these cell types are selected. Second, since the pattern of these novel archetypes tend to be much noisier than the known archetypes, it is hard to draw correspondence between the novel archetypes and the highlighted cell types. The authors can consider providing quantitative measures to show that the highlighted cell types are the ones that match the novel archetypes better than other cell types.
**Response:**

We appreciate the referee’s feedback on our figures illustrating novel archetypes and their associations with cell types. In our initial submission, we manually selected the cell types displayed in these figures based on identified high-expression regions for each archetype. In response to your valuable suggestion, we have implemented a more systematic and quantitative approach for selecting these cell types, which are associated with spatial patterns (referred to as archetypes in the previous version but now referred to as spatial patterns). Detailed information about this method is provided in Section S3.3 in Additional file 1.

Specifically, we calculate correlations between each cell type and the expression levels of a given spatial pattern. These correlations are used to rank the cell types. Starting with the cell type showing the highest correlation, we iteratively include additional cell types with the highest correlations among those remaining. During this process, we monitor changes in correlation values. When the increase in correlation falls below a user-defined threshold (default threshold set to 1.05), we consider further additions of cell types as not significantly enhancing the correlation. At this point, we identify this set of cell types as closely co-located with the spatial pattern considered.

To provide quantitative measures of the correspondence between the selected cell types and the gene expression patterns of spatial patterns, we calculate cumulative correlations between cell types and spatial patterns. These results are presented in Table S2, Table S4 and Table S6 in Additional file 2 for MERFISH, osmFISH and STARmap, respectively.

Additionally, as pointed out by the referee, it appears that the novel spatial patterns tend to exhibit more noise compared to the known patterns, making it challenging to establish a direct correspondence between these novel patterns and the highlighted cell types. We agree with referee that this lower correspondence between the novel spatial patterns and the selected cell types could be attributed, in part, to potential noise in the predictions. However, a more critical factor contributing to this discrepancy is the distinct cellular composition and spatial distribution associated with these patterns. Notably, the known patterns often co-localize with neuronal cell types exhibiting well-defined cortical layer patterns. In contrast, the novel spatial patterns predominantly co-localize with various non-neuronal cell types that are distributed in a more granular manner. This divergence in cellular composition and spatial organization could be attributed, in part, to the abundance of measured genes serving as markers for neuronal cell types, while marker genes for non-neuronal cells are relatively scarce. These findings strengthen our confidence in the biological relevance of the novel spatial patterns, underscoring their potential to unveil previously unexplored biological insights from measured genes

2. In Figs. 3e, 5e, and 7e, the authors showed Gene Ontology analysis terms of the genes in the novel archetypes. First, the authors did not mention what background gene list was used for GO analysis. In case the complete gene list of the corresponding species is used, the authors should re-do this analysis with the background gene list that contains only the expressed genes in the dataset. Second, the presented GO terms do not sufficiently support the proposed cell types for the novel archetypes. Further discussions with references need to be provided to support the consistency between the top GO terms and the selected cell types that correspond to the novel archetypes.

**Response:**

We appreciate the referee’s valuable feedback regarding the Gene Ontology (GO) analysis in our manuscript. First, in the previous submission, we employed the complete gene list of the corresponding species as the background for GO analysis. In the revised manuscript, we have re-conducted this analysis using a background gene list that exclusively comprises genes expressed in the reference dataset. To achieve this, we assemble the background gene list as the union of genes expressed in all the reference datasets utilized for prediction. To clarify this methodological improvement, we have added the following text in the section titled “Identification of novel spatial patterns from predicted gene expression”: “Finally, we employ Gene Ontology enrichment analysis using the clusterProfiler R package to unveil the biological processes associated with these novel spatial patterns. Notably, for this analysis, the background gene list comprises the union of genes included in the reference datasets.” Detailed results, including the genes within each novel pattern and the enriched biological process-related functions, are presented in Additional file 2: Table S3, Table S5, and Table S7 for MERFISH, osmFISH, and STARmap, respectively.

Second, we have incorporated additional discussions and references to reinforce the consistency between the enriched biological processes and the selected cell types corresponding to the novel patterns. To illustrate, consider novel pattern 1 detected in the MERFISH dataset, which exhibits co-localization with oligodendrocytes. Oligodendrocytes play a pivotal role in the central nervous system by supporting and insulating axons through the formation of protective myelin sheaths [1,2]. Our functional enrichment analysis reveals intricate associations between the genes within novel pattern 1 and various oligodendrocyte-related biological processes, including myelination, ensheathment of neurons, axon ensheathment, oligodendrocyte differentiation, and others. This outcome substantially supports the consistency between the top GO terms and the selected cell types corresponding to the novel patterns. We have elaborated on the consistency between selected cell types and enriched biological process terms, accompanied by pertinent references, in the last paragraphs of the “ENGEP accurately predicts unmeasured genes for MERFISH”, “ENGEP accurately predicts gene expression in unmeasured targets for osmFISH”, and “ENGEP accurately predicts unmeasured gene expression and corrects measured genes for STARmap” sections for the MERFISH, osmFISH, and STARmap datasets, respectively.

Reference

[1] Tomassy GS, Dershowitz LB, Arlotta P. Diversity matters: a revised guide to myelination. Trends Cell Biol. 2016;26:135–47.

[2] Molofsky AV, Krenick R, Ullian E, Tsai H-h, Deneen B, Richardson WD, et al. Astrocytes and disease: a neurodevelopmental perspective. Genes Dev. 2021;26:891-907.

3. In multiple figures where predicted gene expression patterns are compared with ISH images, it is hard to observe the claimed consistency. Examples include Fig. 2d, Fig. 3c-d. The authors should make an effort to make this consistency easier to observe. Some suggestions are: First, the ISH images can be shown in the same direction as the images of predicted gene expression. Second, please add color legends (color code) that shows the correspondence between colors and gene expression for all ISH and predicted gene expression images. Third, highlight certain parts in the images which can help show the consistency.

**Response:**

We would like to express our appreciation for the referee’s valuable suggestions regarding the presentation of predicted gene expression patterns and their comparison with ISH images. We have made the following improvements based on your feedback:

**(1) Orientation of ISH images**: In our previous submission, the orientation of the MERFISH ISH images did not match that of the predicted gene expression patterns. In response to your suggestion, we have adjusted the orientation of ISH images to align with the direction of the predicted gene expression images in all relevant figures.

**(2) Color legends**: We have added color legends (color codes) to all ISH images and gene expression patterns to provide a clear indication of the correspondence between colors and gene expression levels. Deeper colors in ISH images typically represent high expression levels, while lighter colors signify low expression levels. Conversely, in gene expression patterns, deeper colors indicate low expression levels, and bright yellow corresponds to high expression levels.

**(3) Highlighting high-expression areas**: Highlighting high-expression areas in both ISH and gene expression images has been a suggestion we carefully considered. However, we encountered challenges when attempting to apply this approach to genes that do not exhibit clear layer structures, such as *Adarb2* (as shown in Fig. R2). *Adarb2* serves as a marker gene for major subclasses of GABAergic neurons [1] known to have a granular distribution [2], making it challenging to effectively highlight all relevant high-expression points. In these cases, highlighting all high-expression points could result in cluttered images that are difficult to interpret, as the sheer number of highlighted points would obscure the image and reduce overall clarity. While the proposed highlighting approach could provide straightforward visualization for genes with clear layer structures, it becomes less practical for genes with granular distributions. Therefore, considering the trade-off between highlighting and image clarity, and following the approach used in previous studies that compare predicted expression with ISH images without highlighting high-expression areas [2,3], we have decided not to implement this highlighting in our revised figures. Instead, we have included clearer and updated ISH images in Figs. 2, 3, 5, 6, and 7. These improved visuals should assist readers in assessing the consistency between ISH images and predicted gene expression more effectively.

**Fig. R2. Predicted gene expression with highlighted high-expression regions.** This figure corresponds to Fig. 3a, illustrating the predicted expression levels of seven spatially unmeasured genes in MERFISH. In the first row, the expression is predicted by ENGEP, while the second row displays the corresponding ISH images sourced from the Allen Brain Atlas. We have encircled high-expression regions in both the predicted expression images and ISH images using red circles. For genes that exhibit clear layer structures, highlighting the high-expression regions is straightforward. However, for genes that lack distinct layer structures, like *Adarb2* (a marker gene for Vip/Sncg and Lamp5), which are major subclasses of GABAergic neurons known to have a granular distribution, it becomes challenging to highlight all the high-expression points.

Reference

[1] Yao Z, Liu H, Xie F, Fischer S, Adkins RS, Aldridge AI, et al. A transcriptomic and epigenomic cell atlas of the mouse primary motor cortex. Nature. 2021;598:103–10.

[2] Biancalani T, Scalia G, Buffoni L, Avasthi R, Lu Z, Sanger A, et al. Deep learning and alignment of spatially resolved single-cell transcriptomes with Tangram. Nat Methods. 2021;18:1352–62.

[3] Abdelaal T, Mourragui S, Mahfouz A, Reinders MJT. SpaGE: Spatial gene enhancement using scRNA-seq. Nucleic Acids Res. 2020;48:e107–e.

4. In order to use this tool, users need to collect multiple reference datasets. One question is how many datasets are sufficient. Is it the more the better, or can it reach saturation at some point? Please provide discussion on this which can be helpful for users.

**Response:**

We appreciate your thoughtful consideration of the usability of our tool and your question about the sufficiency of reference datasets. In our revised manuscript, we have conducted new experiments to analyze the effect of the number of references on the outcome of ENGEP, which can provide valuable insights for users (Additional file 1: Section S3.1).

Specifically, we run ENGEP with different numbers of sub-references (partitioning the original reference datasets) and use cross-validation to evaluate the performance. The results, shown in Fig. S21a, reveal a nuanced pattern: datasets with a substantial number of sub-references, such as MERFISH, initially exhibit performance improvement, but this improvement saturates over time. In contrast, datasets with fewer sub-references, like osmFISH and STARmap, consistently benefit from an increasing number of sub-references (Fig. S21b, c).

Therefore, our recommendation to users is to leverage all available reference datasets, especially when the number of reference datasets is limited. This approach consistently enhances the method’s performance. However, for datasets that already include a wide array of references, it is important to be aware that there is a potential for performance saturation, indicating that computational time can be economized by training the model with a reduced number of reference datasets. It is important to note that the threshold for performance saturation may vary across different datasets, making it challenging to determine an optimal number of references universally. To address this, we plan to explore statistical learning theory in future research to better understand the interplay between performance and the number of references, providing more guidance for users.

In the “Discussion” section of our manuscript, we have also added the following text to further clarify this issue: “We further delve into the impact of the number of references on outcomes through a series of experiments (Additional file 1: Section S3.1). Our findings reveal that datasets with fewer references (e.g., osmFISH and STARmap) invariably benefit from an increasing number of references. Conversely, datasets with a substantial number of references (e.g., MERFISH) exhibit an initial performance ascent followed by saturation (Additional file 1: Fig. S21).”

5. There is no discussion on how to set parameters for ENGEP or which hyper parameters are used to generate the presented results. The parameters include those used in pre-processing steps. Please add this discussion to guide users for parameter selection.

**Response:**

We appreciate your suggestion. ENGEP is designed to be robust and user-friendly, which means it does not require meticulous parameter tuning. However, we understand the importance of providing guidance on parameter selection for users who may want to fine-tune the tool to their specific datasets.

In response to your feedback, we have included a comprehensive clarification of the parameter settings for ENGEP in the revised manuscript. This clarification encompasses the parameters used in preprocessing steps, including data normalization. It also addresses parameters related to the maximum number of cells ($n_{0}$) within each sub-reference dataset when partitioning extensive reference datasets, as well as the array of$k$values used in KNN regression. This additional information is intended to provide users with clear guidance for selecting appropriate parameter settings tailored to their specific applications. For detail, please refer to the section titled “Parameter Settings for ENGEP”.

6. Lines 323-326: the explanation of why no genes are assigned to known archetype 5 is not supported. To verify that this is indeed due to the low gene detection sensitivity, the authors can compare the detection sensitivity of genes in known archetype 5 and genes in other known archetypes.

**Response:**

We appreciate the opportunity to clarify the text and address the referee’s concern. In our previous manuscript, we made a misleading statement regarding why no genes are assigned to known archetype 5. To investigate this issue, we have conducted a comparison of gene detection sensitivity between genes in known archetype 5 and genes in other known archetypes. This comparison revealed that the mean detection sensitivity of archetype 5 is not the lowest among the known archetypes. To further understand why no genes are assigned to known archetype 5, we visualized the expression patterns of all genes encompassed by this archetype. Our observations indicate that the gene expression patterns within archetype 5 do not exhibit significant similarity with each other (Fig. R3).

To address this issue and improve the identification of spatial patterns, we have adopted a different approach inspired by [1]. Specifically, we calculate a Spatial Cross-Correlation Index (SCI) for genes to construct a weighted gene network. We then apply Louvain clustering on this gene network to identify distinct spatial patterns. This revised methodology is detailed in the “Identification of known spatial patterns from measured gene expression” and “Identification of novel spatial patterns from predicted gene expression” sections.

Furthermore, we have updated our approach for classifying unmeasured genes. In the previous manuscript, unmeasured genes were grouped into two categories: those belonging to known patterns and those belonging to novel patterns. In the revised version, we classify unmeasured genes into three distinct groups: those with a strong association with known patterns, those with a weak association with known patterns, and those entirely different from the known patterns. This refined classification system uses two thresholds to categorize genes, allowing for a clear differentiation between genes associated with novel patterns and those linked to known patterns. This enhances our ability to uncover novel insights into previously unexplored biological phenomena. Please refer to the section titled “Identification of novel spatial patterns from predicted gene expression” for more details.

Despite these changes, we still find cases where there are known patterns with no genes strongly associated with them. For example, known pattern 5 in MERFISH (including three genes), known patterns 4, 5, and 6 in osmFISH (each including only one gene), and known pattern 5 in STARmap (including four genes). Several factors may contribute to this absence of strong associations. First, these patterns consist of fewer genes compared to patterns with strong gene associations, suggesting they may be less biologically significant. Second, upon visualizing the expression patterns of the genes within these patterns, we observe that their measured expression levels do not exhibit clear layer structures as seen in other known patterns. Additionally, they do not closely match the ISH images, indicating the presence of latent noise in the measured values. This observation provides a potential explanation for the lack of predicted genes associated with these patterns.

We hope these explanations and methodological improvements address the concerns and provide a clearer understanding of our approach to identifying spatial patterns in spatial transcriptomics data.

**Fig. R3. Genes in archetype 5 in the previous manuscript.** This figure illustrates the measured gene expression levels in archetype 5 as presented in the previous manuscript.

Reference

[1] Miller BF, Bambah-Mukku D, Dulac C, Zhuang X, Fan J. Characterizing spatial gene expression heterogeneity in spatially resolved single-cell transcriptomic data with nonuniform cellular densities. Genome Res. 2021;31:1843–55.

Minor:

Fig. S1: dimodality → bimodality

**Response:**

We correct the caption in Fig. S1 to replace “dimodality” with the correct term “bimodality”. We also update Fig. S1 to fit our new experiment procedure.

Line 300: "a major cell type" - please specify the cell type.

**Response:**

In the revised manuscript, we have specified the cell type, which is oligodendrocytes, to provide more clarity.

Fig. 2c: nagative → negative

**Response:**

We correct the caption in Fig. 2c to replace “nagative” with the correct term “negative”.

**Second round of review**

**Reviewer 2**

The authors addressed all my concerns in a satisfactory manner. I am willing to see its publication in the current version.

**Reviewer 3**

I appreciate the efforts the authors made to address my concerns. After this revision, I still have the following concerns:

1. In Figure 5, the pattern of novel pattern 2 does not compare well with osmFISH.

2. There is no discussion on the GO terms of novel pattern 2 (in Fig. 5) and it is hard to see how the enriched terms in Fig. 5e support the inhibitory neuron functions.

3. Regarding facilitating comparison between gene expression patterns and ISH images: In my previous comment #3, I used Fig. 2 and Fig. 3 as examples of those cases where it’s hard to compare with ISH images. The same problem exist in some other figures, like Fig. 5. It appears that in Fig. 5 the ISH images are not rotated.

Other comments:

It would be very helpful if the authors can point to exact line numbers of changes made in the manuscript for each response. It will also help the reviewers to identify changes in the manuscript if the changes in the manuscript and supplementary files are highlighted, for example, using red fonts.

**Authors Response**

**Point-by-point responses to the reviewers’ comments:**

**Reviewer #3:**

I appreciate the efforts the authors made to address my concerns. After this revision, I still have the following concerns:
**Response:**

Thank you for your continued engagement with our manuscript and for acknowledging our efforts to address your previous concerns. We highly value your feedback and remain committed to further addressing any remaining concerns you may have.

1. In Figure 5, the pattern of novel pattern 2 does not compare well with osmFISH.

**Response:**
We appreciate the opportunity to provide further clarification regarding the pattern of novel pattern 2 in osmFISH (Figure 5). Firstly, we have horizontally mirrored the respective ISH images to ensure consistent orientation with the predicted gene expression patterns, aligning with suggestions from both the editor and referee.

Upon mirroring the ISH images, it is evident that both the identified novel pattern 2 and the corresponding ISH image exhibit a granular structure, indicating an alignment between the pattern of novel pattern 2 and the ISH image.

Thirdly, utilizing the methods outlined in Additional file 1 (Section S3.3), we have identified several cell types co-localizing with this novel pattern. These types comprise inhibitory neuron cell types (Additional file 2: Table S4). Specifically, they encompass Inhibitory CP, Inhibitory Crhbp, Inhibitory Kcnip2, and Inhibitory IC. Notably, these cell types are granularly distributed, consistent with the spatial arrangement of inhibitory neuronal cell types provided in the osmFISH manuscript (Figure R1).

Moreover, we have expanded the discussion on the GO terms associated with novel pattern 2 and elucidated how the enriched terms in Figure 5e substantiate the functions related to inhibitory neurons. For further details, please refer to our response to the second comment.

**Figure R1 depicts the characterization of novel pattern 2 in osmFISH.** a. Illustrates the expression pattern of novel pattern 2 in osmFISH. b. Demonstrates the co-localization of novel pattern 2 with inhibitory neuron cell types. c. Shows the spatial distribution of inhibitory neuron cell types, as outlined in the osmFISH manuscript available at <http://linnarssonlab.org/osmFISH/clusters/>.

2. There is no discussion on the GO terms of novel pattern 2 (in Figure 5) and it is hard to see how the enriched terms in Figure 5e support the inhibitory neuron functions.

**Response:**

We value the reviewer’s insightful feedback and aim to address the query comprehensively. In Figure 5e, we conducted functional enrichment analysis on genes associated with novel pattern 2. The top enriched biological processes are associated with cell adhesion molecules (such as cell-cell adhesion via plasma-membrane adhesion molecules, and homophilic cell adhesion via plasma membrane adhesion molecules) and acetylcholine-related processes, like the acetylcholine receptor signaling pathway, response to acetylcholine, and cellular response to acetylcholine.

Cell adhesion molecules play a crucial role in cell-cell interactions within the nervous system. Inhibitory neurons, responsible for regulating neural activity, express various cell adhesion molecules, facilitating the establishment and maintenance of connections between inhibitory neurons and their targets. This aids in proper neural circuit function and inhibition of excessive neuronal activity. Additionally, existing literature suggests the significant role of acetylcholine in modulating inhibitory neuron activity, especially during motor learning in the cerebral cortex. Notably, the cell types co-localizing with novel pattern 2 predominantly comprise inhibitory neuron cell types.

Furthermore, as demonstrated in Additional file 2: Table S5, we observed additional enrichment in biological processes associated with GABAergic functions. GABA, a primary neurotransmitter, predominantly serves an inhibitory modulatory role in the central nervous system.

To provide further clarification, in the “ENGEP accurately predicts gene expression in unmeasured targets for osmFISH” section, we further elaborate on this association as follows:

“*On the other hand, novel pattern 2 co-localizes with various inhibitory neurons (e.g., Inhibitory CP, Inhibitory Crhbp, Inhibitory Kcnip2, and Inhibitory IC), which play a fundamental role in maintaining the stability and functionality of the nervous system [34]. Its member genes exhibit enrichment across a range of biological processes, particularly those linked to cell interactions, neural development, and neurotransmission. Notably, the primary enriched processes, shown in Fig. 5e, largely revolve around cell adhesion molecules and acetylcholine. Cell adhesion molecules are instrumental in neural interactions, being expressed by inhibitory neurons to establish and maintain crucial connections necessary for proper neural function and regulation [35]. The influence of acetylcholine in modulating the activity of inhibitory neurons, especially during cerebral cortex functions like motor learning, is underscored by the significant enrichment observed in acetylcholine-related processes [36]. These findings underscore the importance of these novel patterns in untangling complex spatial gene expression and biological processes in the nervous system.”*

3. Regarding facilitating comparison between gene expression patterns and ISH images: In my previous comment #3, I used Figure 2 and Figure 3 as examples of those cases where it’s hard to compare with ISH images. The same problem exists in some other figures, like Figure 5. It appears that in Figure 5 the ISH images are not rotated.

**Response:**

We highly value your thorough review of our manuscript and recognize the challenge in comparing gene expression patterns with ISH images, notably observed in Figures 2, 3, and 5. To address this concern, we have taken specific measures. Firstly, in line with the editor's guidance, we have organized the expression patterns and ISH images of genes in MERFISH to align with the correct cortical order in Figures 2 and 3.

Moreover, in Figure 5, we have horizontally mirrored the ISH images to ensure a consistent orientation with the predicted gene expression patterns. These adjustments aim to facilitate a more coherent comparison between gene expression patterns and ISH images. With these revisions, we have ensured that both gene expression patterns and ISH images can be effectively compared in the same orientation across all relevant Figures (Figures 2, 3, 5, 6, and 7).

Other comments:

It would be very helpful if the authors can point to exact line numbers of changes made in the manuscript for each response. It will also help the reviewers to identify changes in the manuscript if the changes in the manuscript and supplementary files are highlighted, for example, using red fonts.

**Response:**

Thank you for your valuable feedback. We understand the importance of clarity in tracking changes made during the revision process. Your suggestion regarding pointing out exact line numbers of changes in the manuscript and highlighting modifications in both the manuscript and supplementary files is duly noted.

However, in this round of revision, we were instructed by the editor to remove the line numbers from the manuscript. Consequently, we have not included specific line numbers in the manuscript to point out the changes made for each response.

We have aimed to address your concerns by highlighting all modifications in the manuscript. In this revision, we have ensured that all changes within the manuscript are appropriately highlighted in a separate file, facilitating easier identification.
